# Evidence on Strategies for Integrating Nutrition Interventions with Health and Immunization Systems in Conflict-affected Areas of Low- and Lower-middle-income Settings—A Systematic Review

**DOI:** 10.1093/nutrit/nuaf031

**Published:** 2025-04-12

**Authors:** Amira M Khan, Bianca Carducci, Oviya Muralidharan, Zulfiqar A Bhutta

**Affiliations:** Centre for Global Child Health, Hospital for Sick Children; Peter Gilgan Centre for Research, and Learning (PGCRL), Toronto, ON M5G 0A4, Canada; Department of Nutritional Sciences, University of Toronto, Faculty of Medicine, University of Toronto, Toronto, ON M5S 1A8, Canada; Columbia Climate School, Columbia University, New York, NY 10027, United States; Centre for Global Child Health, Hospital for Sick Children; Peter Gilgan Centre for Research, and Learning (PGCRL), Toronto, ON M5G 0A4, Canada; Centre for Global Child Health, Hospital for Sick Children; Peter Gilgan Centre for Research, and Learning (PGCRL), Toronto, ON M5G 0A4, Canada; Department of Nutritional Sciences, University of Toronto, Faculty of Medicine, University of Toronto, Toronto, ON M5S 1A8, Canada; Centre of Excellence in Women, and Child Health, Aga Khan University, Karachi 74800, Pakistan; Dalla Lana School of Public, Health University of Toronto Health Sciences Building, Toronto, ON M5T 3M7, Canada

**Keywords:** under-five nutrition, maternal nutrition, conflict, integrated strategies, under-five health, maternal health, nutrition-specific intervention, nutrition-sensitive intervention

## Abstract

**Context:**

Pervasive conflict and war adversely affect a nation’s sustainable development. Health and health systems deteriorate, causing long-lasting impacts on diets and nutrition. For the most vulnerable, integrated models of delivery of essential nutrition interventions are critical for the efficiency and sustainability of programs in these settings.

**Objective:**

The objective of this systematic review was to provide evidence on coverage, utilization, and impact of integrated programs in conflict-affected, low- and middle-income countries (LMICs).

**Data Sources:**

A database search was conducted in MEDLINE, Embase, CINAHL, and CENTRAL from January 1, 2000 to February 14, 2024. Gray literature was also reviewed.

**Data Extraction:**

Quantitative and qualitative studies, including observational or intervention designs, and reviews and program evaluations conducted in LMICs, focusing on women (≥19 years) and children (0-19 years) were included. Data extraction and risk-of-bias assessment were conducted independently by 2 investigators using a standardized tool.

**Data Analysis:**

In total, 93 studies (103 reports) were included from 25 countries, including 32 unique gray literature records. The conflict-affected regions included South-East Asia (n = 27), Eastern-Mediterranean (n = 8), Africa (n = 58), and the Americas (n = 1). The review distinguished programs based on nutrition-specific, nutrition-sensitive, and health components. Although the coverage and utilization of integrated nutrition and health programs has been substantial, the impact of the strategies on health and nutrition has been limited. The meta-analysis found no significant differences in rates of wasting among children under 5 years; however, it showed that children who received an integrated strategy experienced a 28% lower risk of underweight (P = .007) and a 12% reduced risk of stunting (P = .05) compared with those who did not.

**Conclusion:**

This review has provided an in-depth insight into integrated nutrition and health strategies in conflict-affected settings, identifying key facilitators and barriers that can inform future policy and program design. Integrating nutrition programs into health systems and enhancing government and community ownership could enhance efficiency and sustainability, given challenging environments.

**Systematic Review Registration:**

PROSPERO registration No. CRD42022373993.

## INTRODUCTION

Conflict has been identified as one of the leading drivers globally of acute food insecurity, secondary only to economic shocks, with nearly 120 million people acutely food insecure in conflict-affected countries. The State of Food Security and Nutrition has identified conflict, climate change, rising food prices, and the persisting effects of the pandemic as the main threats to achieving the 2030 Sustainable Development Goals, especially nutrition-related targets.^1^^,^^2^

The past 2 decades have seen an evolution of armed disputes and violence, with nearly one-quarter of the world’s population living in conflict-affected areas. More recently, there has been a staggering increase in conflict fatalities, with more than 237 000 in 2022, an alarming 97% increase from 2021.^3^ Acute and chronic conflict has far-reaching impacts on both health systems and the physical and mental health of communities, with women and children facing the greatest risk.^4^

Importantly, most conflict-affected regions are impacted by complex crises, leading to fragile health systems, disrupted relief activities, and severely limited access to maternal and child health (MNCH) care. Nearly 80% of stunted children live in areas impacted by some form of violence or conflict.^5^ Further, disrupted supply chains and food systems often lead to acute food crises, exacerbating food and economic insecurity and malnutrition, ultimately impacting breastfeeding rates^6^ and optimal complementary feeding.^7^ Evidence indicates that only half of the children receive early initiation of breastfeeding, and barely a quarter are exclusively breastfed, in conflict settings.^8^ Because of these compounding effects, the need for comprehensive service delivery including not only basic primary healthcare services but also nutrition, immunization, and mental health services, that are delivered efficiently and cost-effectively is of utmost importance.

Experts recognize that delivering strategies with a siloed approach, such as food assistance or implementing vertical health programs, is unsustainable and less effective. Integrated strategies may be valuable in improving maternal and under-5 nutrition, health, and immunization services in such contexts. The World Health Organization describes integrated health services as those that are “managed and delivered” so that the community receives a continuum of preventive, curative, and rehabilitative services delivered “across different levels and sites of care within and beyond the health sector, according to the needs throughout the life course.”^9^ Although integrated health services may be of different types with varying operationalization styles, the principle largely remains the same: Providing preventive and curative health and health-related interventions cohesively.

A knowledge gap exists regarding integrated nutrition and health strategies and programs, in the context of conflict in low- and middle-income countries (LMICs). Much of the existing literature focuses on emergency maternal and child health interventions in crises,^10–13^ but evidence on the uptake and impact of integrated strategies, including those for under-5 nutrition, in these contexts, is lacking. A review^13^ focusing on nutrition interventions in conflict settings also highlighted the lack of data on the coverage or impact of nutrition interventions, while calling for evidence generation on multisectoral and integrated programming in such contexts.

The overarching objective of this systematic review was to provide evidence relating to the coverage and impact of integrated programs where nutrition-specific interventions are delivered with nutrition-sensitive and/or health interventions in conflict-affected, LMICs.

The more specific objectives of the review were to:

1. Assess the effectiveness of integrated delivery platforms on nutrition in conflict-affected LMIC settings.
2. Examine the utilization and coverage of the integrated nutrition and health programs in these contexts.
3. Document barriers and facilitators to the implementation and uptake of these integrated programs

## METHODS

The protocol for this review is registered with PROSPERO: CRD42022373993, and the review followed the Preferred Reporting Items for Systematic Reviews and Meta-Analyses (PRISMA) guidelines.

### Search Strategy

A systematic and comprehensive search was conducted on December 9, 2022, for published and gray literature published from January 1, 2000 to current in MEDLINE, Embase, and CINAHL using the OVID and EBSCOhost interfaces. Additionally, the Cochrane Central Register of Controlled Trials was searched. An updated search was conducted on February 14, 2024. The sets of search terms were related to women of reproductive age and children accessing nutrition-specific interventions via integrated strategies during or within 5 years of the end of a conflict. The search strategy is provided in Table S1. For the gray literature, 11 organizational websites and databases were searched using the identified keywords, searching for reports from January 1, 2012 onwards. Websites included Emergency Nutrition Network, International Committee of the Red Cross, International Rescue Committee, Médecins Sans Frontières, Save the Children, United Nations High Commission for Refugees, World Vision, United Nations Children’s Fund, World Bank, World Food Programme, and ReliefWeb.

### Eligibility Criteria

We included studies published in the English language, with the following designs: Quantitative and qualitative studies with an observational study design (cross-sectional, cohort, case–control studies, ecological), intervention study design (randomized controlled trials [RCTs], quasi-experimental), reviews (systematic, literature, scoping), or program evaluations. Animal and One Health studies, conference abstracts, study protocols, and letters/comments were excluded (Table 1).

**Table 1.** PICOS—Inclusion and Exclusion Criteria

Element	Inclusion criteria	Exclusion criteria
**Population**	Integrated strategies targeting women (≥19 years) and children (0-19 years) living in low-income countries and lower-middle-income countries (according to the World Bank definition)	Integrated strategies that do not include women (≥19 years) and/or children (0-19 years) in the target group Not a conflict-affected or post-conflict setting Upper-middle, and high-income countries
**Intervention**	The following integrated strategies were included: Nutrition-specific + Health Nutrition-specific + Nutrition-sensitive Nutrition-specific + Nutrition-sensitive + Health	Studies without nutrition-specific interventions integrated with nutrition-sensitive and/or health service delivery strategies during conflict or post-conflict–affected contexts
**Comparator**	No intervention or business as usual	
**Outcomes**		
***Primary outcomes***		
**Coverage** **Utilization** **Impact**	Coverage of intervention in the target population Examples: Coverage of vitamin A supplementation Proportion of the population provided with food rations Uptake of intervention by the target population Examples: Number of internally displaced persons who accessed services of the camp clinic Number of infants brought to the baby-friendly space Measurable impact of the intervention on the target population Examples: Reducing in wasting in under-5 children Children (6-9 months) fed adequate dietary diversity, appropriate meal amount, and a minimum acceptable diet	
***Secondary outcomes***		
**Facilitators and barriers**	Factors that enabled and helped deliver integrated strategies Examples: Close coordination with government partners Microplans for all interventions Factors that impeded and hindered the delivery of integrated strategies Examples: Logistical challenges Lack of community engagement	
**Study design**	Original research Case studies Expert consensus Correspondence, commentary, opinions, or editorials Systematic, scoping, or rapid reviews Research letters Gray literature	Conference proceedings and posters Author’s replies Research highlights News or media watch

#### Population and Setting

Literature focusing on women (>19 years) and children and adolescents (0–19 years) were included. Conflict- and post-conflict-affected LMIC settings were included. The list of eligible countries is provided in Table S2. A conflict-affected setting was defined as per the Uppsala Conflict Data Program (ie, at least 25 conflict-related deaths per year in a specific country), while post-conflict settings were considered as those with cessation of conflict within the last 5 years.^13^ Low-income and lower-middle-income countries were based on 2023 World Bank country economy classifications.^14^ Eligible affected populations included “forcibly displaced, including, refugees, asylum seekers, and internally displaced people or not displaced but living at increased risk of direct or indirect mortality and morbidity from nearby armed conflicts.”^15^

#### Interventions

Integrated strategies were defined as those that delivered nutrition-specific interventions integrated with existing health systems and/or packaged delivery of interventions with nutrition-specific interventions incorporated with health and/or nutrition-sensitive interventions for a particular population group.^16^ This resulted in 3 types of integrated strategies: (1) Nutrition-specific and health; (2) Nutrition-specific and nutrition-sensitive; and (3) Nutrition-specific, nutrition-sensitive, and health. Acknowledging the new framework for effective nutrition interventions,^17^ our review mapped interventions in the following way:

1. Nutrition-specific interventions equate to direct health and direct other sectoral interventions, including the immediate determinants of nutrition, such as micronutrient supplementation, optimal infant and young child feeding (IYCF), and management of severe acute malnutrition (SAM).
2. Nutrition-sensitive interventions equate to indirect other sectoral interventions, including those impacting the underlying determinants of nutrition, such as water, sanitation and hygiene (WASH) strategies, cash transfers, and early childhood development.
3. Health interventions equate to indirect health sector interventions, including strategies directly impacting health outcomes, such as immunization, disease management, and health promotion.

Studies and gray literature focusing on stand-alone interventions that were not integrated as outlined in our protocol were not included. Interventions not targeting women (>19 years) and/or children (0–19 years) were not included.

#### Comparator

In most studies, the comparators were either no interventions or routine interventions being provided as per usual.

#### Outcomes

Primary outcomes included coverage, utilization, and impact of the integrated strategies, and the secondary outcomes included barriers to and facilitators of the strategy and cost-effectiveness (Table 1).

### Data Synthesis

All unique records identified were uploaded to Covidence. Once duplicates were removed, an assessment of study eligibility was initiated independently by 2 authors (A.K., B.C.), with title and abstract screening of retrieved records. Full texts of potentially eligible studies were uploaded into Covidence and screened by 2 reviewers (A.K., B.C.), using the following exclusion criteria; incorrect setting, incorrect intervention, incorrect study design, and incorrect outcomes. Disagreements were resolved by discussion and a third independent reviewer was engaged if consensus was not reached. Data extraction from eligible studies was conducted by 2 reviewers (A.K., O.M.) as per the following domains: General information, study setting and population, conflict details, type of integration and intervention details, primary outcomes, and secondary outcomes. Data was matched by 2 reviewers with conflicts resolved by a third reviewer (B.C.).

### Statistical Analysis

A meta-analysis was conducted for primary outcomes when sufficient articles were reporting an outcome (*N* ≥ 3). Revman 5.4^18^ was used to generate pooled estimates and corresponding forest plots. If articles reported incidence data, the Mantel–Hanzel (M–H) method was used to estimate effect size. If effect estimates were reported, the Inverse-Variance (IV) approach was used to calculate pooled effect. For dichotomous data, an odds ratio (OR) and 95% CI (CI) were computed. Adjusted odds ratios (aORs) were used to account for effects of clustering and baseline demographics in these trials. Heterogeneity was estimated by calculating the *I*^2^ statistic.

Data related to barriers and facilitators was imported into NVivo 14,^19^ a qualitative analysis software, and a thematic analysis was undertaken using an inductive approach. Identification of barriers and facilitators was constructed by reviewer-generated codes, which were then grouped into overarching categories, leading to the emergence of broader thematic patterns.

### Quality of Evidence

The quality of the studies was assessed using specific tools according to study design: RCTs using Cochrane’s Risk of Bias Tool (ROB-2)^20^; quasi-experimental using Cochrane’s ROBINS-I tool^21^; cross-sectional analytical studies using the National Institutes of Health Quality Assessment Tool for Observational Cohort and Cross-sectional Studies^22^; cross-sectional descriptive studies using the JBI Critical Appraisal Checklist for studies reporting prevalence data.^23^ Scores were assigned by 2 independent reviewers (A.K., O.M.). Each study was assigned an overall quality score and was classified as low, low-medium, medium, medium-high, and high. Scores were compared and a final score was determined. In the event of a conflict, a third reviewer (B.C.) was consulted.

### Ethics Approval

Ethics approval was not required, as this paper is a systematic review of publicly available, published literature.

## RESULTS

### Summary of Results

**Figure 1.**
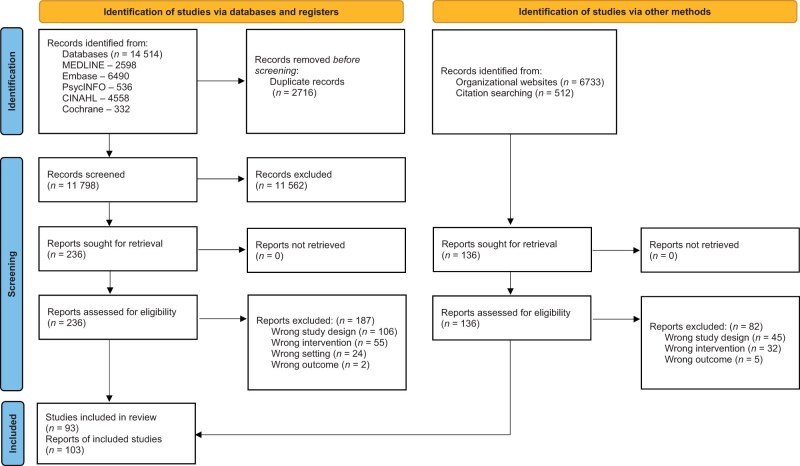
PRISMA Flow Diagram

A total of 14 514 records were identified from the database search. After removing duplicates, the titles and abstracts of 11 798 records were screened, and 236 were included for full-text screening. Reasons for exclusion included wrong study design (*n* = 106), wrong intervention (*n* = 55), wrong setting (*n* = 24), and wrong outcomes (*n* = 2). Data was extracted from 44 peer-reviewed studies (49 reports)^24–70^ that met the inclusion criteria. The updated search in February 2024 identified 2 new trials.^71^^,^^72^ Additionally, 15 primary studies (20 reports)^73–87^ were identified using the snowballing method. Finally, 32 unique gray literature records^88–122^ were identified from organizational websites. In total, 101 reports on 93 studies were included in this review. The PRISMA flow diagram is shown in Figure 1. All programs categorized according to method of integration and country are listed chronologically in Table S3.

### Settings

**Figure 2.**
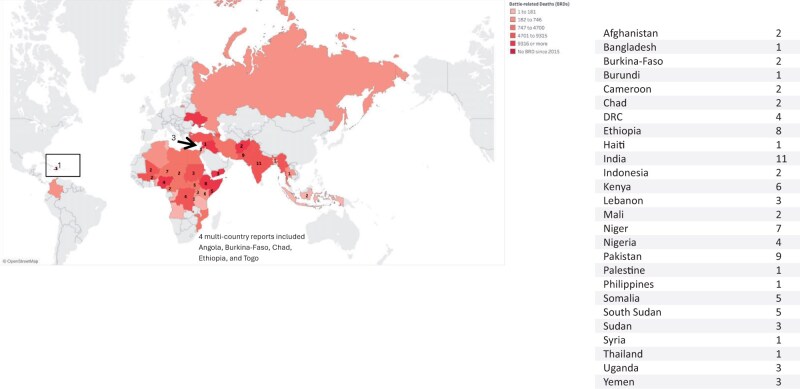
Number of Studies Included Per LMIC Affected by Conflict (Source for Battle-related deaths:^123^)

Table S4 lists the characteristics of the included studies implemented across 25 countries. The conflict-affected regions included Africa (*n* = 58), South-East Asia (*n* = 27), Eastern-Mediterranean (*n* = 8), and the Americas (*n* = 1) (Figure 2).^123^

Most programs were implemented in conflict settings, except 10 studies that focused on refugees from the Central African Republic,^42^^,^^91^ Somalia,^105^ Sudan and Democratic Republic of Congo,^26^^,^^64^ Myanmar,^38^^,^^49^ Syria,^34^^,^^44^ and the State of Palestine^46^ hosted in neighboring countries. All studies included strategies for children and/or adolescents, with some also focusing on caregivers, usually women. Programs served internally displaced persons (IDPs), refugees, and communities who are native to the region. Some studies selected target households based on socioeconomic status, focusing on “poor” or “very poor” households.

### Study Designs

The number of peer-reviewed and gray literature records included by year of publication is shown in Figure S1. Most included articles reported on short-term programs (<1 year)^24^^,^^31^^,^^37^^,^^40^^,^^41^^,^^46^^,^^49^^,^^52^^,^^53^^,^^56^^,^^58^^,^^61^^,^^63^^,^^64^^,^^69^^,^^76^^,^^80^^,^^81^^,^^85^; however 8 studies assessed longer programs (operational for ≥4 years).^26^^,^^30^^,^^34^^,^^51^^,^^54^^,^^73^^,^^74^^,^^87^ Eleven articles reported follow-up of participants after 12 months,^25^^,^^39^^,^^44^^,^^48^^,^^60^^,^^71^^,^^75^^,^^77^^,^^78^^,^^86^^,^^120^ and 3 articles each after 28 months,^32^^,^^33^^,^^72^ and after 33 months.^79^^,^^82^^,^^86^ Multiple study designs were used: 26 were experimental, including 16 RCTs (15 cluster-randomized^24^^,^^25^^,^^29^^,^^47^^,^^48^^,^^50^^,^^54^^,^^70–72^^,^^79^^,^^86^^,^^87^^,^^120^^,^^124^ and 1 trial with both cluster and individual randomization),^78^ 9 quasi-experimental studies,^28^^,^^32^^,^^37^^,^^44^^,^^52^^,^^63^^,^^83^^,^^84^ and 1 case–control study.^80^ Thirty-five were observational studies, including 16 program evaluations,^26^^,^^30^^,^^36^^,^^39^^,^^40^^,^^57^^,^^58^^,^^61^^,^^69^^,^^73–77^^,^^82^ 12 program descriptions,^31^^,^^33^^,^^35^^,^^38^^,^^41–43^^,^^49^^,^^51^^,^^56^^,^^60^^,^^81^ 6 cross-sectional analytical studies,^34^^,^^53^^,^^55^^,^^59^^,^^62^^,^^64^ and 1 cohort study.^85^ Ten were secondary reports or commentaries of included studies.^27^^,^^65–68^^,^^114^^,^^124–127^ Using quality appraisal tools, all 12 descriptive studies were assessed as being of sufficient quality to be included. The remaining observational and experimental studies were evaluated. Seven studies were graded as being of low quality,^30^^,^^55^^,^^59^^,^^64^^,^^67^^,^^69^^,^^120^ 30 were graded as being of moderate quality,^26^^,^^28^^,^^32^^,^^34^^,^^36^^,^^37^^,^^39^^,^^40^^,^^44–46^^,^^50^^,^^52^^,^^53^^,^^57^^,^^58^^,^^61–63^^,^^70^^,^^72^^,^^74–77^^,^^79–81^^,^^83^^,^^84^ and 12 were graded as being of high quality.^24^^,^^25^^,^^29^^,^^47^^,^^48^^,^^71^^,^^73^^,^^78^^,^^85–87^^,^^124^ Broadly, studies were downgraded due to attrition bias from loss to follow-up, bias due to lack of blinding of participants, investigators, and/or outcome assessors, and insufficient information on the randomization process. Figures S2–S5 show the detailed quality assessment per study.

From the gray literature sources, 8 program reports,^90^^,^^94^^,^^96^^,^^97^^,^^100^^,^^104^^,^^110^^,^^112^ 6 field reports,^91^^,^^92^^,^^99^^,^^105^^,^^111^^,^^115^ 6 program evaluations,^93^^,^^103^^,^^107^^,^^109^^,^^113^^,^^119^ 6 summaries of original research,^88^^,^^89^^,^^102^^,^^106^^,^^114^^,^^116^ 3 operational updates of evolving humanitarian situations,^98^^,^^117^^,^^118^ as well as, a fiscal year report,^108^ technical brief,^101^ and appeal for funds^95^ were included.

Forty-three studies used a nutrition-specific, nutrition-sensitive, and health-integrative strategy,^25^^,^^26^^,^^31^^,^^33^^,^^35–38^^,^^40^^,^^42–44^^,^^46^^,^^48^^,^^50^^,^^54^^,^^70^^,^^71^^,^^73^^,^^74^^,^^77^^,^^79^^,^^80^^,^^82^^,^^86^^,^^88^^,^^89^^,^^91–93^^,^^95^^,^^97–100^^,^^103^^,^^106–108^^,^^113^^,^^114^^,^^118^^,^^119^ 34 studies used a nutrition-specific and health-integrative strategy,^14^^,^^30^^,^^32^^,^^34^^,^^39^^,^^41^^,^^45^^,^^51^^,^^53^^,^^59–62^^,^^64^^,^^69^^,^^75^^,^^76^^,^^81^^,^^83–85^^,^^87^^,^^90^^,^^94^^,^^96^^,^^101^^,^^102^^,^^105^^,^^110–112^^,^^116^^,^^117^^,^^120^^,^^124^ and 14 studies used a nutrition-specific and nutrition-sensitive integrative strategy.^24^^,^^28^^,^^29^^,^^47^^,^^49^^,^^52^^,^^55^^,^^56^^,^^58^^,^^63^^,^^72^^,^^78^^,^^109^^,^^115^

Nutrition-specific activities most frequently included micronutrient supplementation (*n* = 20),^39^^,^^45^^,^^73^^,^^78^^,^^79^^,^^81^^,^^83^^,^^84^^,^^86^^,^^88^^,^^90^^,^^91^^,^^103^^,^^113^ IYCF counseling and support (*n* = 18),^26^^,^^28^^,^^32^^,^^34^^,^^38^^,^^42^^,^^47^^,^^55^^,^^70^^,^^80^^,^^81^^,^^84^^,^^89^^,^^90^^,^^99^^,^^111^^,^^115^^,^^120^ treatment of acute malnutrition by therapeutic and targeted supplementary feeding (*n* = 14),^26^^,^^31^^,^^47^^,^^53^^,^^54^^,^^58^^,^^62^^,^^64^^,^^77^^,^^80^^,^^85^^,^^90^^,^^92^^,^^103^ and general food distributions (*n* = 7).^24^^,^^26^^,^^31^^,^^54^^,^^56^^,^^91^^,^^110^ (For details on dosage and types, please see Table S5.)

Nutrition-sensitive strategies included cash transfers and food vouchers (*n* = 15),^24^^,^^25^^,^^28^^,^^29^^,^^37^^,^^40^^,^^47^^,^^50^^,^^52^^,^^63^^,^^71^ home visits for early childhood development interventions, including responsive stimulation (*n* = 12),^42^^,^^58^^,^^78^^,^^79^^,^^86^ parenting classes,^70^ maternal mental health and psychosocial support,^31^^,^^33^^,^^36^^,^^38^^,^^42^^,^^111^ water, sanitation and hygiene (WASH) services (such as provision of soap or hygiene kits [*n* = 11],^31^^,^^36^^,^^77^^,^^92^ ensuring safety of drinking water supply,^26^^,^^36^^,^^92^^,^^113^^,^^119^ and sanitation campaigns,^31^^,^^40^^,^^80^^,^^92^^,^^113^^,^^117^) child protection (*n* = 5),^34^^,^^60^^,^^108^^,^^110^^,^^112^ and family planning services (*n* = 4).^79^^,^^88^^,^^91^^,^^102^

Health interventions included child immunization services (*n* = 21),^26^^,^^31^^,^^36^^,^^39^^,^^40^^,^^43^^,^^45^^,^^47^^,^^48^^,^^51^^,^^53^^,^^57^^,^^60^^,^^69^^,^^73^^,^^74^^,^^76^^,^^81^^,^^84^^,^^117^^,^^124^ health promotion by community health workers (CHWs) (*n* = 14),^36^^,^^44^^,^^74^^,^^87^^,^^88^ malaria prevention and treatment (*n* = 5),^61^^,^^73^^,^^91^^,^^102^^,^^119^ HIV services (*n* = 5),^40^^,^^64^^,^^73^^,^^77^^,^^102^ and training of health workers in essential newborn care practices and integrated illness management (*n* = 5).^32^^,^^86^^,^^87^^,^^105^^,^^119^ Four studies assessed mobile health teams (MHTs) providing integrated health and nutrition services in insecure regions.^43^^,^^53^^,^^90^^,^^99^

Behavior change communication (BCC) interventions in 13 studies encompassed health, nutrition, and WASH behaviors.^25^^,^^29^^,^^54^^,^^56^^,^^71^^,^^80^^,^^83^^,^^84^^,^^91^^,^^103^^,^^105^^,^^114^^,^^115^

### Comparator

Integrated programs were compared with control groups wherein participants had access to basic health services provided by the government,^45^^,^^48^^,^^50^^,^^54^^,^^66^^,^^67^^,^^70^ standard nutritional management and IYCF counseling,^28^^,^^46^^,^^47^^,^^55^^,^^58^^,^^79^ and routine humanitarian programs.^25^^,^^27^^,^^37^^,^^80^^,^^84^^,^^86^ Some programs provided donations of essential medication at the end of the project^83^ or cash transfers only.^24^^,^^71^

### Primary Outcomes

#### Coverage and Utilization

Coverage and utilization were most frequently reported for interventions and platforms, as outlined in Table 2.

**Table 2.** Summary of Most Frequently Reported Interventions

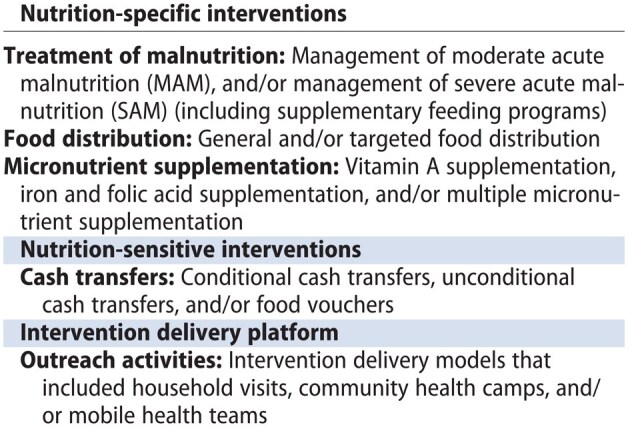

##### Treatment of malnutrition

Prevention and management of malnutrition was featured in 23 peer-reviewed articles^25^^,^^26^^,^^28^^,^^30^^,^^31^^,^^35–37^^,^^40^^,^^41^^,^^43^^,^^47^^,^^52^^,^^55^^,^^57^^,^^60^^,^^62–64^^,^^66^^,^^75^^,^^80^^,^^85^ and 18 gray literature reports.^88–95^^,^^97–100^^,^^103^^,^^107–110^^,^^112^^,^^116^^,^^119^ Twelve articles reported on the coverage of malnutrition treatment.^28–31^^,^^40^^,^^41^^,^^90^^,^^91^^,^^94^^,^^98^^,^^99^^,^^109^ The proportion of districts in Kenya providing routine SAM treatment increased from 53% in 2018 to 60% in 2020.^40^ In Cameroon, coverage of health districts/nutrition service delivery points increased by 16% in 1 year after implementation of an integrated prevention program.^91^

Distribution of fortified foods was reported in 5 studies.^53^^,^^54^^,^^77^^,^^107^^,^^109^ Coverage of fortified cereals did not increase in Nigeria after integrated outreach programs.^53^ Coverage of protein-fortified flour in Kenya was 72.5% for at least 1 bag and 53% for 2 bags in a clinic-based integrated program.^77^ Importantly, 86.4% of pregnant and lactating women received fortified biscuits, and 98.3% of children aged 6–23 months received fortified blend foods at least once at health posts.^107^ Super Cereal Plus was also provided in 2 studies.^24^^,^^91^

Four programs provided lipid-nutrient supplementation (LNS).^26^^,^^29^^,^^52^^,^^55^^,^^122^ An integrated IYCF and LNS program in Democratic Republic of Congo reported that 70.2% of target children had tried LNS and 74.9% of children had consumed all 28 sachets from the last distribution.^55^ Compliance of participants with LNS was 94.1% in the group that pursued an integrated BCC and unconditional cash transfer strategy, compared with 82.7% of participants who received supplements with cash transfer only (*P* < 0.001).^29^ In addition, the group that received BCC reported fewer sharing of LNS sachets with nontarget family members (*P* < .001).^29^

##### Food distribution

General food distributions were reported in 11 programmes.^26^^,^^31^^,^^52^^,^^54^^,^^55^^,^^62^^,^^74^^,^^91^^,^^109^^,^^110^^,^^118^ An integrative emergency nutrition response in Cameroon reached 90% of participants with at least 2 food distributions.^91^ Preventive blanket supplementary feeding was reported by 2 programs.^108^^,^^109^ Targeted food distribution programs were included in 8 studies.^24^^,^^53^^,^^54^^,^^57^^,^^63^^,^^77^^,^^107^

##### Micronutrient supplementation

Sixteen studies reported on the provision of vitamin A supplementation using various integrative strategies (Table S5).^30^^,^^33^^,^^39^^,^^40^^,^^45^^,^^51^^,^^57^^,^^59^^,^^61^^,^^69^^,^^73^^,^^76^^,^^81^^,^^84^^,^^90^^,^^98^ Multiple micronutrients were integrated in 4 studies.^25^^,^^78^^,^^79^^,^^86^^,^^95^ Fewer households (11%) reported noncompliance with multiple micronutrient supplementation when they received integrated services, compared with nutritional counseling only (24%).^86^ Maternal and child iron–folic acid supplementation was reported in 6 studies.^41^^,^^81^^,^^84^^,^^88^^,^^93^^,^^113^ Coverage of pediatric iron folic acid supplementation was 66.5% of participants who received integrative services compared to only 1.4% of those who received standard nutrition programming.^41^

##### Cash transfers

Cash transfers had broad reach and near universal uptake.^24^^,^^25^^,^^28^^,^^29^^,^^37^^,^^47^^,^^50^^,^^52^^,^^63^^,^^71^^,^^82^^,^^95^^,^^118^ However, in certain settings, coverage targets could not be met.^97^ For instance, multipurpose cash distribution in Jabal Mara only reached 11% of its target. Distribution delays were attributed to security reasons and changes in payment modalities.^97^ Food and agricultural vouchers also had reduced, seasonal uptake. Of the total agricultural vouchers made available to women during their pregnancy and postpartum period, 62% were redeemed, most in the wet season.^82^ In Niger, all households received the full amount of the emergency cash transfer, and 98% attended all conditional educational sessions.^37^ Even with soft conditionality, attendance at nutritional educational sessions was high, with 96% of households attending at least 1 training session.^50^ In Somalia, 66% of the caregivers attended all 12 nutritional counseling sessions in the absence of conditionality.^28^ Cash plus food models were reported on in 6 articles.^24^^,^^25^^,^^29^^,^^37^^,^^52^^,^^63^ Notably, a study found that utilization of monthly cash transfers by caregivers to purchase food for the target child was only 20%–40%.^63^ Similarly, a quasi-experimental study in Niger found that the target child was the sole consumer of food supplements in 30%–51% of the households and that sole consumption was higher when families received a food ration compared with a cash transfer.^52^

##### Outreach activities

Fourteen articles reported on health and nutrition services delivered via community-based outreach strategies. Mobile teams,^43^^,^^48^^,^^99^ community health workers (CHWs),^49^^,^^79^^,^^83^^,^^84^^,^^86^^,^^88^^,^^120^^,^^122^^,^^124^ and nutrition support workers^58^ were used for outreach. In India, home visits in the third trimester were higher (33.9%) in the group that received an integrated package compared with controls (17.8%) (*P* < .05).^84^ Similarly, an integrative nutrition and early developmental intervention delivered by CHWs in Pakistan achieved coverage of 77% of households, compared with 75% in standalone programs and 12% in the control group (who received routine services).^86^ Postnatally, 42.6% of women had all 3 home visits by a CHW.^87^ In Afghanistan, community-based nutrition found coverage of home visits by CHW increased by 5%–10%. In comparison, the coverage achieved by growth monitoring promotion sessions increased substantially (90% in certain districts).^89^ Notably, 86.5% of caregivers impacted by the Syrian refugee crisis reported improved access to preventive nutrition services as home visits by CHWs were scaled up.^108^ Integrated mobile health teams in Afghanistan achieved a 5%–10% increase in coverage of health and nutrition services.^99^ Emergency mobile medical teams in South Sudan investigated disease outbreaks and achieved higher reactive measles and cholera vaccination coverage.^43^ Finally, a community-based nutrition package in Cameroon found coverage of nutrition programming improved from 52.3% in 2015 to 59.9% in 2017, as mothers were trained and equipped with a mid-upper arm circumference bracelet.^92^

#### Impact

##### Anthropometric outcomes

Impacts on anthropometric measures were evaluated in 19 articles. Nine articles reported data that could not be meaningfully aggregated.^26^^,^^27^^,^^30^^,^^47^^,^^57^^,^^69^^,^^90^^,^^107^^,^^119^ Four studies described impacts on child growth without providing numerical data on anthropometric measures.^37^^,^^52^^,^^73^^,^^114^ Nonetheless, 6 experimental studies reporting on anthropometric outcomes (wasting, underweight, stunting) were combined in a meta-analysis (Table 3).^24^^,^^25^^,^^28^^,^^29^^,^^70^^,^^80^

**Table 3.** Studies Reporting Anthropometric Outcomes and Included in Meta-Analysis

Reference	Interventions	Location and context	Delivery points	**Participants**
Fenn et al (2017)^25^	UCTs of $14-$28/month, FFVs for 6 months	114 villages in Dadu district, Pakistan, highly vulnerable to climatic shocks	Cash transfers given at central distribution points FFVs distributed to households at village level	Poor or very poor households with at least 1 child aged 6-48 months
Sibson et al (2018)^24^	Education session + screening for malnutrition + targeted supplementary food distribution + 36 pounds/month for 4 months during the “lean” season	39 villages in Tahoua, southwest Niger with sedentary agro-pastoral communities highly reliant on single, unpredictable rainy or “lean” season	Cash and supplementary food given in-hand to female household representative at distribution points within 5 km of the villages	Mothers of children aged 6-59 months
Head et al (2019)^80^	Integrated WASH and nutrition activities implemented in 2011	12 villages in Oromia region, Ethiopia, with low rainfall and frequent droughts	CHWs visited households at least once a month	Mothers of children aged 6-59 months
Ali et al (2022)^28^	Weekly nutritional counseling (NC) + UCT of $40/month for 3 months	6 IDP camps in 2 districts in Banadir, Somalia, with unregulated housing managed by local managers known as “gatekeepers”	NC delivered in health facilities CNVs visited caregivers in camps UCT was provided via mobile phone	Caregivers with mild to moderately malnourished children aged 6-59 months
Fahmida et al (2022)^70^	Parenting classes + food assistance twice weekly for 8 months	Two villages from 2 subdistricts in Indonesia affected by the 2018 Lombok earthquake	Delivered in early child education (ECE) centers by nutritionists from public health centers	Caregivers with children aged 6-49 months
Soofi et al (2022)^29^	UCT of $36 quarterly for + SBCC by LHWs + locally produced MQ-LNS 30 sachets given monthly	200 LHW catchment areas in villages in Rahim Yar Khan district in Punjab, Pakistan	Cash transfers and MQ-LNS given at BISP distribution points. 18 individual household SBCC sessions and 6 in community conducted by LHWs	The poorest households with children at 6 months of age

Abbreviations: BISP, Benazir Income Support Programme; CHW, community health worker; CNV, community nutrition volunteers; FFV, fresh food vouchers; IDP, internally displaced persons; LHW, Lady Health Workers; MQ-LNS, medium-quantity lipid-nutrient supplementation; NC, nutritional counseling; SBCC, social behavior change communication; UCT, unconditional cash transfer; WASH, water, sanitation and hygiene.

###### Wasting (WHZ)

**Figure 3.**
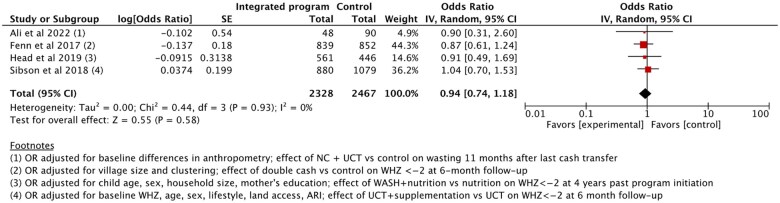
Comparison 1: Outcome 1.1: Prevalence of Wasting (WHZ) in Children Under 5 Years of Age

Seven studies reported on the prevalence of wasting.^24–26^^,^^28^^,^^30^^,^^57^^,^^80^ Three studies were impact evaluations^26^^,^^30^^,^^57^ lacking disaggregated data that could be meaningfully pooled. Thus, excluding these studies, the meta-analysis included 4 trials (*n* = 4795).^24^^,^^25^^,^^28^^,^^80^ There was no significant effect of integrated programs on wasting prevalence compared with stand-alone programs (Figure 3).^24^^,^^25^^,^^28^^,^^80^

###### Underweight (WAZ)

**Figure 4.**
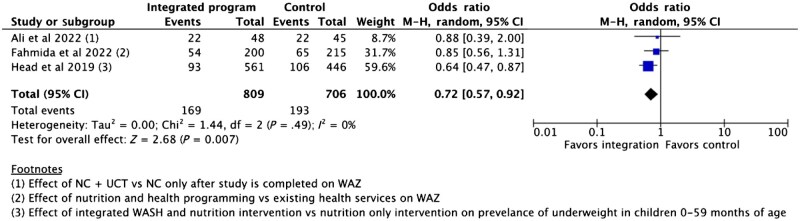
Comparison 1: Outcome 1.2: Prevalence of Underweight (WAZ) in Children Under 5 Years of Age

Five articles^28^^,^^47^^,^^57^^,^^70^^,^^80^ reported on underweight in children under 5 years of age. Two articles reported changes in WAZ scores with no data on incidence.^47^^,^^57^ Thus, excluding these 2 articles, 3 experimental studies^28^^,^^70^^,^^80^ (*n* = 1515) were meta-analyzed, and the results suggested a 28% reduction in the odds of underweight in children under 5 years of age who received an integrated programmatic service (OR [Random]: 0.72, 95% CI: [0.57, 0.92]) (Figure 4).^28^^,^^70^^,^^80^

##### Stunting (HAZ)

**Figure 5.**
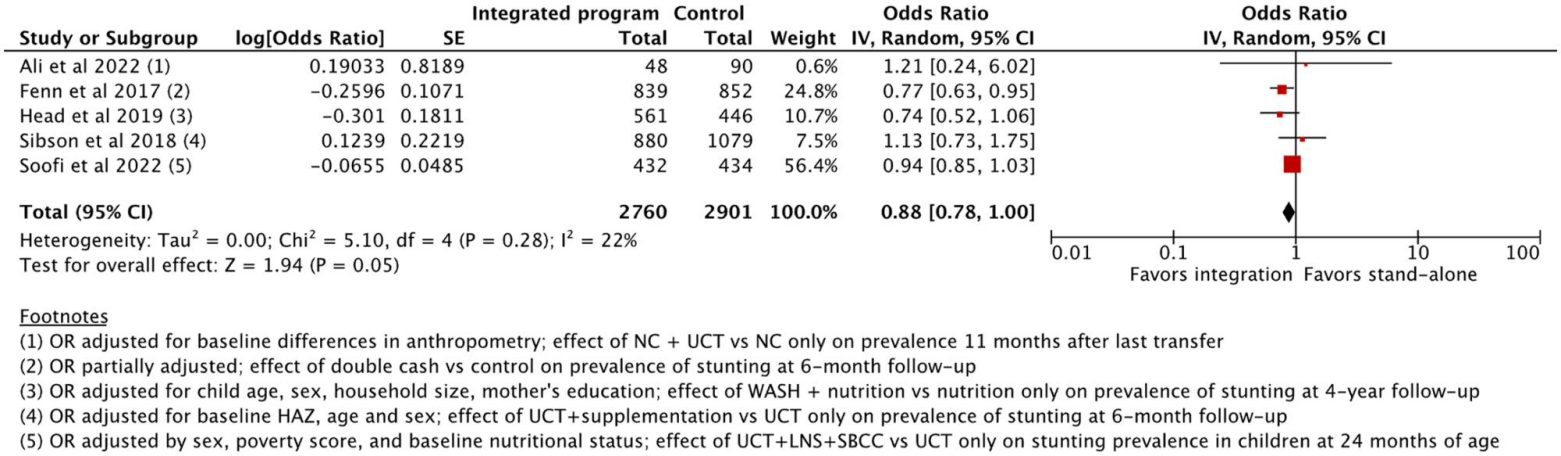
Comparison 1: Outcome 1.3: Prevalence of Stunting (HAZ) in Children Under 5 Years of Age

Six articles^24–26^^,^^28^^,^^29^^,^^80^ reported on stunting in children. An impact evaluation^26^ reported disaggregated data on prevalence (6–23 months and 24–59 months) and, therefore, lacked data to aggregate meaningfully. Five studies^24^^,^^25^^,^^28^^,^^29^^,^^80^ (*n* = 5661) were meta-analyzed, and the pooled OR indicated a 12% decreased odds of stunting in children (OR [Random]: 0.88, 95% CI: [0.78, 1.00]), which bordered on statistical significance (*P* = .05). Heterogeneity was estimated to be 22% (Figure 5).^24^^,^^25^^,^^28^^,^^29^^,^^80^

##### Food security

Ten peer-reviewed articles and two gray literature documents reported on food security, with some using food security scales such as the Household Food Insecurity Access Scale score,^67^ the Arab Family Food Security Scale score,^65^ and the Food Consumption Score,^47^ while most articles used dietary diversity indicators, including Minimum Dietary Diversity, to assess complementary feeding in children 6–59 months.^28^^,^^37^^,^^49^^,^^55^^,^^71^^,^^114^ Additionally, individual dietary diversity scores^28^^,^^47^^,^^70^^,^^71^ and household dietary diversity scores^28^^,^^114^ were used to measure food security.

Although most studies that measured food security did not find a significant improvement in food security, a program evaluation in Lebanon found that moderately food insecure households targeted by community-based kitchen and school-based nutrition programs became food secure, as measured by an endline 7-point Arab Family Food Security Scale score.^65^ In Niger, emergency conditional cash transfers, accompanied by education and cooking demonstration sessions, revealed a significant improvement in minimum dietary diversity in children under 5 years (*P* < .001).^37^ A cluster RCT evaluating a 4-year health and nutrition program in Burundi found that integrated food rations, health service strengthening, and a nutrition and hygiene BCC strategy reduced the proportion of food-insecure households and improved dietary diversity significantly (*P <* .05).^67^

##### IYCF

Thirteen peer-reviewed articles and 2 gray literature documents reported on early initiation of breastfeeding,^32^^,^^50^^,^^55^^,^^84^^,^^87^ exclusive breastfeeding,^33^^,^^49^^,^^50^^,^^71^^,^^73^^,^^120^ and reduced difficulties with breastfeeding practices.^42^ While positive results for early initiation of breastfeeding were frequent,^55^^,^^84^ a matched impact on exclusive breastfeeding was not seen. A cash transfer and BCC initiative in Yemen significantly increased maternal knowledge of exclusive breastfeeding (*P* < .01) without a significant rise in exclusive breastfeeding probability.^50^ A 10-year multi-country nutrition and health program with extensive community-based BCC on IYCF led to significant increases in exclusive breastfeeding (*P* < .05) in Ethiopia.^73^ Recall of breastfeeding and complementary feeding advice by mothers was significantly higher in the group that received contact with CHWs.^84^ A statistically significant increase in rates of exclusive breastfeeding was reported by an evaluation of the postnatal home-visiting program between 2010 and 2014 in the Gaza Strip, State of Palestine.^111^

##### WASH

While WASH sectoral activities and messages were integrated in 12 articles,^25^^,^^29^^,^^31^^,^^35^^,^^37^^,^^49^^,^^50^^,^^55^^,^^73^^,^^78^^,^^80^^,^^108^ 5 articles reported impacts on hygiene behavior and nutritional status of participants. Provision of WASH and nutrition services with reproductive healthcare in Kenya found significant improvements in hand-hygiene practices when caring for the newborn (*P* < .001).^77^ Similarly, an integrated IYCF-small quantity (SQ)-LNS program found improvements in handwashing behavior (*P* < .001) but no impact on dietary diversity of children in a food-insecure area in Democratic Republic of Congo.^55^ Monthly home visits to improve IYCF and WASH behaviors in a refugee camp in Thailand found safer disposal of stool (*P* = .04) and improved dietary diversity (*P* < .001) in a relatively food-secure population.^49^ An integrated WASH and nutrition intervention reported fewer households practicing open defecation, and the mean HAZ to be significantly higher, in these households, compared with the control group (*P* < .05).^80^ The Integrated Emergency Health, Nutrition, Protection and WASH project by World Vision found that 69.1% of participants considered their health, protection, and WASH needs met, at endline, compared with 14.5% before the start of the project. Additionally, 85.6% of caregivers reported access to emergency preventative nutrition services had improved.^108^

##### Mental health

Mental health services such as psychosocial support and first aid were provided in 11 programs.^31^^,^^36^^,^^38^^,^^42^^,^^44^^,^^56^^,^^58^^,^^65^^,^^70^^,^^99^^,^^100^ Five articles reported on maternal and child mental health.^42^^,^^44^^,^^58^^,^^65^^,^^70^ One article reported on cognitive development and found 10-point higher social emotional scores in children older than 2 years of age (*P* < .010) in response to a community-based intervention that adopted integrated nutrition and early childhood development strategies.^70^ However, the same article reported a nonsignificant improvement in maternal depression. A brief intervention integrating psychosocial activities with established nutritional education and emergency feeding programs in northern Uganda found improvements in involvement (*P* < .001) and mood (*P* = .003) of displaced mothers of malnourished children.^58^ Following the creation of baby-friendly spaces in Cameroon, pregnant and lactating women reported reduced psychological suffering, increased perceived social support, improvements in mother–baby interactions, and reduced difficulties in breastfeeding (*P* < .001).^42^ Integrated health and nutrition summer camps for children and adolescents in Lebanon were associated with improved nutrition and life-skills knowledge scores (*P* < .001).^44^ However, a community kitchen project in Lebanon was not associated with significant improvements in mental health inventory scores of Palestinian refugee women, despite reduced food insecurity scores (*P* < .001).^65^

### Secondary Outcomes

Barriers and facilitators were categorized based on 4 domains: Operations and logistics, human resources, communication, and community-related factors. The cost-effectiveness data available were also examined.

#### Barriers and Facilitators

##### Partnerships and operations

Aspects related to program operations and logistics were the most frequently cited influencing factors for integrated programs. Strong operating plans^39^ and efficient logistics systems^31^ were highlighted as critical for the success of integrated operations. More specifically, articles emphasized the need for a robust coordinating platform with close interaction between all implementing partners.^31^^,^^91^^,^^106^ Concurrently running programs with common goals but minimal coordination hindered implementation and impact.^81^^,^^121^^,^^127^ Several articles highlighted that strong partnerships were a facilitator for integrated programs, especially engagement with local actors, including civil society organizations with an existing presence and trust in the target communities.^40^^,^^61^^,^^72^^,^^90^^,^^91^ The articles also described struggles of developing appropriate partnerships that aligned with health and nutrition project needs.^96^ Reasons for the reluctance of local partners to engage ranged from apprehension of new partnerships, fear of time and resource commitment,^46^ and the lack of buy-in on the interventions being integrated.^56^ A few articles^38^^,^^56^ reported that integration of psychosocial support for mothers with nutrition-specific interventions was considered non-essential by partners. A case study on digital health and nutrition solutions implemented in Burkina Faso recognized stakeholder analysis and the consequent stakeholder engagement strategy as key to developing strong and adaptive partnerships.^116^

Of all the partnerships highlighted, engagement with government bodies and institutions in conflict areas was recognized as pivotal. Government support allowed for smooth implementation rollout, increased accountability for stakeholders, and stronger ownership of the program, with more chances of integration into existing health services.^29^^,^^40^^,^^61^ Engaging and building capacity of the local government of target areas was identified as a strong facilitator for program sustainability.^29^^,^^40^^,^^61^^,^^90^^,^^91^ Conversely, a lack of government support impeded smooth implementation, especially in the context of humanitarian crises.^96^^,^^110^^,^^127^

Coordinated multisectoral integration^30^ was imperative for integrated strategies to work well, and gaps in coordination^81^ or missed opportunities to leverage multisectoral synergies often led to ineffective implementation.^103^^,^^110^ Studies identified that disjointed efforts by different sectors at various government and health system levels—from the ministries and organizational heads to the frontline workers—led to reduced program impact.^81^ A lack of interest and motivation by the different multisectoral partners, such as erratic engagement and participation, was noted as a barrier to integrative efforts.^81^ Lack of ownership and fragmented efforts in the humanitarian sector were highlighted in 2 reports.^56^^,^^90^ Administrative and bureaucratic barriers were reported to impact preparedness and delay emergency health and nutrition responses.^127^

Two articles acknowledged logistics and supply chains as fundamental elements.^31^^,^^91^ An inadequate budget for all intervention components, absence of operational banks in active conflict settings, and delays in the flow of funds because of multisectoral administrative barriers led to delays in supply delivery and operational constraints.^31^^,^^33^^,^^83^^,^^89^^,^^100^^,^^102^^,^^105^^,^^109^^,^^113^ Integrated strategies with multiple interventions and multiple needs were an added strain on these systems, requiring funds, coordination, and a streamlined logistics system for efficient implementation.^106^ A few articles documented the hurdle of remote management and virtual communication in conflict settings with partners based internationally, leading to delays in decision-making and implementation.^90^^,^^96^ Communication challenges were also noted within the country setting, with damaged telecommunication systems, poor mobile network coverage, and electricity supply issues that limited coordination, data reporting, and referral systems.^43^^,^^64^^,^^90^^,^^116^^,^^127^ An integrated program in Cameroon noted that logistical and warehouse management training provided to frontline health workers aided efficiency and smooth implementation.^91^ Several articles noted that a baseline assessment focusing on population health and nutritional status, existing health systems, and the availability of resources, contributed to successful planning and implementation.^24^^,^^36^^,^^94^^,^^100^

#### Human Resources

Health worker and team engagement-related factors were highlighted in several articles. Community health workers were recognized as an asset for integrated programs, given their skills, close connections with the community, and knowledge of local customs and language.^38^^,^^55^ A program evaluation in Ethiopia noted the benefits of using the Health Extension Workers’ platform to operationalize a health and IYCF program, owing to their knowledge and existing integration in the community.^102^ Engagement of female health workers in conservative contexts enhanced community receptivity.^35^ Articles highlighted the importance of using context-specific, trained, multidisciplinary outreach teams for integrated strategies in conflict settings, such as deploying doctors, nurses, CHWs, nutritionists, and health promotion experts, to increase impact.^43^ Finding personnel with varied expertise and skills is a challenge in most emergency contexts, and a shortage of trained personnel was a barrier reported by several articles.^43^A lack of knowledge of maternal and newborn health, poor communication skills, and a dearth of workers trained in nutrition were noted as major implementation obstacles.^32^^,^^38^^,^^64^ In some contexts, medicines and equipment went unused, as personnel were neither knowledgeable about nor trained to use them.^33^ An integrated health and IYCF outreach intervention in Haiti reported a low referral rate that was attributed to the inability of health workers to identify children with complicated severe acute malnutrition.^33^ Conversely, frustration of health workers was also noted in several articles, with concerns of increased responsibility and high workload in integrated strategies.^39^^,^^41^^,^^45^^,^^61^^,^^64^^,^^74^^,^^89^^,^^102^^,^^103^ Low wages and a lack of incentives led to worker discontent.^61^^,^^64^^,^^102^ Many articles reported high staff turnover and attrition, leading to disruption of service delivery, impacting intervention quality and sustainability.^75^^,^^96^^,^^97^^,^^102^ Failure to adhere to guidelines and faulty practices were also noted in the context of high workload and insufficient training.

#### Community Engagement

Several articles emphasized the importance of community leader engagement, community-level ownership, and support at every stage of implementation of an integrated delivery program.^40^^,^^72^^,^^96^^,^^115^ The participatory decision-making approach and ongoing community feedback mechanisms were recognized as enabling factors for sustainable implementation.^40^^,^^72^^,^^96^^,^^115^ Involvement of community members in project design, selection of target sites, delivery of interventions, and data collection was linked to positive perceptions and sustainability of integrated health and nutrition programs. Three articles^46^^,^^70^^,^^78^ described leveraging community resources, such as nominating community women to serve as frontline workers, or using school infrastructure to run health camps. Poor community mobilization was documented as a barrier to project uptake and community acceptance.^30^

Navigating cultural norms and traditional beliefs is important, even in the crisis context. Many articles documented community resistance to changing IYCF practices, such as giving colostrum and regarding immunization.^50^^,^^59^^,^^94^^,^^105^ In many contexts, endorsement from religious leaders was key for community acceptance of interventions, particularly immunization.^39^ In line with local culture, engagement of key household decision-makers (such as men, grandmothers, and mothers-in-law) was fundamental for behavior change and program engagement.^40^^,^^105^

#### Communication Strategies

Given the challenges of changing health and nutrition practices, the need for a strong BCC strategy was highlighted, and the success of programs was attributed to effective BCC.^29^^,^^55^^,^^66^^,^^67^ A program report on integrated maternal and infant nutrition interventions in a refugee camp in Kenya reported that involvement of stakeholders and community members in the development of the communication strategy enhanced uptake and adoption.^105^ Several articles highlighted the importance of context-specific and culturally appropriate messaging in local languages.^33^^,^^49^^,^^72^^,^^107^^,^^120^ Conversely, messages that were not culturally sensitive were often resisted or ignored by communities.^44^ It was noted that, in the context of integrated strategies, multiple messages were being communicated simultaneously, which often diluted the impact.^50^^,^^72^^,^^76^ The materials and messages covered multifactorial behavior determinants, thus complicating the BCC strategy further.^83^ In conflict settings, the interventions were short term, while most BCC strategies required long-term exposure for impact and conversion into optimum behaviors.^114^

#### Cost-Effectiveness

The cost-effectiveness of integrated programs was measured by 3 studies. A RCT conducted in Pakistan,^27^ examining the cost-effectiveness of an integrated program of cash-based interventions and BCC strategies, found that integration was cost-effective when assessed as cost per case of stunting and disability-adjusted life-year averted. Another study^79^ found that integrating responsive stimulation and nutrition interventions was more cost-effective than implementing them individually, given the savings on human resources. However, Levin et al^82^ found that implementing an agriculture, health, and nutrition program led to a substantial cost per beneficiary. The researchers noted that the biggest cost category was multisectoral integrative measures, including coordination and monitoring activities, and administration.

## DISCUSSION

### Summary of Key Results

This systematic review and meta-analysis synthesized the evidence on nutrition-specific strategies integrated with health and/or nutrition-sensitive interventions in conflict-affected settings of LMICs. The review included 61 peer-reviewed articles and 32 reports from the gray literature that focused on integrated programs in 25 countries, across 4 continents. The present review showed that, while coverage and uptake of integrated nutrition programs was substantial, particularly when integrated with existing health systems and government engagement, the impact of the interventions, especially on nutritional outcomes, was limited. The meta-analysis revealed no significant difference in wasting or stunting outcomes in children under 5 years, though a 28% statistically significant reduction in the risk of underweight was seen in those children who received an integrated strategy compared with those who did not. The 12% reduction in stunting risk in children who received an integrated strategy approached statistical significance and warrants further exploration in similar contexts. Facilitators of implementation were coordinated efforts by multisectoral partners, strong engagement with the government, community participation, and integration with existing health systems. Specific to nutrition, robust BCC components, health workers with strong counselling skills, and context-appropriate messaging were factors most frequently associated with impactful programs. The most cited barriers to implementation were insecure and unpredictable contexts, limited access, logistical challenges, difficulty in maintaining a skilled workforce, and community resistance. Certain factors, such as unpredictable and insecure contexts, logistical constraints, weakened health systems, and population movement, impeded implementation and are largely inevitable in conflict-affected settings. Conversely, modifiable barriers, such as inadequate planning and coordination, insufficient training, weak community engagement, and ineffective communication strategies can be prioritized and addressed in future programming.

### Comparison with Previous Studies

While the extent of nutrition integration as it relates to the World Health Organization building blocks of health systems has been examined in the literature,^128^ the present review aimed to examine nutrition integration in terms of uptake, impact, and practical considerations of implementation. There is limited evidence on the uptake and feasibility of integrated health and nutrition programs in conflict settings, especially considering barriers, facilitators, and cost-effectiveness.^129^ Shah and colleauges collated evidence on nutrition-specific interventions targeting women, children, and adolescents in conflict settings of LMICs and concluded that general food distribution, micronutrient supplementation, and nutritional assessment were the most reported interventions, largely delivered in refugee camps and community-focused platforms.^13^ The review also recognized integrating nutrition interventions with other sectoral strategies as a delivery facilitator. Abdullahi et al focused on research exploring the integration of under-5-focused nutrition-specific interventions with nutrition-sensitive interventions in fragile contexts and reported that integrated community case management and Integrated Management of Childhood Illness were the most common integration platforms.^129^ Similar to our findings, they found immunization and cash transfers being commonly integrated. The authors also indicated the need for additional studies looking at feasibility of integration, local capacities, and relevance.^129^ Bridge and Lin^130^ systematically reviewed the evidence on CHW strategies addressing under-5 undernutrition in conflict-affected settings and examined effectiveness and acceptability.

### Features of Integrated Programs

The integrative strategies included in this review were heterogeneous.^131^ Most articles reported on co-delivery of multi-sectoral services for a specific population groups, while others reported on single delivery points at which a selection of services are offered. The review purposely included different integrative strategies to ensure an in-depth assessment of varied forms of integration, their impact, and their feasibility, to inform future policy, planning, and practice. The review also distinguished programs based on nutrition-specific, nutrition-sensitive, and health components. Integrated strategies with exclusively nutrition-specific and nutrition-sensitive interventions were the least prevalent, most likely due to the expected prioritization of health interventions in such contexts. Examination of the various strategies showed that a positive impact was seen most frequently with integration of all 3 components. However, given the heterogeneity in study methods and the unavailability of disaggregated data, subgroup analyses were not possible by program components.

Several articles emphasized the benefits, including cost-sharing and enhanced accountability, of integration of strategies with other sectors rather than stand-alone programs.^30^^,^^38^^,^^40^^,^^107^ Community receptivity to interventions, such as polio vaccination, vitamin A supplementation, and family planning services, were increased when packaged with other health and nutrition interventions.^48^^,^^61^^,^^102^ The success of integrated programs was commonly associated with strategies in which interventions were combined with an existing health system or healthcare structures, such as vitamin A supplementation integrated with the existing seasonal malaria chemoprevention platform or the provision of Community Management of Acute Malnutrition at existing health centers.^29^^,^^36^^,^^61^^,^^100^ Challenges with data collection were noted for integrated strategies, given the added interventions and additional variables, which increases complexity and workload.^32^^,^^33^^,^^122^

Though under the umbrella of humanitarian crises, conflict-affected settings are a unique context, with uncertainty and ongoing distress.^38^^,^^40^^,^^53^ Health systems are weakened, and infrastructure damaged or lacking in essential resources and supplies.^41^ The articles also documented challenges with constantly changing populations, with displacement of large numbers of people or high influx of refugees.^28^^,^^33^^,^^46^^,^^90^ Unexpectedly high numbers put a strain on the integrated health and nutrition campaigns.^48^ Additionally, most settings are impacted by not just conflict but also climate change, further adding to population health and implementation risks. Predictably, some programs found that intervention impact was reduced and health and nutrition situations worsened when faced with a climatic event within a conflict setting.^38^^,^^40^^,^^69^

Integrated programs in the conflict context are often donor-dependent, making their sustainability uncertain and often resulting in community apprehension.^39^^,^^59^^,^^63^^,^^88^ Integration with government health systems and local community initiatives can enhance the lifespan of these programs. By example, before phasing out a pilot mobile health service program in a high-risk area of Afghanistan, implementing organizations worked on building the capacity of the local health centers to ensure the community continued to receive health and nutrition services and were not left unsupported.^99^

### Nutrition Interventions in Integrated Programs

The nutrition interventions most included in integrated strategies were acute malnutrition management, supplementary foods, and IYCF counseling. Notably, key components that were reported to enhance the impact of nutrition interventions within integrated strategies were combining supplementary food rations with cash transfers^47^^,^^52^ and/or with household food rations,^66^ to support household food security and ensure supplementary food rations reach the child in need. A few articles also highlighted the disproportionate focus on acute malnutrition.^57^ While breastfeeding promotion was common, tailored IYCF counseling support for mothers and psychosocial assistance were rare.^33^^,^^34^ Also noted was the distribution of infant formula in humanitarian settings, harming breastfeeding efforts.^34^ Several articles noted the sidelining of nutrition interventions in integrated strategies and the lack of inclusion of nutrition indicators in monitoring frameworks.^89^

### Strengths and Limitations of the Review

This review captured a breadth of published and gray literature examining a range of integrative strategies for nutrition and health interventions, using systematic methods and an in-depth qualitative analysis. Given the high number of conflict settings globally, there may have been integrated nutrition and health programs that were not captured in the literature, which thus would not have been evaluated. We only included English language articles, which may have excluded evidence on integrated programs from Francophone conflict settings. We also recognize that some integrated programs may not have been included as they did not align with our protocol definition.

### Implications for Future Research, Policy, and Practice

An interplay of factors impact the nutritional status of women and children. As complexity increases in conflict settings, it is recommended that future programs conduct baseline assessments, if possible, to understand the context, including cultural and religious norms and community beliefs, to develop tailored strategies. Importantly, nutrition-sensitive strategies, such as WASH, should be delivered with nutrition-specific interventions for synergistic impacts. Active engagement with government and local partners is imperative. The institutionalization of integrated programs into national health systems and enhancing government and community ownership could enhance efficiency and sustainability, given challenging and dynamic environments. Future research should focus on cost-effectiveness assessment of integrated strategies and on evaluating nutritional impact with standardized measures. Considering their potential efficiency and cost-effectiveness, integrated nutrition and health interventions are promising strategies in conflict settings, as long as they are deployed with optimal planning and close government and multisectoral coordination.

## CONCLUSION

Integrated service provision and quality data have both been identified as essential components of developing high-quality health systems for nutrition.^132^ Conflict settings require efficient and comprehensive responses through the integration of nutrition interventions into existing health interventions as a “minimum package response” to improve efficiency and sustainability, and, ultimately, universal health and nutrition care for all.^128^^,^^130^

## Supplementary Material

nuaf031_Supplementary_Data

## Data Availability

Data are available in the public domain. Data extracted from publications retrieved from the indexed and gray literature are available from the corresponding author upon reasonable request. Further inquiries can be directed to the corresponding author.
