# Supplementary material for: Evidence on Strategies for Integrating Nutrition Interventions with Health and Immunization Systems in Conflict-affected Areas of Low- and Lower-middle-income Settings—A Systematic Review
Source: Nutr Rev. 2025 Apr 12;83(8):1475–93. doi: 10.1093/nutrit/nuaf031 (PMC12241850; doi:10.1093/nutrit/nuaf031)
Supplement: nuaf031_Supplementary_Data [file nuaf031_supplementary_data.zip › Khan_Supplementary File_revised submission Feb 2025.docx]

**Supplementary File**

[Supplementary Table 1. Search strategy (Medline)](#A1)

[Supplementary Table 2. List of LMIC countries affected by conflict](#T2)

[Supplementary Table 3. List of integrated strategies by type of integration](#T3)

[Supplementary Table 4. Characteristics of Included Studies](#T4)

[Supplementary Table 5. Description of nutritional supplementation strategies](#T4)

[Supplementary Table 6. Coverage rates for micronutrient supplementation, immunization under different integration strategies](#T5)

[Supplementary Table 7. PRISMA Checklist](#T6)

[Supplementary Figure 1. Included studies by year of publication](#f1)

[Supplementary Figure 2. Risk of bias (RoB-2) summary: review authors' judgements about each risk of bias item for each included study](#f2)

[Supplementary Figure 3. Risk of bias for non-randomized studies (ROBINS-1) summary: review authors' judgements about each risk of bias item for each included quasi-experimental study](#f3)

[Supplementary Figure 4. Risk of bias (RoB-2) summary: review authors' judgements about each risk of bias item for each included study](#f4)

[Supplementary Figure 5. Risk of bias for program evaluations, cohort and cross-sectional studies (NIH tool) summary: review authors' judgements about each risk of bias item for each included observational cohort and cross-sectional study](#f5)

**[Supplementary Table 1. Search strategy (Medline)](#A1)**

| # | **SEARCH SET 1 – CONFLICT ENVIRONMENT TERMS** | **RECORDS** |
| --- | --- | --- |
| 1. | exp disasters/ |  |
| 2. | exp natural disasters/ |  |
| 3. | Exp tsunami/ |  |
| 4. | Exp volcanic eruptions/ |  |
| 5. | exp "Warfare and Armed Conflicts"/ or exp Nuclear Warfare/ or exp Chemical Warfare/ or exp Psychological Warfare/ or exp Warfare/ or exp Biological Warfare/ |  |
| 6. | exp Afghan campaign 2001/ |  |
| 7. | exp gulf war/ |  |
| 8. | exp Iraq war |  |
| 9. | ((disaster* catastrophe* or cris* or emergenc*) adj5 (environ* or human or man$made or natur* or weather)).ti,ab,sh,kf |  |
| 10. | (mass casualt* or mass fatalit* or disaster victim*).ti,ab,sh,kf |  |
| 11. | ("afghan campaign" or armed conflict* or "gulf war" or "iraq war" or "war time" or "wartime"). ti,ab,sh,kf |  |
| 12. | ((armed or zone or political or civil) adj3 (conflict* or attack* or war* or "no fly")). ti,ab,sh,kf |  |
| 13. | (militant group* or militant organi$ation*).ti,ab,sh,kf |  |
| 14. | (biological terrorism or bioterrorism or biowarfare or chemical terrorism or ethnic cleansing* or gas poisoning or genocide or holocaust* or nuclear terrorism or war exposure*).ti,ab,sh,kf |  |
| 15. | (avalanche* or cyclone* or drought* or earthquake* or famine* or flood* or hurricane* or land$slide* or mud$slide* or storm* or tornado* tsunami* or typhoon* or volcanic ash or volcanic eruption* or volcanic gas*).ti,ab,sh,kf |  |
| 16. | (fragile state or fragile setting).ti,ab,sh,kf |  |
|  | **SEARCH SET 2 – CONFLICT RESPONSE TERMS** | **RECORDS** |
| 1. | exp disaster medicine/ |  |
| 2 | exp emergency medical services/ |  |
| 3. | exp disease outbreaks/ |  |
| 4. | ((rescue or relief or aid) adj2 (plan* or activity*or agenc*)). ti,ab,sh,kf |  |
| 5. | (humanitarian assistance* or "aid plan" or "aid work" or "relief plan" or "relief work" or "rescue plan" or "rescue work"). ti,ab,sh,kf |  |
| 6. | (humanitarian adj2 (aid or response or relief or cris* or emergenc* or disaster*)).ti,ab,sh,kf |  |
|  | **SEARCH SET 3 – CONFLICT PEOPLE TERMS** | **RECORDS** |
| 1. | Exp refugee/ |  |
| 2. | (refugee* or evacuee* or disaster victim*).ti,ab,sh,kf |  |
| 3. | (displaced child* or displaced famil* or displaced individual* or "displaced internally" or "displaced men" or "displaced people*" or "displaced person*" or "displaced population*" or "displaced women" or "forced displacement*" or "internal* displace*" or "population displace*").ti,ab,sh,kf |  |
| 4. | (((camp*) and displac*) or "protected village*").ti,ab,sh,kf |  |
|  | **SEARCH SET 4 – GENERAL POPULATION TERMS** | **RECORDS** |
| 1. | Exp Infant/ |  |
| 2. | Exp Child/ |  |
| 3. | Exp Adolescent/ |  |
| 4. | Exp Young Adult/ |  |
| 5. | Exp Pregnant Women/ |  |
| 6. | Exp Pregnancy/ |  |
| 7. | exp Delivery, Obstetric/ |  |
| 8. | Exp Pregnancy Complications/ |  |
| 9. | (infan* or newborn* or "new born*" or neonat* or baby* or babies or toddler* or boy or boys or boyfriend or boyhood or girl* or kid* or child* or pediatric* or paediatric* or prematur* or preterm*).ti,ab,sh,kf |  |
| 10. | (adolescen* or teen* or youth* or young adult*).ti,ab,sh,kf |  |
| 11. | (expectant mother* or gravid* or pregnan*).ti,ab,sh,kf |  |
| 12. | ("mother to be" or "mothers to be"). ti,ab,sh,kf |  |
| 13. | (prenatal or "pre natal").ti,ab,sh,kf |  |
| 14. | (perinatal or "peri natal").ti,ab,sh,kf |  |
| 15. | ((trimester*) adj3 (first or second or mid or third or final or "1st" or "2nd" or "3rd")).ti,ab,sh,kf |  |
| 16. | (midtrimester* or early placental phase*).ti,ab,sh,kf |  |
| 17. | ((labor or labour) adj5 (birth* or breech or childbirth* or complicat* or difficult or early or easy or induce* or induction or late or obstetric* or onset or pregnan* or present*)).ti,ab,sh,kf |  |
| 18. | parturients.ti,ab,sh,kf |  |
| 19. | (birth* or childbirth* or parturition*).ti,ab,sh,kf |  |
| 20. | (abdominal deliver* or c-section* or caesarean* or cesarean* or "postcesarean section" or "postcaesarean section").ti,ab,sh,kf |  |
| 21. | (abortion* or embryotomy* or "postconception fertility control").ti,ab,sh,kf |  |
| 22. | ((pregnanc*) adj3 (terminat* or complicat*).ti,ab,sh,kf |  |
|  | **SEARCH SET 5 – NUTRITION TERMS** | **RECORDS** |
| 1. | Exp Anthropometry/ |  |
| 2. | Exp Nutritional Status/ |  |
| 3. | Exp Infant Nutritional Physiological Phenomena/ |  |
| 4. | Exp Child Nutritional Physiological Phenomena/ |  |
| 5. | Exp Adolescent Nutritional Physiological Phenomena/ |  |
| 6. | Exp Malnutrition/ |  |
| 7. | Exp Hunger/ |  |
| 8. | Exp Food Assistance/ |  |
| 9. | Exp Food, Fortified/ |  |
| 10. | Exp Milk, human/ |  |
| 11. | Exp Infant Formula/ |  |
| 12. | Exp Breast feeding/ |  |
| 13. | Exp Breast Milk expression/ |  |
| 14. | Exp Dietary Supplements/ |  |
| 15. | Exp Milk Banks/ |  |
| 16. | Exp Micronutrients/ |  |
| 17. | Exp Goitre |  |
| 18. | Exp Nutrition Assessment/ |  |
| 19. | Exp Nutrition Surveys/ |  |
| 20. | ((arm or arms or midarm or midarms) adj3 (anthropometr* or circumference)).ti,ab,sh,kf |  |
| 21. | Muac.ti,ab,sh,kf |  |
| 22. | ("height to weight ratio" or "height weight ratio" or "height for age" or "weight for age" or "weight for height" or "weight for length" or "weight to height ratio" or "haz" or "waz" or "whm" or "whz" or stunt* or under$weight).ti,ab,sh,kf |  |
| 23. | ("adolescent nutrition physiology" or "adolescent nutritional physiological phenomena" or "adolescent nutritional physiological phenomenon" or "adolescent nutritional physiology" or "child nutrition physiology" or "child nutritional physiological phenomena" or "child nutritional physiology" or "child nutritional physiology phenomena" or "child nutritional physiology phenomenon" or "infant nutrition physiology" or "infant nutritional physiological phenomena" or "infant nutritional physiological phenomenon").ti,ab,sh,kf |  |
| 24. | (breast$fe* or breast$milk or breast pumping* or artificial milk or baby$formula* or "formula feeding" or formula* milk or infant formula* or "milk formula" or breast$milk substitute* or similac or synthetic milk).ti,ab,sh,kf |  |
| 25. | (milk$bank*).ti,ab,sh,kf |  |
| 26. | (feeding centre* or feeding center*).ti,ab,sh,kf |  |
| 27. | (diet* modification* or diet* therap* or diet* treatment* or nutrition therapy).ti,ab,sh,kf |  |
| 28. | (hunger or famine or hungry).ti,ab,sh,kf |  |
| 29. | iodine deficienc*.ti,ab,sh,kf |  |
| 30. | (goitre* or goiter*).ti,ab,sh,kf |  |
| 31. | (amino$acid starvation or kwashiorkor* or kwasiorkor or protein deprivation or protein malnutrition or protein starvation).ti,ab,sh,kf |  |
| 32. | (severe acute or moderate acute or deficient nutrition or malnourish* or malnutrition or marasmus or nutritional deficienc*or under$feeding or under$nourishment or under$nutrition).ti,ab,sh,kf |  |
| 33. | ((micronutrient* or trace element* or mineral* or vitamin*) adj5 (deficienc* or "defective diet" or "deficient diet" or "diet insufficienc*" or "dietary insufficienc*" or "nutritional deficit*")).ti,ab,sh,kf |  |
| 34. | ((micronutrient* or trace element* or mineral* or vitamin*) adj5 (diet* or nutrition*or supplement*)).ti,ab,sh,kf |  |
| 35. | (micronutrient powder* or mnp).ti,ab,sh,kf |  |
| 36. | sprinkles.ti,ab,sh,kf |  |
| 37. | ("italian leprosy" or "lombardy leprosy" or maidism or "niacin deficiency" or pellagra* or "pellagrous skin").ti,ab,sh,kf |  |
| 38. | ("dietary assessment*" or "dietary evaluation*" or "mininutrition* assessment*" or "nutrition* assessment*" or "nutrition* index" or "nutrition* indexes" or "nutrition* indices" or "nutrition* evaluation").ti,ab,sh,kf |  |
| 39. | nutrition* surveillance.ti,ab,sh,kf |  |
| 40. | ("nutrition* survey*" or nhanes or "national health and nutrition examination survey" or "health survey" or "smart survey").ti,ab,sh,kf |  |
| 41. | ("nutrition* status" or "nutrition* state").ti,ab,sh,kf |  |
| 42. | ("outpatient therapeutic" adj2 (care or program*)).ti,ab,sh,kf |  |
| 43. | selective feeding.ti,ab,sh,kf |  |
| 44. | stabili$ation centre* or stabili$ation center*.ti,ab,sh,kf |  |
| 45. | starvation*.ti,ab,sh,kf |  |
| 46. | ("supplementary feeding*" or "complementary feeding*" or "complementary food" or "supplementary feeding*").ti,ab,sh,kf |  |
| 47. | therapeutic feeding*.ti,ab,sh,kf |  |
| 48. | therapeutic food*.ti,ab,sh,kf |  |
| 49. | ("thiamine deficienc*" or "aneurin deficienc*" or "avitaminosis b1" or "thiamin deficienc*" or "vitamin b 1 deficienc*" or "vitamin b1 deficienc*").ti,ab,sh,kf |  |
| 50. | (beriberi or "beri beri").ti,ab,sh,kf |  |
| 51. | (iron deficien* or anemia or anaemia).ti,ab,sh,kf |  |
| 52. | ("ascorbic acid deficienc*" or "vitamin c deficienc*" or "avitaminosis c" or "hypovitaminosis c").ti,ab,sh,kf |  |
| 53. | (hypoascorbemia* or scorbutus or scurvies or scurvy or barlow disease or scorbut).ti,ab,sh,kf |  |
| 54. | (wasting or wasted).ti,ab,sh,kf |  |
| 55. | (nutrition specific).ti,ab,sh,kf |  |
|  | **SEARCH SET 6 – HEALTH TERMS** | **RECORDS** |
| 1. | Exp Health Services/ |  |
| 2. | Exp Health Knowledge, Attitudes, Practice/ |  |
| 3 | Exp Health Promotion/ |  |
| 4 | (health service*).ti,ab,sh,kf |  |
| 5. | (telemedicine or telehealth or mobile health or mHealth or ehealth).ti,ab,sh,kf |  |
| 6. | (antenatal care or prenatal care or neonatal care).ti,ab,sh,kf |  |
| 7. | (health promot* or health educat* or health prevent* or universal health care).ti,ab,sh,kf |  |
| 8. | (early child* develop* or parent* practice* or develop* milestone* or growth monitoring).ti,ab,sh,kf |  |
| 10. | (deworm*).ti,ab,sh,kf |  |
| 11. | (family plan* or contraception*).ti,ab,sh,kf |  |
| 12. | (infectious disease* or communicable disease* or antimalarial or vaccin* or immune*).ti,ab,sh,kf |  |
| 13. | (malaria or ebola or west nile or typhoid or dengue or tuberculosis or HIV or measles or polio or mumps or rubella).ti,ab,sh,kf |  |
| 14. | (diarrhea or diarrhoea or rotavirus or rehydration).ti,ab,sh,kf |  |
| 15. | (upper respiratory infection* or URI or pneumonia).ti,ab,sh,kf |  |
| 16. | (low birth weight, LBW, preterm, small for gestation* or SGA).ti,ab,sh,kf |  |
|  | **SEARCH SET 7 – NUTRITION SENSITIVE TERMS** |  |
| 1. | (nutrition sensitive).ti,ab,sh,kf |  |
| 2. | (social safety net* or social protection or social assistance).ti,ab,sh,kf |  |
| 3. | ((extra or take home) adj5 (food* or ration*)).ti,ab,sh,kf |  |
| 4. | targeted food distribution*.ti,ab,sh,kf |  |
| 5. | ((transfer* or voucher*) adj5 (cash or money)).ti,ab,sh,kf |  |
| 6. | (food aid or food relief or food assistance or food stamp* or snap program* or supplemental nutrition assistance program* or wic program*).ti,ab,sh,kf |  |
| 7. | (enriched food* or food fortification or fortified food* or supplemented food*).ti,ab,sh,kf |  |
| 8. | fortified milk.ti,ab,sh,kf |  |
| 9. | general ration distribution.ti,ab,sh,kf |  |
| 10. | ((energy or protein or cereal) adj (bar or bars or biscuit* or cookie* or milk or food or foods)).ti,ab,sh,kf |  |
| 11. | (((lipid* based or energy) adj3 supplement*) or plumpy or "lns" or nutrispread or "nutri spread").ti,ab,sh,kf |  |
| 12. | ((ready to use adj3 (supplementary food* or therapeutic food*)) or rusf or rutf).ti,ab,sh,kf |  |
| 13. | (water sanitation hygiene or WASH).ti,ab,sh,kf |  |
| 14. | (empowerment |  |
| 15. | (agriculture or |  |
|  | **SEARCH SET 8 – INTEGRATED INTERVENTION TERMS** | **RECORDS** |
| 1. | Exp Delivery of Health Care, Integrated/ |  |
| 2. | (integrat* care or “integration of care” or integrat* services or “integration of services” or integrat* programme* or integrat* program* or “integration of programmes” or “integration of programs” or integrat* service delivery or “integration of service delivery” or integrat* service* or “integration of services” or integrat* delivery or integrat* management or “integration of  management” or integrat* intervention* or integrat* platform).ti,ab,sh,kf |  |
| 3. | (coordinat* care or “coordination of care” or coordinat* service* or “coordination of services” or coordinat* programme* or coordinat* program* or “coordination of programmes” or “coordination of programs” or coordinat* service delivery or “coordination of service delivery” or co ordinat* services or “coordination of services” or coordinat* delivery or coordinat*  management or “coordination of management” or co-ordinat* care or “co-ordination of care” or co-ordinat* services or “co-ordination of services” or co-ordinat* programmes or co-ordinat* programs or “co-ordination of programmes” or “co-ordination of programs” or co-ordinat* service delivery or “co-ordination of service delivery” or co-ordinat* services or “co-ordination of services” or co-ordinat*).ti,ab,sh,kf |  |
| 4. | (IMCI or iCCM).ti,ab,sh,kf |  |
|  | **SEARCH SET 9 - LMICs** | **RECORDS** |
| 1 | (afghanistan or algeria or angola or "antigua and barbuda" or antigua or barbuda or aruba or bahrain or bangladesh or barbados or british honduras or benin or dahomey or bhutan or bolivia or bechuanaland or burkina faso or burkina fasso or upper volta or burundi or urundi or cabo verde or cape verde or cambodia or kampuchea or khmer republic or cameroon or cameron or cameroun or central african republic or ubangi shari or chad or chile or comoros or comoro islands or iles comores or mayotte or democratic republic of the congo or democratic republic congo or congo or zaire or "cote d’ivoire" or "cote d’ ivoire" or cote divoire or cote d ivoire or ivory coast or croatia or cyprus or czech republic or czechoslovakia or djibouti or french somaliland or egypt or united arab republic or el salvador or spanish guinea or eritrea or estonia or eswatini or swaziland or ethiopia or gambia or ghana or gold coast or gibraltar or greece or guam or guinea or guinea bissau or haiti or hispaniola or honduras or hungary or india or indonesia or timor or iran or isle of man or kenya or "democratic people’s republic of korea" or republic of korea or north korea or south korea or korea or kyrgyzstan or kirghizia or kirgizstan or kyrgyz republic or kirghiz or laos or lao pdr or "lao people's democratic republic" or latvia or lebanon or lebanese republic or lesotho or basutoland or liberia or lithuania or macau or macao or madagascar or malagasy republic or malawi or nyasaland or indian ocean or mali or malta or micronesia or federated states of micronesia or kiribati or nauru or northern mariana islands or mauritania or mongolia or morocco or ifni or mozambique or portuguese east africa or myanmar or burma or nepal or netherlands antilles or nicaragua or niger or nigeria or oman or muscat or pakistan or panama or papua new guinea or new guinea or peru or philippines or philipines or phillipines or phillippines or poland or "polish people's republic" or portugal or portuguese republic or puerto rico or romania or rwanda or ruanda or samoa or pacific islands or polynesia or samoan islands or navigator island or navigator islands or "sao tome and principe" or saudi arabia or senegal or seychelles or sierra leone or slovakia or slovak republic or slovenia or melanesia or solomon island or solomon islands or norfolk island or norfolk islands or somalia or south sudan or sri lanka or ceylon or "saint kitts and nevis" or "st. kitts and nevis" or sudan or dutch guiana or netherlands guiana or syria or syrian arab republic or tajikistan or tadjikistan or tadzhikistan or tadzhik or tanzania or tanganyika or siam or timor leste or east timor or togo or togolese republic or "trinidad and tobago" or trinidad or tobago or tunisia or uganda or ukraine or uruguay or uzbekistan or uzbek or vanuatu or new hebrides or venezuela or vietnam or viet nam or middle east or west bank or gaza or palestine or yemen or yugoslavia or zambia or zimbabwe or northern rhodesia or global south or africa south of the sahara or sub-saharan africa or subsaharan africa or africa, central or central africa or africa, northern or north africa or northern africa or magreb or maghrib or sahara or africa, southern or southern africa or africa, eastern or east africa or eastern africa or africa, western or west africa or western africa or west indies or indian ocean islands or caribbean or central america or latin america or "south and central america" or south america or asia, central or central asia or asia, northern or north asia or northern asia or asia, southeastern or southeastern asia or south eastern asia or southeast asia or south east asia or asia, western or western asia or europe, eastern or east europe or eastern europe or developing country or developing countries or developing nation? or developing population? or developing world or less developed countr* or less developed nation? or less developed population? or less developed world or lesser developed countr* or lesser developed nation? or lesser developed population? or lesser developed world or under developed countr* or under developed nation? or under developed population? or under developed world or underdeveloped countr* or underdeveloped nation? or underdeveloped population? or underdeveloped world or middle income countr* or middle income nation? or middle income population? or low income countr* or low income nation? or low income population? or lower income countr* or lower income nation? or lower income population? or underserved countr* or underserved nation? or underserved population? or underserved world or under served countr* or under served nation? or under served population? or under served world or deprived countr* or deprived nation? or deprived population? or deprived world or poor countr* or poor nation? or poor population? or poor world or poorer countr* or poorer nation? or poorer population? or poorer world or developing econom* or less developed econom* or lesser developed econom* or under developed econom* or underdeveloped econom* or middle income econom* or low income econom* or lower income econom* or low gdp or low gnp or low gross domestic or low gross national or lower gdp or lower gnp or lower gross domestic or lower gross national or lmic or lmics or third world or lami countr* or transitional countr* or emerging economies or emerging nation?).ti,ab,sh,kf. |  |
|  | **SEARCH STRATEGY** |  |
|  | 1. SEARCH SET 1 OR SEARCH SET 2 OR SEARCH SET 3 2. SEARCH SET 5 AND SEARCH SET 6 3. SEARCH SET 5 AND SEARCH SET 7 4. SEARCH SET 6 AND SEARH SET 7 5. B OR C OR D 6. SEARCH SET 4 7. SEARCH SET 8 8. SEARCH SET 9 9. A AND E AND F AND G AND H |  |

**Supplementary Table 2. List of LMIC countries affected by conflict**

| **Low income-conflict countries**   - Afghanistan - Burkina Faso - Burundi - Central African Republic - Chad - DRC (Congo) - Ethiopia - Mali - Mozambique - Niger - Somalia - South Sudan - Sudan - Syria - Yemen |
| --- |
| **Lower-middle-income conflict countries**   - Cameroon - Egypt - Haiti - India - Indonesia - Iran - Kenya - Kyrgyzstan - Myanmar - Nigeria - Pakistan - Philippines - Ukraine - Venezuela - West Bank and Gaza |
| **LMIC countries with previous conflict**   - Liberia - Angola - Uganda |

**Supplementary Table 3.** List of integrated strategies by type of integration

| Type of integration | Program | Location | Year |
| --- | --- | --- | --- |
| Nsp + Nsn+ Health  (N=43) | ^1^Micronutrient and health (MICAH programme) | Ethiopia, Ghana, Malawi, Tanzania | 1996-2005 |
|  | ^2^Integrated Child Development Services | India | 2005-2016 |
|  | ^3^Comprehensive health cluster strategy | Pakistan | 2008-2009 |
|  | ^4^Health cluster approach and a package of vital PHC services | Pakistan | 2008-2009 |
|  | ^5^Integrated enhanced nutrition and responsive stimulation | Pakistan | 2009-2012 |
|  | ^6^Integrated enhanced nutrition and responsive stimulation delivered by LHWs | Pakistan | 2009-2012 |
|  | ^7^Baby tent program | Haiti | 2010-2012 |
|  | ^8^Enhanced homestead food production (E-HFP) program | Burkina Faso | 2010-2013 |
|  | ^9-11^Tubaramure program | Burundi | 2010-2014 |
|  | ^12^GFD + PHC treatment of malnutrition + vaccination + WASH services | Chad | 2010-2017 |
|  | ^13^Integrated health and nutrition interventions and reproductive health services | Kenya | 2011-2012 |
|  | ^14^Sweet potato Action for Security and Health in Africa (SASHA) | Kenya | 2011-2013 |
|  | ^15^Concern International Emergency Cash Transfer Program + health and nutrition education session | Niger | 2012 |
|  | ^16^Integration of MCN activities with health systems + specialized nutritious food through Posyandus + BCC to promote IYCF | Indonesia | 2012-2015 |
|  | ^17^Integration of polio immunization in health camps + community mobilization | Pakistan | 2013-2014 |
|  | ^18^Baby friendly spaces | Cameroon | 2014-2016 |
|  | ^19^BCC + community-led total sanitation and hygiene (CLTSH) activities | Ethiopia | 2015 |
|  | ^20,21^Double and standard cash transfers, fresh food vouchers | Pakistan | 2015-2016 |
|  | ^22^Maternal and Child Stunting Reduction Programme (MCSRP) | Pakistan | 2015-2018 |
|  | ^23^Regional EMOP 200777: cash transfers integrated with malnutrition prevention | Nigeria | 2016 |
|  | ^24^Swabhimaan program | India | 2016-2019 |
|  | ^25^Community Resilience to Acute Malnutrition (CRAM) | Chad | 2017 |
|  | ^26^Mental health and psychosocial support (MHPSS) interventions | Bangladesh | 2017-NR |
|  | ^27,28^Cash for Nutrition: CBT+ nutrition and health education, nutrition screening | Yemen | 2017-2020 |
|  | ^29^Emergency mobile medical teams (eMMT) | South Sudan | 2017-2020 |
|  | ^30^Comprehensive nutrition rehabilitation intervention: food supplementation and health messaging | Indonesia | 2018-2019 |
|  | ^31,32^Healthy Kitchen and Community-based School Nutrition Intervention | Lebanon | 2019 |
|  | ^33^Cash transfer + health screening + mHealth BCC interventions | Somalia | 2019 |
|  | ^34^Revised Emergency Nutrition response | Cameroon | 2019 |
|  | ^35^Integrated Mobile health teams (MHTs) | Afghanistan | 2019 |
|  | ^36^Integrated Reproductive, Maternal, Neonatal, Child Health and Nutrition | Pakistan | 2019 |
|  | ^37^WASH in Nut(WiN) | Niger | 2019 |
|  | ^38^Maternal and child nutrition project | India | 2019 |
|  | ^39^Ethiopia Nutrition Project (P106228) | Ethiopia | 2019 |
|  | ^40^Health and Nutrition Summer Camps | Lebanon | 2020 |
|  | ^41^Community-based nutrition package | Afghanistan | 2020 |
|  | ^42^Public Health Emergency Operation Center (PHEOC) | Ethiopia | 2020-2021 |
|  | ^43^Integrated Emergency, Health, Nutrition, Protection and WASH project | Syria | 2020-2021 |
|  | ^44^MNCP II – healthy IYCF, integrated nutrition services (NC and cash transfers), | Kenya | 2021 |
|  | ^45^WASH, Health and nutrition project | Sudan | 2021 |
|  | ^46^Integrated package of nutrition, food security, education, civic Education, youth empowerment, WASH, protection, and health intervention | Somalia | 2021 |
|  | ^47^Integrated WASH, health, nutrition, and multipurpose cash assistance (MPCA) programs | Sudan | 2022 |
|  | ^48^Integrated package of services | Mali | 2023 |
| Nsp + Health  (N=36) | ^49^Integration of nutritional supplementation into MNCH programs | DRC | 1994-1996 |
|  | ^50^Integration of VAS in polio campaigns | India | 2000 |
|  | ^51^Integration of VAS in routine-health services | Ethiopia | 2000-2015 |
|  | ^52,53^ Enhanced Integrated Nutrition and Health Program (INHP II) | India | 2004-2006 |
|  | ^54,55^Integrated Management of Neonatal and Childhood Illness (IMNCI) programme | India | 2004-2010 |
|  | ^56^Integration of VAS with deworming, immunization and ITN distribution | Ethiopia, Madagascar, Tanzania, Uganda, Zambia, Zimbabwe | 2006-2007 |
|  | ^57^Integrated Management of Neonatal and Childhood Illness (IMNCI) | India | 2008-2010 |
|  | ^58^Community-based supplementary feeding program | Uganda | 2010-2012 |
|  | ^59,60^Integration of OTPs and SAM treatment into the existing public health system including health posts | Ethiopia | 2011 |
|  | ^61^Integrated family planning and community-based nutrition programs | Ethiopia | 2012 |
|  | ^62^Integrated Child Development Services (ICDS) and National Rural Health Mission (NRHM) | India | 2013 |
|  | ^63^Emergency response – Red Cross | Philippines | 2013 |
|  | ^64^Community-health workers provided IYCF counselling at home | India | 2013-2014 |
|  | ^65^IMAM integrated with health services | Niger | 2013-2014 |
|  | ^66^Child Protection Project: Integration of VAS in polio campaigns | Angola, Chad, Cote d’Ivoire, Tanzania and Togo | 2014-2015 |
|  | ^67^Integrated Rapid Response Mechanism (IRRM) | South Sudan | 2014-2015 |
|  | ^68^Community based management of severe wasting | South Sudan | 2014-2020 |
|  | ^69^Joint Rapid Response Mechanism (RRM) | South Sudan | 2015 |
|  | ^70^Integration of VAS in routine-health services | Ethiopia | 2015 |
|  | ^71^Niger Maternal Nutrition (NiMaNu) project: BCC + provision of essential drugs | Niger | 2015-2017 |
|  | ^72^Integration of VAS with deworming | Kenya | 2016 |
|  | ^73^Acute malnutrition care at OTC level | Uganda | 2016 |
|  | ^74^Cross-sectoral communication for development (C4D) model | Kenya | 2016 |
|  | ^75^Community screening and treatment of malnutrition integrated with IYCF activities, VAS, IFA, MNP supplementation and deworming | Yemen | 2016 |
|  | ^76^Integration of routine health services and distribution of fortified cereal | Nigeria | 2016-2017 |
|  | ^77,78^Helping Babies Survive (HBS) program training in conflict settings | Somalia | 2016-2018 |
|  | ^79^IYCF programmatic activities and distribution of formula and milk | Lebanon | 2017 |
|  | ^80^Immunization services integrated with nutrition services in OTP centers and nutrition outreach | South Sudan | 2017 |
|  | ^81^Integration of VAS with SMC (SPAQ) | Nigeria | 2019 |
|  | ^82^ JEEViKA/Bihar Rural Livelihoods Project | India | 2019 |
|  | ^83^Integration of COVID-19 measures into Inpatient management of SAM | Yemen | 2020 |
|  | ^84^Postnatal home visiting programs | State of Palestine | 2020 |
|  | ^85^Integration of IYCF-E and nutrition into overall EVD response | DRC | 2020 |
|  | ^86^Integrated e-Diagnostic approach | Burkina-Faso | 2021 |
|  | ^87^IOM Health and Nutrition services | Somalia | 2022 |
|  | ^88^Integrated package of community and primary health interventions to strengthen delivery of RMNCAHN services | Nigeria | 2023 |
| Nsp + Nsn  (N=14) | ^89^Psychosocial activities + nutrition monitoring and education | Uganda | 2007-2008 |
|  | ^90^LNS + Super Cereal plus + UCT | Niger | 2011 |
|  | ^91^LNS Super Cereal + plus + cash transfers | Niger | 2011 |
|  | ^92^UCT + supplementary nutrition | Niger | 2011-2012 |
|  | ^93^Psychosocial activities + food rations + BCC | Burkina Faso, Niger, Mauritania, Chad and Senegal | 2013 |
|  | ^94^Home/preschool fortification with MNP + early learning interventions | India | 2013 |
|  | ^95^Healthy-Baby Flipbook for home-based IYCF counselling | Thailand | 2013-2014 |
|  | ^96^SAM treatment + counselling on IYCF + UCT | DRC | 2015-2016 |
|  | ^97^WASHplus | Mali | 2016 |
|  | ^98^Integrated package of complementary nutrition activities | Sudan | 2016-2017 |
|  | ^99,100^IYCF counselling + SQ-LNS distribution | DRC | 2016-2018 |
|  | ^101^Nutritional Counselling + UCT | Somalia | 2017-2019 |
|  | ^102^UCT + LNS + SBCC | Pakistan | 2017-2019 |
|  | ^103^Livestock for Health (L4H) | Kenya | 2019-2021 |
| *Abbreviations:* BCC, behavioural change communication; CBT, cash-based transfer; community management of acute malnutrition; EVD, Ebola virus diseases; GFD, general food distribution; IMAM, integrated management of acute malnutrition; IFA, iron folic acid; IOM, international organization of migration; IYCF, infant and young child feeding; IYCF-E, infant young child feeding education; ITN, insecticide treated bed nets; LHW, lady health worker; MCN, maternal and child nutrition; MNCH, maternal, newborn and child health; mHealth, mobile health; MNP, micronutrient powder; NC, nutritional counselling; Nsn, nutrition-sensitive; Nsp, nutrition-specific; OTC, outpatient therapeutic center; PHC, primary health care; RMNCAHN, reproductive, maternal, newborn, child and adolescent health and nutrition; LNS, lipid-based nutrient supplement; SAM, severe acute malnutrition; SBCC, social and behaviour change communication; SMC, seasonal malaria chemoprevention; SPAQ, sulfadoxine-pyrimethamine and amodiaquine; SQ-LNS, small quantity lipid-based nutrient supplement; UCT, unconditional cash transfer; VAS, vitamin A supplementation; WASH, water sanitation and hygiene. | | | |

**Supplementary Table 4.** Characteristics of Included Studies

| **Study ID** | **Study design** | **Period** | **Location** | **Conflict** | **Participants** | **Integrated Strategies** | **Outcomes** |
| --- | --- | --- | --- | --- | --- | --- | --- |
| Peer Reviewed literature from search of electronic databases up till March 2024 | | | | | | | |
| Alemu et al. (2022)  ^42^ | Descriptive | Dec 2020-Jun 2021 | Ranch collective site, Chagni, Ethiopia | Armed  (Internal conflict) | 79,041 IDPs (31,868 camp and 47,173 in host community) | **Nsp +Nsn + Health**  Public Health Emergency Operation Center (PHEOC) -coordination platform to deliver health + nutrition emergency responses | GAM (%), children under-5 with SAM (%), moderate malnutrition in PLW (%), vaccination (N) |
| Ali et al. (2022)  ^101^ | Quasi-experimental | Jul 2017-Feb 2019 | 2 districts in Banadir region, Somalia | Complex  (Conflict and drought) | Children aged six months up to five years who had mild or moderate malnutrition (mild, −1.0 to −1.9; moderate, −2.0 to 2.9 WHZ) and their caregivers.  Intervention group=116 children and 88 caregivers, Control=148 children, 87 caregivers | **Nsp + Nsn**  Nutritional counselling + unconditional cash transfer (NC+UCT) | Differences in wasting, underweight and stunting at follow-up, composite index of anthropometric failure, children’s minimum dietary diversity |
| Amsalu et al. (2020)  ^104^ | Quasi-experimental | Aug 2016-Dec 2018 | Bossaso, Somalia | Complex  (Climate shock and conflict) | IDPs and refugees | **Nsp + Health**  American Academy of Pediatrics **Helping Babies Survive (HBS)** program – breastfeeding counselling + maternal health + intrapartum care | Proportion of newborns who received essential newborn care, early initiation of breastfeeding, training knowledge scores, score on the accurate completion of a partograph, skills in newborn resuscitation, |
| Ayoya et al. (2013)  ^7^ | Descriptive | Feb 2010-June 2012 | 5 cities – Port-au-Prince, Haiti | Complex  (Earthquake, gang violence, civilian massacre) | 180 499 mother–infant pairs and 52 503 pregnant women registered in program | **Nsp + Nsn + Health**  Baby tent program - infant feeding support, growth monitoring + nutritional counselling + health assessment and management + psychosocial support | Number of tents established; Number of mother-infant pairs enrolled |
| Shaker-Berbari et al. (2017)  ^79^ | Cross-sectional? | 4yrs – funding dependent | 4 regions in Lebanon (North, Bekaa, South and Beirut and Mount Lebanon) | Armed  (Syrian Civil War) | Organisations active in the provision of humanitarian assistance to Syrian refugees in the areas of health, food security or nutrition | **Nsp + Health**  IYCF programmatic activities + general health services + reproductive health services | Number of organizations with internal IYCF policies, Number of organizations with external IYCF policies, Number of organizations distributing infant formula, Number of facilities targeted for distribution of milk powder, Number of infants benefiting from formula, Blanket or targeted distribution of infant formula |
| Bile et al. (2010)  ^3^ | Program evaluation | Crisis of 2008-2009 | Khyber Pakhtunkhwa (KP) and Federally Administered Tribal Areas (FATA), Pakistan | Armed  (Regional terrorism) | 446, 755 families internally displaced by the humanitarian crisis of 2008-09 (88% in host community and 12% in camps) | **Nsp + Nsn + Health**  Comprehensive health cluster strategy **-** nutrition surveillance + mass vaccinations + maternal and child health services + health promotion + WASH strategies + mental health and psychosocial support | Barriers and facilitators |
| Bile et al. (2011)  ^4^ | Descriptive | Crisis of 2008-2009 | Pakistan | Armed  (Regional terrorism) | 60% of the total 2.7 million IDPs were mothers and children | **Nsp + Nsn + Health**  Comprehensive health cluster strategy **-** nutrition surveillance + mass vaccinations + maternal and child health services + health promotion + WASH strategies + mental health and psychosocial support | Proportion of outbreaks of diarrhoea, VPD, HEP A and E. |
| Bliss et al. (2018)  ^15^ | Quasi-experimental | Jul-Sept 2012 | Villages in Niger | Complex  (Conflict and drought) | Female heads of 426 beneficiary households with at least one child aged 6-23 months of age within 21 selected villages | **Nsp + Nsn + Health**  Nutrition and health education + management of acute malnutrition + emergency cash transfer | Weight gain velocity, acute malnutrition, dietary diversity, meal frequency |
| Borja Jr et al. (2019)  ^26^ | Descriptive | 2017-not specified | Cox’s Bazaar, Bangladesh | Armed  (Genocide) | 700,000 Rohingya refugees (55% children) | **Nsp + Nsn + Health**  IYCF support and CMAM + mental health and psychosocial support (MHPSS) interventions + psychological first aid, | Community evaluation of MHPSS services |
| Chehab et al. (2016)  ^66^ | Program evaluation | 2014-15 | Angola, Chad, Cote d’Ivoire, Tanzania and Togo | Armed  (Civil war and insurgency) | Vit A supplementation: children aged 6-59 months  OPV: children aged 0-59 months | **Nsp + Health**  Vitamin A supplementation + polio immunization campaigns | Coverage of vitamin A supplementation, Campaign reach, wellbeing of children aged 4-10 |
| Codija et al. (2022)  ^44^ | Program evaluation | Jun-Jul 2021 | 3 counties Kitui, Isiolo and Turkana in Kenya | Armed  (Insurgency and ethnic conflict) | 167 participants were interviewed. Program beneficiaries were pregnant and lactating women, mothers of children under five years and children under five years | **Nsp + Nsn + Health**  IYCF counselling + vitamin A supplementation + CMAM + integrated cash transfers + integrated packages of services for children and women of reproductive age, focusing on health, nutrition, WASH, and HIV services | Proportion of children who received two annual doses of Vit A, proportion of pregnant women receiving IFA supplementation, number of children 6-59 months with SAM admitted for treatment |
| Coulibaly-Zerbo et al. (2021)  ^83^ | Descriptive | Adaption in May 2020, monitoring continued till Dec 2020 | Aden and Sana, Yemen | Complex  (Civil war, economic collapse, disease) | Children under-5 years of age suffering from food insecurity and at risk for malnutrition | **Nsp + Health**  Adaptation of the Nutrition Program under COVID-19: Integrating COVID-19 measures into Inpatient management of SAM + nutrition surveillance system (NSS) | Number of children screened by the Nutrition Surveillance System, Number of children admitted to TFC, |
| Dozio et al. (2020)  ^18^ | Descriptive | Oct 2014-Apr 2016 | 3 refugee camps in East  Cameroon (Timangolo, Lolo and M’bilé) | Armed | 1022 (203 pregnant women, 819 lactating women and 819 infants) | **Nsp + Nsn + Health**  Baby Friendly Spaces – IYCF counselling + care for women during pregnancy and after delivery + mother and child play sessions + psychosocial support + home visits + community awareness activities | Breastfeeding practices, psychological suffering (self-reported on a suffering scale), quality of mother-baby interactions (observation grid from Manual for the integration of childcare practices and mental health into nutrition programmes) |
| Dulacha et al. (2022)  ^29^ | Descriptive | 2017-2020 | Conflict-affected Kajo-Keji and Tambura, and flood-affected locations such as Pibor, Akobo and Mayom in South Sudan | Armed  (Sudanese Civil war) | IDPs | **Nsp + Nsn + Health**  Emergency mobile medical teams (eMMT) **–** integrated health and nutrition services including screening of malnourished cases and referrals + WASH interventions + routine vaccination + ANC care + health education and promotion + minor surgeries + reactive measles and cholera vaccination + pre-emptive cholera vaccination | Vaccination coverage – measles, reactive oral cholera, pre-emptive oral cholera, outpatient consultations, |
| El-Jor et al. (2021)  ^40^ | Quasi-experimental | 20 camps over 12 months. Camps were 2 weeks each. | Lebanon | Armed  (Syrian Civil War) | 295 students aged 9-16 years who attended the camps (Lebanese and Syrian) | **Nsp+ Nsn + Health**  Health and Nutrition Summer Camps – healthy snacks + nutrition education, health and life skills awareness + recreational and educational activities | Nutrition knowledge scores, Life skills knowledge scores, social cohesion measure |
| Fahmida et al. (2022)  ^105^ | Cluster RCT | Dec 2018  Feb-Sept 2019 | Villages in Lombok district, Indonesia | Complex  (Earthquake, terrorism) | 480 mothers (children 6-49 months) who came to the early childhood education centers (ECE) | **Nsp + Nsn +Health**  Comprehensive nutrition rehabilitation intervention – IYCF counselling + mothers received shredded fish/liver/anchovy + parenting classes + early childhood development and psychosocial stimulation | Dietary diversity scores in children under-2, WAZ in children 24 months or older, self-reported maternal depression |
| Fenn et al. (2017)  ^20^ | Cluster RCT | Jun 2015-Jun 2016 | 114 villages, Dadu District, Sind Province, Pakistan | Complex  (Armed conflict, environmental shocks) | 2496 poor and very poor households with 3584 children aged 6±48 mo and their female caregivers | **Nsp + Nsn + Health**  Cash-based incentives + fresh food vouchers + BCC for IYCF + treatment of SAM + BCC for WASH | Prevalence of wasting, stunting |
| Fenn et al. (2021)  ^12^ | Program evaluation | 2010-2017 | 12 UNHCR mandated refugees camps in eastern Chad - Amnabak and Kounoungo, Gozamer; Amnabak, Iridimi and Touloum;Djabal; Bredjing; Farchana;Gaga; Mile; Oure Cassoni; Treguine | Armed  (Inter-and intra-state rebel groups) | 39231 records of children 6–59 months registered with the UNHCR | **Nsp + Nsn + Health**  General food distribution + IYCF programmes + management of acute malnutrition + primary health care + vaccinations + WASH services | Prevalence of wasting, stunting in children aged 0-23 and 24-59 months |
| Gatobu et al. (2017)  ^70^ | Program evaluation | 2015 | Amhara region, Ethiopia | Armed  (Ethnic, state- and non-state rebel groups) | Children aged 6-59 months | **Nsp + Health**   - VAS via routine health services - VAS via Community Health Day Campaign | Coverage of VAS |
| Ghattas et al. (2019)  ^106^ | Quasi-experimental | 8 months | 2 Palestinian refugee camps in Beirut, Lebanon | Complex  (Protracted conflict in the region) | 33 women participated in the kitchens on a rotating basis to provide daily healthy snacks to 847 children attending two local elementary schools | **Nsp + Nsn + Health**  Healthy Kitchen and Community-based School Nutrition Intervention -  Subsidized healthy food + health, hygiene and nutrition education for children and parents + women empowerment | Program participation |
| Sahyoun et al. (2019)  ^32^ | Secondary report - Ghattas et al. 2019 | 8 months | 2 Palestinian refugee camps in Beirut, Lebanon (Bourj el Barajneh and Shatila) | Complex  (Protracted conflict in the region) | 33 women participated in the kitchens on a rotating basis to provide daily healthy snacks to 847 children attending two local elementary schools | **Nsp + Nsn +Health**  Healthy Kitchen and Community-based School Nutrition Intervention -  Subsidized healthy food + health, hygiene and nutrition education for children and parents + women empowerment | 7-point Arab Family Food Security Scale score, women’s mental health inventory (MHI-5) score |
| Gorstein et al. (2003)  ^50^ | Program evaluation | Mar-Aug 2000 | Odisha state, India | Complex  (Cyclone, state/ethnic violence) | 1811 children aged 12-48 months | **Nsp + Health**  High dose vitamin A supplementation on national immunization day + intensified pulse polio immunization (IPPI) | Prevalence of Bitot’s spot, low serum retinol (< 0.70 μmol/L), severe vitamin A deficiency (serum retinol < 0.35 μmol/L), underweight, stunting, coverage of VAS |
| Grellety et al. (2017)  ^96^ | RCT | Jul 2015-Jul 2016 | Bipemba (commune in the city of Mbuji-Mayi) in the Kasaï Oriental province, Democratic Republic of Congo | Armed  (Interstate and rebel groups) | 1481 children aged 6-59 months eligible for outpatient SAM treatment | **Nsp + Nsn**  Treatment of SAM + IYCF counselling + cooking demonstrations + cash transfer | Changes in WAZ score/month, changes in MUAC/day, changes in food consumption scores, changes to individual dietary scores, relapse to MAM, relapse to SAM, |
| Grijalva-Eternod et al. (2023)  ^33^ | Cluster RCT | Jan 2019-Sep 2019 | Afgooye Corridor, in peri-urban Mogadishu, Somalia | Complex  (Protracted conflict and climate shocks) | Households in IDP camps located between 0.3 and 1 km straight-line distance from the Concern Worldwide–operated health centre with children aged 0-59 months | **Nsp + Nsn + Health**  mHealth BCC messaging on IYCF, vaccination, health, and WASH + cash transfers | Coverage of measles immunization, % vaccinated as per Somalia Expanded Programme on Immunisation (EPI) schedule, pentavalent series completion, child dietary diversity scores, prevalence of exclusive breastfeeding |
| Habib et al. (2017)  ^17^ | Cluster RCT | Jun 2013-May 2014 | Bajaur, Kashmore, Karachi Districts, Pakistan | Complex  (Ongoing insurgency, general insecurity) | 75189 healthy children aged 1mo-5yrs that resided within the study sites in three districts of Pakistan at high risk of polio | **Nsp + Nsn + Health**  Health camps – Polio immunization + maternal and child health services + nutrition counselling + WASH messaging | Coverage of OPV, IPV  Proportion fully immunized, partially immunized and not immunized |
| Hashmi et al. (2018)  ^95^ | Descriptive | Oct 2013-Jun 2014 | Mae La refugee camp (MLA), Thailand | Armed  (Genocide) | 20 mother-infant pairs who were Rohingya refugees | **Nsp + Nsn**  Home-based counselling – IYCF counselling + WASH messaging | Proportion of infants exclusively breastfed, Infants at 6 and 9 months of age who were fed adequate dietary diversity, safe disposal of infant stool |
| Ickes et al. (2020)  ^11^ | Commentary on Leroy et al. 2018 | 4 years | Rural Burundi | Complex  (civil war, rebel forces, political instability) | 4960 households surveyed in 2012, 6946 households in 2014 | **Nsp + Health**  Food rations (corn-soy blend and micronutrient-fortified vegetable oil) + BCC on nutrition and health + promotion of health services | Proportion of food secure households, impact on stunting, anemia. Children’s dietary diversity |
| Kurdi et al. (2020)  ^27^ | Cluster RCT | 2.5 years | 3 districts in Al Hodeidah governorate, Yemen | Armed  (Civil war) | 1945 households with female relatives of Social Welfare fund beneficiaries who were pregnant or had children under 2 years of age | **Nsp + Nsn + Health**  Nutrition and health education sessions + WASH education + malnutrition screening sessions + cash transfers | Probability of BF initiation within the 1^st^ hour after delivery. Probability of exclusive BF during the first 6 months, Program impact on early BF, exclusive BF, total knowledge scores, knowledge on exclusive BF, treated water for children, |
| Laillou et al.(2020)  ^51^ | Descriptive | DHS surveys of 2000, 2005, 2011 and 2016  (15 years) | Ethiopia | Complex  (Ethnic, state- and non-state rebel groups, droughts) | Children 6-59 months of age | **Nsp + Health**  Vitamin A supplementation on Child Health Days + integration with routine health services at health posts | Coverage rates of VAS, VAS inequities by wealth and rural-urban residence |
| Langendorf et al. (2014)  ^91^ | Quasi-experimental | Aug-Dec 2011 | Madarounfa health district, in the  southern part of Maradi region, Niger | Armed  (state and non-state-based violence) | 5395 children aged 6-23 months living in 48 villages and hamlets | **Nsp + Nsn**  Lipid-based nutrient supplements/Super Cereal/Food rations + cash transfers | Adjusted risk (HR) of MAM, SAM and mortality |
| Leidman et al. (2017)  ^76^ | Cross-sectional | Oct 2016-Mar 2017 | Northeastern states of Borno and Yobe, Nigeria | Armed  (State and non-state violence, militant groups, Islamic insurgency) | Children aged 0-59 months in Borno (round 1: 1557, round 2: 1813) and Yobe (round 1: 1692, round 2: 2729) | **Nsp + Health**  Nutritional screening + fortified cereals + MNCH services + vaccination | Proportion of households in each region that received fortified cereals in the last 6 months (Round 1 and 2), access to ORS among children with diarrhoea aged 0-59 months |
| Leroy et al. (2020)  ^10^ | Cluster RCT | 4 years | Provinces of Cankuzo and Ruyigi in eastern Burundi | Armed  (rebel forces, political violence) | Pregnant women (at or after the 4^th^ month of gestation) and children aged less than 6 months  3572 households in 2010, 2614 households in 2012 | **Nsp + Nsn + Health**  Food rations + BCC strategy for nutrition, health, and WASH + promotion of health services + training of health staff | Household Food Insecurity Access Scale (HIFAS) score, HHS score, diet diversity score, maternal diet diversity score (including/excluding corn soy blend), total energy consumed (kcal) |
| Leroy et al. (2016)  ^9^ | RCT | 4 years  Baseline: 2010  Follow-up:  2012  And 2014 | Provinces of Cankuzo and Ruyigi in eastern Burundi | Armed  (rebel forces, political violence) | Baseline: 2566 households, follow-up: 2581 households | **Nsp + Nsn + Health**  Food rations + BCC strategy for nutrition, health, and WASH + promotion of health services + training of health staff | Percent consumed CSB in the past 24h, dietary diversity with CSB/without CSB, consumed iron rich foods with CSB/without CSB, minimum meal frequency |
| Locks et al. (2019)  ^99^ | Cross-sectional | 2 years | DRC | Armed  (interstate and rebel groups) | 660 households were targeted in each health zone during both baseline (1288 mothers) and endline surveys (1307 mothers) | **Nsp + Nsn**  IYCF counselling + SQ-LNS distribution + WASH messaging | Adjusted comparision (DID) Early initiation of breastfeeding, water not introduced till 6 months, solid foods introduced at 6 months, child breastfeeding/complementary feeding, minimum diet diversity, minimum meal frequency, WASH indicators, proportion of mothers received LNS, child consumption of LNS, mother participation |
| McGrath et al. (2014)  ^93^ | Descriptive | 6 weeks | **Burkina Faso, Niger,** Mauritania, **Chad** and Senegal | Complex  (Interstate and non-state rebel groups, food crisis) | 1^st^ author visited the five countries in the region for six weeks with UNICEF | **Nsp + Nsn**  Food rations + nutrition BCC sessions + psychosocial support | Number of participants |
| Mokori et al. (2013)  ^58^ | Program evaluation | May 2010-Mar 2012 | 17 sub-counties in northern Uganda | Armed (political instability, interstate and non-state rebel groups) | 363 households and 412 children aged 6-59 months were recruited | **Nsp + Health**  Screening for malnutrition + supplementary feeding program + nutrition education + immunization + health promotion | Prevalence of moderate and severe wasting, prevalence if moderate and severe underweight |
| Morris et al. (2012)  ^89^ | Program evaluation | May 2007-Jan 2008 | 5 established feeding centers in the Kitgum district, Northern Uganda | Armed  (political instability, interstate and non-state rebel groups) | 237 mothers with either MAM or SAM infants aged between 6 and 30 months entering the combined psychosocial and feeding programs | **Nsp + Nsn**  Nutrition and IYCF education + psychosocial support | Maternal ability to stimulate child (HOME scale), maternal mood (Kitgum maternal mood scale) |
| Oiye et al. (2019)  ^72^ | Cross-sectional | Jun-Jul 2016 | 3 counties in western Kenya | Armed  (non-state, state-based and Islamic insurgency) | 1177 caregivers of children aged 6-59 months | **Nsp + Health**  Vitaimin A supplementation + deworming | Vitamin A knowledge and coverage, prevalence of deworming in children aged 12-59 months in the past 6 months and twice yearly, time to nearest health facility and age-appropriate VAS (</>45min) |
| Oladeji et al. (2019)  ^80^ | Descriptive | Jan-Dec 2017 | Bentiu PoC site in Unity State, South Sudan | Armed  (civil war) | Sector 2: 20, 315  Sector 2: 33, 379  Total population  Sector 2: 1646  Sector 5: 2712  Children under 5 years seen at the OTP centres and during nutrition community outreaches | **Nsp + Health**  Treatment of acute malnutrition at OTP centres + nutrition education and counselling + immunization services | Children vaccinated in sector 2 and 5 in 2016 compared with 2017, probability of missed vaccination among children, immunization coverage (BCG, OPV3, PENTA3, MEASLES) among malnourished children at OTP centers in sector 2 and 5 |
| Oresanya et al. (2022)  ^81^ | Program evaluation | Jul-Nov 2019 | Nigeria | Armed  (state and non-state violence, militant groups, insurgency) | 197 caregivers from 33 communities in 11 wards were interviewed | **Nsp + Health**  Vitamin A supplementation + seasonal malaria chemoprevention | VAS coverage, SPAQ for SMC coverage, awareness of vitamin A and seasonal malaria chemoprevention |
| Renzaho et al. (2021)  ^68^ | Program evaluation | 2014-2020 | South Sudan | Armed  (Civil war) | 1, 105, 546 children were admitted to CMSW programs | **Nsp + Health**  Treatment of severe acute malnutrition + IYCF counselling + vitamin A supplementation + health services | Crude mortality rate per 10,000 live births per day, under-five mortality per 10,000 live births per day, trends in prevalence of wasting, relapse rate, recovery rate in CMSW programs, children aged 6-59 months screened for wasting, wasted children treated caregivers receiving MNCN education, children aged 6-69 months supplemented with vitamin A during IRRM missions, weight gain velocity |
| Renzaho et al. (2003)  ^49^ | Cross-sectional | 1994-1996 | DRC (Zaire) | Complex  (military conflict, active volcanoes) | 31, 714 children aged 6-59 months in Katale camp | **Nsp + Health**  Treatment of malnutrition integrated into the MNCH programs | Children aged 9-59 months vaccinated against measles, crude mortality rate per 10,000 per day in 1994 compared to 1996 |
| Scott et al. (2017)  ^90^ | Quasi-experimental | Aug-Dec 2011, Feb-Mar 2012 | Madarounfa district in Maradi region, Niger | Armed  (state and non-state-based violence) | 124 female caregivers of 3192 children aged 6-23 months in eight villages | **Nsp + Nsn**  Supplementary food ration + nutrition education + cash transfer | Proportion of transfer spent on the target child |
| Sibson et al. (2018)  ^92^ | Cluster RCT | Mar 2015-Nov 2015 | Niger | Environmental (food crisis) | Children aged 6–59 months, living in villages - sampled 1959 children and obtained baseline measures from 1831 (2093 intervention, 495 control) | **Nsp + Nsn**  Supplementary food rations + nutrition education + cash transfers | Global acute malnutrition, WHZ, prevalence of stunting (crude, partially and fully adjusted), prevalence of low MUAC (crude, partially adjusted, fully adjusted), Dietary diversity |
| Soofi et al. (2022)  ^102^ | Cluster RCT | May 2017-Jul 2019 | Rahim Yar Khan, Pakistan | Complex  (terrorism, border conflict, floods) | 1729 children at 6 months of age living in the poorest households in Rahim Yar Khan | **Nsp + Nsn**  Lipid-based nutrient supplements + SBCC sessions on IYCF and WASH + cash transfers | Risk of stunting at 24 months of age (Rate Ratio), prevalence of stunting among children 6-23 months of age, pair-wise intervention comparisons for prevalence of stunting |
| Thumbi et al. (2023)  ^103^ | Field RCT | Sept 2019- Dec 2021 | Laisamis region of  Marsabit County in northern Kenya | Armed  (Islamic insurgency and tribal conflict) | 1734 pregnant women/mothers and 1748 children under 3-years of age with strong dependence on livestock | **Nsn + Nsp**  Enhanced nutritional counselling and education + livestock feed + hygiene messaging | Barriers and facilitators of program implementation |
| Trenouth et al. (2018)  ^21^ | Secondary report of Fenn et al. 2017 | 6 months | Dadu, Pakistan | Armed  (terrorism, border conflict) | 3562 children aged 6-48 months from 2496 poor households | **Nsp + Nsn + Health**  BCC on nutrition, IYCF, and WASH + fresh food vouchers + cash transfers | DiD in prevalence of wasting and stunting, costing |
| Wanzira et al. (2018)  ^73^ | Cross-sectional | Jul-Aug 2016 | 6 government owned health facilities in Arura district, West Nile region, Uganda | Armed  (government and rebel forces, refugees from Sudan and DRC) | Population sample from the 6 health facilities accounted for 45.4% of the total caseload of malnourished children | **Nsp + Health**  Treatment of severe acute malnutrition + nutrition counselling + evaluation of HIV + evaluation of health outcomes | Performance of facilities according to the NSDA tool, cure rate, defaulting rate and quality of case management (correct diagnosis, treatment, counselling, HIV evaluation, exit outcome) at all 6 health facilities |
| Peer Reviewed literature from snowball search | | | | | | | |
| Berti et al. (2010)  ^1^ | Program evaluation | 1996-2005  (10 years) | **Ethiopia**, Ghana, Malawi, Tanzania | Armed  (Internal ethnic conflict and conflict with Eritrea) | 4 million direct and more than 6 million indirect benefiricaries (pre-school and school age children, pregnant, post-partum and women of childbearing age) | **Nsp + Nsn + Health**  Micronutrient supplement distribution + dietary diversification + IYCF counselling + WASH strategies + immunization + diarrhea management + malaria prevention | Impact on immunization nationally, impact on exclusive BF, impact on anemia and malaria, impact on vitamin A, iodine status, impacts on height and weight |
| Chakrabarti et al. (2019) ^2^ | Program evaluation – secondary analysis | 2005-2006  2015-2016 | India | Armed  (conflict with neighbouring countries, ethnic and religious conflict) | 82 million children younger than 6 years and over 19 million PLW | **Nsp + Nsn + Health**  Integrated Child Development Services - Take-home supplementary food + health and nutrition education + health check-ups + immunization + pre-school care services | % change in intervention coverage during pregnancy, lactation and childhood between 2006 and 2016 |
| Deconinck et al. (2016)  ^65^ | Program evaluation | 2013-2014 | Aguie Health District in Maradi Region, Matameye Health District in Zinder Region and Niger overall | Complex  (State and non-state-based violence, drought) | Interviews with 57 community members and 5 FGDs | **Nsp + Health**  IMAM integrated with health services: | Uptake of IMAM and community perception |
| Doherty et al. (2010)  ^56^ | Program evaluation | Nov 2006-Mar 2007 | **Ethiopia,** Madagascar, Tanzania, Uganda, Zambia, Zimbabwe | Armed  (Internal ethnic conflict and conflict with Eritrea) | 28 interviews conducted per country | **Nsp + Health**  Child Health Days – Vitamin A supplementation + immunization + deworming + distribution of ITNs | Effects of CHDs on coverage of key child survival interventions |
| Fagerli et al. (2017)  ^13^ | Program evaluation | Mar 2011-Mar 2012 | Suba and Mbita districts in western Kenya. | Armed  (Islamic insurgency and tribal group conflict) | 328 pregnant women >/=14 years of age presenting to ANC clinics in the 12 selected health facilities | **Nsp + Nsn + Health**  Integrated health and nutrition interventions and reproductive health services – Fortified flour distribution, WASH training, free hygiene kids, improving obstetric care, maternal screening for syphilis | Coverage of program interventions (hygiene kits, protein-fortified flour, clean delivery kits), impact on handwashing, Coverage of ANC visits, health facility delivery, postnatal checkup |
| Fernandez-Rao et al. (2013)  ^94^ | RCT (individual and cluster) | NR | Rural villages of Nalgonda District of Andhra Pradesh, India | Armed  (conflict with neighbouring countries, ethnic and religious conflict) | Individual:  Children aged 6-12 months or  Cluster:  36-48 months in 23 selected villages | **Nsp + Nsn**  Fortification with micronutrient powders + counselling on complementary feeding and dietary diversification + early childhood development | Barriers and facilitators to program implementation |
| Gowani et al. (2014)  ^5^ | RCT – Cost and effects | Jul 2009-Mar 2012 | Rural Sindh, Pakistan | Complex  (insurgency, general insecurity, drought) | Birth cohort of 1489 children | **Nsp + Nsn + Health**  Fortification of MNP + hygiene and health messages + responsive stimulation + treatment of minor ailments + family planning + immunization + ANC | Barriers and facilitators to program implementation |
| Head et al. (2019)  ^19^ | Case control | Jun-Jul 2015 | 2 woredas of Oromia, Ethiopia | Armed  (Ethnic, state and non-state rebel groups) | Mothers of children aged 0-59 months in 1007 households | **Nsp + Nsn + Health**  Screening and treatment of SAM + vegetable seed provision + promotion of backyard gardening + IYCF education + community-led total sanitation and hygiene (CLTSH) activities + health promotion | Prevalence of stunting, two-week history of fever, |
| Kim et al. (2017)  ^62^ | Descriptive | Jul-Aug 2013 | 3 districts in Odisha, India | Armed  (conflict with neighbouring countries, ethnic and religious conflict) | 145 semi-structured interviews with three types of CHWs from 12 villages | **Nsp + Health**  Maternal iron-folic acid supplementation + vitamin A supplementation + IYCF counseling + referrals for SAM + ANC + immunization | Barriers and facilitators to program implementation |
| Levin et al. (2019)  ^14^ | Program evaluation – cost | Mar 2011-Dec 2013 | Kenya | Armed  (Islamic insurgency and tribal group conflict) |  | **Nsp + Nsn + Health**  Counselling of pregnant women on nutrition and healthy eating + orange-fleshed sweet potato vine vouchers + ANC | Utilization of agricultural vouchers |
| Mukhopadhyay et al. (2017)  ^64^ | RCT | Jan 2013-Feb 2014 | Gangajal Ghati Community Development Block in Bankura district of West Bengal, India | Armed  (conflict with neighbouring countries, ethnic and religious conflict) | Birth cohorts from 6 sub-centers | **Nsp + Health**  IYCF counselling + health counselling | Awareness of early initiation of BF, knowledge regarding optimum duration of exclusive BF, correct knowledge on timely introduction of semi-solid food, exclusive BF in optimum frequency at 6^th^ month, average gain in infant length and weight at 6^th^ month |
| Ouedraogo et al. (2018)  ^71^ | Quasi-experimental | 19 months | Two health districts  (Mirriah and Zinder) in the Zinder region, Niger | Complex  (State and non-state-based violence, drought) | 2307 pregnant women in the selected villages | **Nsp + Health**  BCC on nutrition during pregnancy + provision of essential drugs and supplies + improvement in ANC | Proportion of pregnant women who attended any ANC, who attended a gestational-age specific adequate number of ANC, received any IFA supplements, receiving and consuming IFA daily, adequate GWG per week, adequate MUAC |
| Singh et al. (2017)  ^52^ | Quasi-experimental | May 2004-Jul 2006 | 2 rural districts in Uttar Pradesh, India | Armed  (conflict with neighbouring countries, ethnic and religious conflict) | 3^rd^ trimester pregnant women | **Nsp + Health**  BCC focusing on IYCF + postnatal care + immunization | Contact with a health worker in 3^rd^ trimester, Anganwadi worker home visit in 3^rd^ trimester, recall of education about BF practices, recall of advice about CF practices, health VAS, pediatric IFA supplementation, adjusted odds of optimal breastfeeding initiation practices |
| Tadesse et al. (2017)  ^59^ | Cohort | Jul-Dec 2011 | 4 adjacent districts of Wolaita zone in southern Ethiopia | Armed  (ethnic, state- and non-state, rebel groups) | 1659 children admitted to OTPs in 94 health posts | **Nsp + Health**  Integrated OTPs and SAM treatment into the existing health system/health posts | Proportion of children with SAM after discharge, readmission to program after discharge, status on discharge/exit outcomes |
| Yousafzai et al. (2014)  ^6^ | RCT | Jun 2009-Mar 2012  33 mo | Naushero Feroze, Sindh, Pakistan | Complex  (Ongoing insurgency, general insecurity) | 3550 children across all clusters from a single birth cohort, 1489 mother-infant dyads were enrolled for in-depth assessment | **Nsn + Nsp + Health**  Nutrition/IYCF education + MNP fortification + health and hygiene education + responsive stimulation | Households received nutritional advice on last home visit, 30-day dose of MMN powder delivered, households which received MNP and did not give MNP to children, HAZ, developmental outcomes at 12 months of age |
| Non-peer reviewed literature from search of humanitarian organization websites and other grey literature sources | | | | | | | |
| ENN-1  Ngwenyi et al. (2019)  ^34^ | Field Exchange article | Jul 2019 | Far North region, East and Adamaoua regions, Cameroon | Complex  (climatic shocks, sociopolitical crisis, insurgency) | Refugees and IDPs and the host resident population both in and outside camps | **Nsp + Nsn + Health**  Distribution of Super Cereal Plus for management of MAM + SBCC focused on IYCF and WASH, general food distribution, childhood diseases management, immunization, deworming, malaria prevention, MMN supplementation, family planning services | Coverage of health districts, total cost per beneficiary, prevalence of low MUAC coverage of SNF |
| ENN-2  Noorzad et. al. (2020)  ^41^ | Original research article | Jun 2020 | Afghanistan | Complex  (Political conflict, prone to recurrent natural disasters) | 2 million children under 5 years old, their caregivers and other male community members who are main decision-makers at household level | **Nsp + Nsn + Health**  Community-based health and nutrition services, IYCF support, cooking demonstrations, management of acute malnutrition, immunization | Coverage of home visits by CHW, number of growth monitoring promotion sessions, costing |
| ENN-3 Marshaket al. (2017)  ^25^ | Program impact assessment | 2017 | 69 settlements encompassed by the Concern programme area in Dar Sila region, eastern Chad | Complex  (Variable rainfall, seasonal food insecurity, | 1,400 households spread evenly between 69 settlements consisting of IDPs and host communities | **Nsp + Nsn + Health**  Community Resilience to Acute Malnutrition (CRAM): Integrated program nutrition and health + WASH and food, income and markets | Illness of child in past two weeks, prevalence of wasting, stunting, WHZ scores |
| ENN-4  Mwendwa et al. (2016) ^74^ | Field Exchange article | 2016 | Dadaab refugee camp, Kenya | Complex  (famine, refugees fleeing armed conflict) | 349,280 is the total population of registered refugees as of Aug 2015 | **Nsp + Health**  Strengthening, integrating, and sustaining MIYCN interventions within mainstream health and nutrition programs of partner organizations like IRC, MSF, IRK | Barriers and facilitators to program implementation |
| ENN-5  Abdullah et al. (2016)  ^75^ | Evaluations and Lessons Learned report | 2016 | Hodeidah and Hajjah sub cluster, Yemen | Complex  (political crisis) | 8.4 million people lack access to basic healthcare | **Nsp + Health**  Community screening + IYCF activities + capacity building of TFC + MMN, IFA and Vitamin A supplementation + deworming + maternal and neonatal care | Coverage of SAM treatment, OTP services, MAM treatment, number of breastfeeding corners in health facilities, Prevalence of GAM, SAM, underweight, stunting |
| ENN-6  Qarizada et al. (2019)  ^35^ | Field Exchange article | 2019 | 4 districts of Faryab province, northern Afghanistan | Complex  (Political conflict, prone to recurrent natural disasters) | 66,590 children aged 6-59 months | **Nsp + Nsn + Health**  Integrated Mobile health teams (MHTs) – health and nutrition services + IYCF counselling + immunization, + WASH messaging | Number of people reached with health and nutrition services, prevalence of MAM, number of people reached with IYCF counselling, immunization rates |
| ENN-7  Ahmed et al. (2019)  ^36^ | Original research article | Jun 2019 | Punjab, Pakistan | Complex  (border conflict and natural disaster) | Approximately 1.9 million PLW in Punjab, Pakistan are being screened by LHW | **Nsp + Nsn + Health**  Integrated Reproductive, Maternal, Neonatal, Child Health and Nutrition – IFA supplementation + IYCF counselling + MNCH care | Coverage of ANC from skilled provider, proportion of deliveries assisted by a skilled birth attendant, proportion of facility deliveries, number of IFA tablets provided |
| ENN-8  Jan Badar et al. (2020)  ^22^ | Original research article | 2015-2018 | 3 districts in Sindh province, Pakistan | Complex  (border conflict and natural disaster) | 800,000 target population (children under 2 years and PLW) in all 3 districts | **Nsp + Nsn + Health**  Maternal and Child Stunting Reduction Programme (MCSRP): IYCF + WASH interventions including SBCC, community engagement, and counselling | Number of people reached with nutrition-specific interventions, costing |
| ENN-9  Tamboura et al. (2019)  ^37^ | Field Exchange article | 2019 | Niger | Armed  (state and non-state-based violence) | 322,381 people | **Nsp + Nsn + Health**  Management of acute malnutrition + WASH interventions + provision WiN (WASH in Nutrition)  hygiene kits + treatment of diarrhea with ORS, zinc salts + community awareness sessions | Prevalence of SAM, diarrhoea, acute respiratory infections, malaria, other morbidities, coverage of nutrition programming |
| ENN-10  (HKI)  Olney et al. (2015)  ^8^ | Cluster RCT | 2 years | Burkina-Faso | Complex  (political instability, drought) | All women with children aged 3-13 months in 55 villages in 4 departments in Gourma province, 1767 households at baseline and 1481 at endline | **Nsp + Nsn + Health**  Enhanced homestead food production (E-HFP) program: Nutrition + health strategies + integrated agricultural production activities (provision of seeds, saplings, chicks and gardening tools and training) | Prevalence of anemia, diarrhea in children, decrease in prevalence of wasting, impacts on dietary intake diversity, |
| ENN-11  (CINI)  Mukherjee et al. (2019)  ^38^ | Pilot project evaluation | 2019 | 3 districts in West Bengal, India | Complex  (violence, natural disasters) | 210 women in the 1^st^ trimester of pregnancy from all 98 ICDS centers | **Nsp + Nsn + Health**  Dietary counselling of pregnant women + nutritional counselling of family members + monthly weight monitoring for assessing GWG + coupon incentives + ANC + maternal health counselling | Prevalence of maternal anemia, rate of institutional deliveries, results of initial nutritional assessment |
| ENN-12  Bery et al. (2016) ^97^ | Field Exchange article | 2016 | 180 villages in 18 communes in northern Mali | Armed  (Mali War) | 187,000 women of reproductive age and 60,000 of their children in poor, rural households and communities | **Nsp + Nsn**  Promote exclusive breastfeeding + nutrition demonstrations + WASH strategies (latrine construction rehabilitation of water points, important hygiene messaging) | Number of MAM referrals, number of SAM referrals, |
| ENN-13  Shrivatsav et al. (2021)  ^24^ | Midline program evaluation | 2016-2021  Midline:  2019 | 3 states in India | Complex  (Violence, natural disasters) | 3171 adolescent girls, 1856 pregnant women and 3,277 mothers of children under 2 years | **Nsp + Nsn + Health**  Swabhimaan program - IFA, calcium supplementation, counselling on dietary diversity, use of iodised salt, food subsidy schemes, BPE, kitchen gardens, deworming, access to ITNs | Number of women who received ANC, use of modern family planning methods, number of women screened using MUAC, proportion of thinness and severe thinness, costs for scale-up, number of nutrition gardens developed, minimum dietary diversity, IFA compliance, proportion of women who received one dose of albendazole |
| ICRC-14  Messoudi et al. (2013)  ^63^ | Operational update | May-2013 | Zamboanga, Philippines | Armed  (Communist rebels, insurgency) | 9000 displaced people and 50,6000 people in evacuation centers | **Nsp + Health**  VAS, nutrition assessment, emergency latrine building and clean up campaign deworming, basic health care, mass immunization | Number of people reached through interventions in the emergency response |
| World Vision-15  ^43^ | Fiscal Year 2020 results report | 2020-2021 | Syria | Armed  (Civil war) | 900,000 IDPs being added to 2.7 million in need of humanitarian assistance | **Nsp + Health**  Integrated Emergency, Health, Nutrition, Protection and WASH project | Number of people with access to health, WASH and protection services, number of caregivers with access to preventive emergency nutrition services, Number of households visited by CHW teams |
| UNICEF-16  ^84^ | Field report | 2020 | Gaza strip, Palestine | Armed  (Genocide) | 55,000 deliveries take place annually in a health facility | **Nsp + Health**  Breastfeeding support + neonatal weight monitoring + postnatal home visit programs + assessment for neurodevelopmental delays + provision of postnatal kits | Number of mothers reached, impact on exclusive BF |
| UNICEF-17  ^85^ | Technical brief | Nov-2020 | DRC | Complex  (disease outbreak, armed conflict) | Mothers infected with EVD and over 3,000 affected infants and young children | **Nsp + Health**  IYCF-E and nutrition integrated into overall EVD response interventions | Number of infants and young children who had received BMS support |
| WB-18  ^39^ | Project Performance Assessment Report (PPAR) by the Independent Evaluation Group (IEG) of the World Bank Group | 2019 | Ethiopia | Armed (internal ethnic clashes) | 55.8 million people including PLW and under-5 children in 238 woredas | **Nsp + Nsn + Health**  Ethiopia Nutrition Project - IYCF BCC counseling + provision of micronutrients + growth monitoring + treatment of malnourished children + WASH strategies + basic healthcare and deworming | Number of people with access to CBN nutrition services, exclusive BF at the national level, proportion of women eating less during pregnancy, share of children who consume a minimum acceptable diet, diet diversity for under-two children, proportion of under-two children participating in monthly growth monitoring and promotion sessions |
| WB-19  ^82^ | Programme report | 2019 | Bihar, India | Armed  (internal ethnic clashes) | Pregnancy women and mothers of children less than 2 years in villages | **Nsp +Health**  JEEViKA/Bihar Rural Livelihoods Project - counselling for maternal nutrition + ensuring early registration of pregnancy check-ups + institutional delivery and birth-preparedness | Number of children reached, impacts on women and children’s diet diversity |
| WB-20  Sellen et al. (2012)  ^61^ | Discussion paper on a qualitative study | 2012 | Ethiopia | Armed conflict  (Internal clashes, ethnic violence) | Kebele A=1796, B=3285, C=2547, D=7569 | **Nsp + Health**  Integrated family planning and community-based nutrition programs - delivered messages on nutrition, sanitation, hygiene, malaria prevention and HIV/AIDS | Barriers and facilitators to program implementation |
| WFP-21  Brewin et al. (2017)  ^98^ | Evaluation report | 2016-2017 | Sudan | Armed  (political instability) | 2.3 million IDPs in Sudan and 386,000 refugees from neighbouring countries | **Nsp + Nsn**  Integrated package of complementary activities – GFD + food assistance for assets and food assistance for training (FFA/FFT) + School Meals + WASH messages | Coverage of program, MAM treatment recovery rate, MAM treatment mortality rate, |
| WFP-22  Dillon et al. (2016)  ^16^ | Evaluation report | 2012-2015 | Timor Tengah Selatan (TTS)  district, Nusa Tenggara Timur (NTT) Province, Indonesia | Complex  (flooding, landslides and drought exacerbated by climate change) | 893 children aged 18-35 months from 34 enrolled MCN program villages | **Nsp + Nsn + Health:**  Provision of specialized nutritious food for young children and PLW + IYCF BCC + WASH messaging + improve care-seeking behaviours + health messaging | Prevalence of diarrhoea, prevalence of stunting, wasting, underweight, LBW, anemia, number of children provided with fortified food rations, coverage of fortified biscuits, prevalence of timely introduction of complementary foods, uptake of program and growth monitoring |
| WFP-23  (WFP, UNICEF)  ^69^ | Analysis report | May 2015 | Juba, Greater Upper Nile region in Unity, Upper Nile and Jonglei states, South Sudan | Armed conflict  (Civil war) | Over 100,000 people displaced by the conflict sheltered in Protection of Civilians sites (PoCs) hosted by the UNMISS | **Nsp + NSn + Health**  Joint Rapid Response Mechanism (RRM) integrated package of life-saving humanitarian relief including GFD + curative nutrition services + immunizations for children + help for communities to access safe water + child education and protection services | Number of children vaccinated against measles, polio, Number of children screened for malnutrition, number of children reached by GFD, number of children who received supplementary feeding, costing |
| WFP-24  ^23^ | Situation report | Nov 2016 | Nigeria | Armed conflict  (Islamic insurgency) | 1.8 million IDPs are reported to be in north-east Nigeria | **Nsp +Nsn + Health**  Prevention of malnutrition programs + in-kind distributions + supplementary feeding programs + cash-based transfers + livelihood support + basic health care | Number of people who received food rations, number of children who received supplementary nutritious food, number of people who received in-kind food assistance, number of people who received CBT, |
| WFP-25  ^67^ | Evaluations and Lessons Learned | 2014-2015 | South Sudan | Armed  (Civil war) | 1,489,948 people including 278,776 children under 5 years | **Nsp + NSn + Health**  Integrated Rapid Response Mechanism (IRRM): Food assistance + nutrition, health and WASH strategies +, education + child protection. | Number of children vaccinated for measles, polio, Number of people screened for acute malnutrition, prevalence of SAM, MAM, number of PLW who received IYCF counselling, number of children who received VAS and deworming treatment, number of clean delivery kits provided |
| RW-26  (UNICEF)  ^48^ | Appeal | Jun 2023 | Mali | Complex  (Sociopolitical instability, intercommunal clashes, adverse weather, protracted effects of COVID-19 pandemic) | 8.8 million people in need in Jan 2023 with 375,539 IDPs | **Nsp + Nsn + Health**  Early detection and provision of quality nutrition care to wasted children +VAS + MNP distribution + cash transfers + social assistance support + national immunization campaigns + outbreak response and essential MNCH services | Children with SAM admitted for treatment, primary caregivers received IYCF counselling, vaccination rates for measles, polo, children receiving MNP, VAS, households receiving cash transfer/social assistance |
| RW-27  (WRC)  ^88^ | Evaluation and Lessons Learned Report | April 2023 | Borno state, Nigeria | Armed  (state and non-state violence, militant groups, insurgency) | 1.7 million IDPs as of 2021 | **Nsp + Health**  Integrated package of community and primary health interventions to strengthen delivery of RMNCAHN services including facilitating transport to health facility, supply chain strengthening, quality improvement | Facilitators and barriers to program implementation |
| RW-28  (IOM)  ^87^ | Quantitative survey, Assessment | Dec 2022 | Bardhere and Dollow, Somalia | Complex  (Violent conflict and severe drought) | 26,452 children between 6-59 months of age were screened for malnutrition in IDPs and host communities | **Nsp + Health**  Community screening and referrals for SAM + IYCF interventions + health education | SAM, MAM program coverage |
| RW-29  (CARE, USAID)  Ahmed et al. (2020)  ^45^ | Evaluation and Lessons Learned Report | Sept 2021 | South and East Darfur, Sudan | Armed  (political instability) | 443,190 individuals 332,764 individuals in South (including 253,191 IDPs and 79,573 host community members) and 110,426 individuals in East Darfur (including 10,000 IDPs and 100,426 host community members). | **Nsp + Nsn + Health**  Improved access to nutrition assistance for under-five children and PLW + increasing access to safe water supply, sanitation facilities and hygiene supplies + improving access to basic curative and preventative primary healthcare | Number of infants who received minimum diet diversity, mortality due to specific diseases, coverage of training on infant attitude change, prevalence of exclusive BF, coverage of ANC, PNC, malnourishment treatment, prevalence of malnourishment in children under-5, impact of health messaging |
| RW-30  (SOYDA)  ^46^ | Situation report | Oct-Dec 2021 | Somalia | Complex  (Armed conflict, climatic shocks, COVID-19 pandemic, desert locusts and poor rains from drought) | 838,900 children under the age of five years face acute malnutrition between Jan-Dec 2021, community members in Benadir, Southwest and Jubbaland State of Somalia. | **Nsp + Nsn + Health**  Integrated package of nutrition, Food Security, Education, Civic Education, Youth empowerment, WASH, protection, and health intervention | Coverage of VAS, measles and other immunization, number of children and PLW provided with malnourishment treatment, number of women who completed all 4 ANC visits |
| RW-31  (SUN)  Sarrassat et al. 2021  ^86^ | Analysis | Oct-2021 | Burkina-Faso | Complex  (Interstate and non-state rebel groups, food crisis) | 1755 (84%) of the country’s primary healthcare facilities | **Nsp + Health**  Integrated e-Diagnostic approach: digital tool for nutrition service delivery integrated into health infrastructure | Number of consultations conducted using the app, Number of PHCs using the app, SAM identification rate, reduction in antibiotic over prescription |
| RW-32  (CARE)  Elmedani et al. 2022  ^47^ | Evaluation and Lessons Learned Report | Sept 2022 | South and East Darfur, Sudan | Armed conflict  (state and non-state rebel groups, political instability) | 527,764 people from targeted conflict affected IDPs, resident and host community households | **Nsp + Nsn + Health**  Integrated nutrition + WASH + multipurpose cash assistance (MPCA) + health programs | Number of people reached, Number of consultations, Number of people benefiting from preventative and curative BHA nutrition supported services, Number of people admitted at nutrition centers (recovery, relapse and death rates), impact on children under-5 with SAM, uptake of ANC services, immunization rates, coverage of multi-purpose cash assistance |
| **Abbreviations**: ANC, antenatal care; BCC, behaviour change communication; BMS, breast milk substitutes; CARE, Cooperative for Assistance and Relief Everywhere; CINI, Child in Needs Institute; CHW, community health workers; CSB, corn-soy blend; EVD, Ebola Virus Disease; ENN, Emergency Nutrition Network; FA-MCHN, Food-assisted Maternal and Child Health and Nutrition; GFD, General Food Distribution; HQ-LNS, high-quantity lipid-based nutrient supplement; HKI, Helen Keller International; IHC, integrated health center; IOM, International Organization for Migration; ITN, insecticide-treated bed nets; ICDS, Integrated Child Development Services; MAM, moderate acute malnutrition; MIYCN, Maternal; Infant; and Young Child Nutrition; MOH, Ministry of Health; MNP, micronutrient powder; MMN, multiple-micronutrient; NR, not reported; NSDA, nutrition service delivery assessment; NSP, nutrition specific; NSN, nutrition-sensitive; OTC, outpatient therapeutic care; PLW, pregnant and lactating women; QI, quality improvement; RCT, randomized controlled trial; RMNCAHN, reproductive; maternal; newborn; child; and adolescent health and nutrition services; SC+, Super Cereal+; SMC, seasonal malaria chemoprevention; SPAQ; SAM, severe acute malnutrition; TFC, Therapeutic Feeding Centres; UNICEF, United Nations Children's Fund; UNMISS, United Nations Mission in South Sudan; USAID, United States Agency for International Development; WRC, Women's Refugee Commission; and μmol/L, micromolar per liter. | | | | | | | |

**Supplementary Table 5.** Description of nutritional supplementation strategies

| Study ID | Dose of supplement | Frequency of distribution | Conditionality |
| --- | --- | --- | --- |
| Lipid-nutrient supplementation | | | |
| Langendorf et al. (2014) ^91^ | Targeted: HQ-LNS (92 g/500kcal/day) (Plumpy’nut®; Nutriset) or MQ-LNS (46 g/250kcal/day) (Plumpy’Doz®;Nutriset) or 200 g/ day of SC+ (820kcal/day) | Monthly | Some groups received cash transfer of $52 and one group received household support in the form of food; comprising; cereals; pulses and oils |
| Locks et al. (2019) ^99^ | SQ-LNS (local name – Kulabora) | Monthly – 28 sachets | None |
| Fenn et al. (2021) ^12^ | Blanket: General Food Distribution (Full ration: 2100kcal/person/day) + 20 g daily ration of SQ-LNS (110 kcal/day) or 200 g/child/day of SC+  Targeted: Treatment of children affected with SAM using therapeutic milks (F75, F100) and RUTF. Treatment of children affected with MAM using CSB or ready-to-use supplementary foods | Daily (all children aged 6-59 mo) | None – families in camp |
| Soofi et al. (2022) ^102^ | MQ-LNS (local name – Wawamum) | Monthly – 30 sachets (from 6 to 24 mo) or daily | None – unconditional cash transfer ($32/quarter) and/or SBCC |
| Supplemental food (Blanket and Targeted) | | | |
| Renzaho et al. (2003) ^49^ | Blanket: General food distribution of CSB or BP-5 compact food (high-protein biscuits) (6240kJ energy/person/day)  Targeted: Special food distribution of micronutrient-fortified blended food (based on cereals such as flours and grains; legumes; and oil seeds) and high protein biscuits for children affected with MAM | Daily | None |
| UNICEF-WFP; 2015 | Blanket: General food distribution  Targeted: supplementary feeding for children affected with MAM and therapeutic care for children affected with SAM | Routinely | None |
| Leroy et al. (2016) ^9^ | Blanket: Household food ration of 12kg of micronutrient fortified CSB and 1200g of vitamin A enriched oil. Mothers individually received 6kg of CSB and 600g of oil during pregnancy and post-natal for 6 months | Monthly | None - Care group attendance was not mandatory |
| Fagerli et al. (2017) ^13^ | Blanket: All mothers received protein-fortified flour | At 2^nd^ and 4^th^ ANC visit | As incentive for attendance at maternal health services |
| Sibson et al. (2018) ^92^ | Blanket: 200 g/day SC+ for each child 6–<24 months (providing 820 kcal/day) and 250 g/day Super Cereal and 75 g/day vegetable oil for each pregnant/lactating woman (providing 1,613 kcal/day). | Monthly | Only between June and September. Intervention groups also received a cash transfer. Beneficiaries first had to attend an education session |
| Chakrabarti et al. (2019) ^2^ | Blanket: Take-home supplementary food and hot cooked meals | Routinely | Health and nutrition education and check-ups delivered at rural child-care centers or at home |
| Ghattas et al. 2019 ^106^ | Blanket: Health snacks sold in schools at a subsidized price | 5 days per week | Women received 1-week of training. Families receiving assistance from the social welfare program were exempt from the costs of the snacks |
| El-Jor et al. (2021) ^40^ | Blanket: Health snacks given for free at summer camps | Every day of camp | Participation in summer camp |
| Alemu et al. (2021)^42^ | Blanket: Each household received food ratio of 15kg/person/month an additional food commodity given regularly to prevent acute malnutrition  Targeted: Fortified foods for mothers and children affected with MAM | Monthly | None – families in camp |
| Fahmida et al. (2022) ^105^ | Blanket: Local food prepared by mothers and volunteer health cadres: liver, anchovies, fish | Twice weekly | Mothers attended parenting classes at Early Childhood Education (ECE) centers |
| Morris et al. (2012) ^89^ | Targeted: Community-based emergency feeding – RUTF, supplementary food | Weekly/fortnightly | Health education sessions on the topics of feeding practices, HIV, contraception, hygiene, and illness in the child |
| Mokori et al. (2013) ^58^ | Targeted: Pre-mix of supplementary food ration (CSB, vegetable cooking oil and sugar) | Bi-monthly | Nutrition education, VAS, deworming |
| Grellety et al. (2017) ^96^ | Targeted: Take-home ration of RUTF supplying approximately 170 kcal per kilogram per day (Plumpy’nut®, Nutriset) for children affected with SAM | Monthly | Caretakers with one or more children with SAM received an unconditional $40 cash transfer during treatment and follow-up |
| Tadesse et al. (2017) ^59^ | Targeted: OTP integrated in the primary healthcare system providing RUTF, medication and counselling for children affected with MAM | Routinely | None |
| Bliss et al. (2018) ^15^ | Targeted: CMAM which provides therapeutic and supplementary feeding for qualifying children | Routinely | Conditional emergency cash transfer and nutritional education sessions |
| Micronutrient supplementation | | | |
| Fernandez-Rao et al. (2013) ^94^ | Blanket: Locally manufactured MNP (Grow Smart 1 and 2 - 0.5 and 1g scoops) | Every 15 days – 15 sachets | At home/in pre-school after instruction on use |
| Yousafzai et al. (2014) ^6^ | Blanket: Locally manufactured MNP (Sprinkles®) | Monthly | Delivered by CHW during home visit after nutritional education |
| Gowani et al. (2014) ^5^ | Blanket: MNP (Sprinkles®) | Monthly | Nutrition education |
| UNICEF, 2023 | Blanket: Children 6-59 months receiving micronutrient powder | Routinely | None |
| Locally prepared foods (health snacks and cooked meals) and commercially available supplements (Therapeutic milks: F75, F100 Super Cereal Plus (SC+) or Corn Soy Blend Plus (CSB++), Plumpy®)  *Abbreviations:* ANC, antenatal care; CHW, community-health worker; CMAM, community management of acute malnutrition, HQ, high-quantity; LNS, lipid-nutrient supplement; MQ, medium-quantity; MAM, moderate acute malnutrition; MNP, micronutrient powder; RUTF, ready-to-use therapeutic food; SBCC, social behaviour change communication; SAM, severe acute malnutrition; SQ, small-quantity; VAS, vitamin A supplementation | | | |

**Supplementary Table 6.** Coverage rates for micronutrient supplementation, immunization under different integration strategies

| Study ID | Region, Country | Integration strategy | Coverage achieved (year) |
| --- | --- | --- | --- |
| Coverage of immunization | | | |
| Renzaho et al (2003) ^49^ | Democratic Republic of Congo | Measles immunization with treatment of malnutrition | 72% (1994)  88.6% (1996) |
| Doherty et al (2010) ^56^ | Ethiopia, Madagascar, Tanzania, Uganda, Zambia, Zimbabwe | Measles immunization with VAS, deworming and distribution of insecticide treated nets during Child Health Day campaign | 35% (Ethiopia)  61% (Madagascar)  68% (Uganda) |
| Habib et al. (2017) ^17^ | Pakistan | OPV immunization, with community mobilization and health camps vs routine immunization | 82% vs 75% |
|  |  | IPV delivered at the MCH immunisation strategy camps | 94.3% |
| Oladeji et al (2019) ^80^ | South Sudan | Measles and DPT3 immunization with treatment of malnutrition at OTP centers and nutrition education and counselling | 69.1-90% (2017) |
| Dulacha et al. (2022) ^29^ | South Sudan | Reactive measles campaign during outbreak delivered by mobile medical team | 72-125% (2019-2020) |
|  |  | Pre-emptive oral cholera vaccine delivered by mobile medical team | 89.3% |
|  |  | Immunization services integrated with nutrition services in OTP centers and nutrition outreach | Measles – 90%-69.1% (2017) |
| Grijalva-Eternod et al. (2023) ^33^ | Somalia | Measles immunization with conditional cash transfer | 39.2 (March 2019)  77.5% (Dec 2019) |
| Coverage of twice-yearly VAS in children aged 12-59 months | | | |
| Gorstein et al. (2003) ^50^ | Odisha, India | VAS + intensified pulse polio immunization on Child Health Day | 59.8% (March 1999)  97% (March 2000) |
| Chehab et al (2016) ^66^ | Chad, Angola, Togo, Tanzania and Cote d’Ivoire | VAS + OPV during campaigns | 80% (2013-2014) |
|  | Tanzania | VAS + Measles during campaigns | 100% (2013-2014) |
| Gatobu et al (2017) ^70^ | Amhara, Ethiopia | VAS during Child Health Day campaign vs VAS during routine health services | 91.7% vs 85.3% (Median coverage in 2015-2016) |
| Singh et al (2017) ^52^ | Uttar Pradesh, India | Integrated health and nutrition program vs standard nutrition program | 70.3% vs 32% (2004-2006) |
| Oiye et al. (2019) ^72^ | Kenya | VAS during immunization campaigns delivered at Early Childhood Development Centers | 64.5% (2018)  70% (2020) |
|  |  | VAS + deworming | 44.9% |
| Laillou et al. (2020) ^51^ | Ethiopia | VAS + Child Health Day campaign gradually integrated with routine health services at health posts | 93.1% (2006)  66.6% (2018) |
| Renzaho et al. (2021) ^68^ | South Sudan | VAS during medical missions | Ranged between 29.5% and 65.5%  50.8% (2014)  49.9% (2020) |
| Oresanya et al. (2022) ^81^ | Nigeria | VAS + seasonal malarial chemoprevention | 1.6% (Baseline)  59.4% (Endline) |
| *Abbreviations:*DPT, diphtheria, pertussis and tetanus; IPV, inactivated polio-virus vaccine; MCH, maternal and child health; OPV, oral polio-virus vaccine; OTP, outpatient therapeutic program; VAS, vitamin A supplementation | | | |

**Supplementary Table 7. PRISMA Checklist**

| **Section and Topic** | **Item #** | **Checklist item** | **Location where item is reported** |
| --- | --- | --- | --- |
| **TITLE** | | |  |
| Title | 1 | Identify the report as a systematic review. | Title |
| **ABSTRACT** | | |  |
| Abstract | 2 | See the PRISMA 2020 for Abstracts checklist. |  |
| **INTRODUCTION** | | |  |
| Rationale | 3 | Describe the rationale for the review in the context of existing knowledge. | 109-115 |
| Objectives | 4 | Provide an explicit statement of the objective(s) or question(s) the review addresses. | 118-127 |
| **METHODS** | | |  |
| Eligibility criteria | 5 | Specify the inclusion and exclusion criteria for the review and how studies were grouped for the syntheses. | 148-222 |
| Information sources | 6 | Specify all databases, registers, websites, organisations, reference lists and other sources searched or consulted to identify studies. Specify the date when each source was last searched or consulted. | 134-147 |
| Search strategy | 7 | Present the full search strategies for all databases, registers and websites, including any filters and limits used. | Table S1. |
| Selection process | 8 | Specify the methods used to decide whether a study met the inclusion criteria of the review, including how many reviewers screened each record and each report retrieved, whether they worked independently, and if applicable, details of automation tools used in the process. | 226-232 |
| Data collection process | 9 | Specify the methods used to collect data from reports, including how many reviewers collected data from each report, whether they worked independently, any processes for obtaining or confirming data from study investigators, and if applicable, details of automation tools used in the process. | 232-236 |
| Data items | 10a | List and define all outcomes for which data were sought. Specify whether all results that were compatible with each outcome domain in each study were sought (e.g. for all measures, time points, analyses), and if not, the methods used to decide which results to collect. | 233-235 |
|  | 10b | List and define all other variables for which data were sought (e.g. participant and intervention characteristics, funding sources). Describe any assumptions made about any missing or unclear information. | 233-235 |
| Study risk of bias assessment | 11 | Specify the methods used to assess risk of bias in the included studies, including details of the tool(s) used, how many reviewers assessed each study and whether they worked independently, and if applicable, details of automation tools used in the process. | 257-264 |
| Effect measures | 12 | Specify for each outcome the effect measure(s) (e.g. risk ratio, mean difference) used in the synthesis or presentation of results. | 242-249 |
| Synthesis methods | 13a | Describe the processes used to decide which studies were eligible for each synthesis (e.g. tabulating the study intervention characteristics and comparing against the planned groups for each synthesis (item #5)). |  |
|  | 13b | Describe any methods required to prepare the data for presentation or synthesis, such as handling of missing summary statistics, or data conversions. | 244-249 |
|  | 13c | Describe any methods used to tabulate or visually display results of individual studies and syntheses. | 242-253 |
|  | 13d | Describe any methods used to synthesize results and provide a rationale for the choice(s). If meta-analysis was performed, describe the model(s), method(s) to identify the presence and extent of statistical heterogeneity, and software package(s) used. | 242-253 |
|  | 13e | Describe any methods used to explore possible causes of heterogeneity among study results (e.g. subgroup analysis, meta-regression). | 248-249 |
|  | 13f | Describe any sensitivity analyses conducted to assess robustness of the synthesized results. | None |
| Reporting bias assessment | 14 | Describe any methods used to assess risk of bias due to missing results in a synthesis (arising from reporting biases). | 257-264 |
| Certainty assessment | 15 | Describe any methods used to assess certainty (or confidence) in the body of evidence for an outcome. | None |
| **RESULTS** | | |  |
| Study selection | 16a | Describe the results of the search and selection process, from the number of records identified in the search to the number of studies included in the review, ideally using a flow diagram. | 272-280 |
|  | 16b | Cite studies that might appear to meet the inclusion criteria, but which were excluded, and explain why they were excluded. | 274-275 |
| Study characteristics | 17 | Cite each included study and present its characteristics. | Table 2 |
| Risk of bias in studies | 18 | Present assessments of risk of bias for each included study. | Table S.3-S.5 |
| Results of individual studies | 19 | For all outcomes, present, for each study: (a) summary statistics for each group (where appropriate) and (b) an effect estimate and its precision (e.g. confidence/credible interval), ideally using structured tables or plots. | 410-654 |
| Results of syntheses | 20a | For each synthesis, briefly summarise the characteristics and risk of bias among contributing studies. | 508-545 |
|  | 20b | Present results of all statistical syntheses conducted. If meta-analysis was done, present for each the summary estimate and its precision (e.g. confidence/credible interval) and measures of statistical heterogeneity. If comparing groups, describe the direction of the effect. | 508-545 |
|  | 20c | Present results of all investigations of possible causes of heterogeneity among study results. | 508-545 |
|  | 20d | Present results of all sensitivity analyses conducted to assess the robustness of the synthesized results. | N/A |
| Reporting biases | 21 | Present assessments of risk of bias due to missing results (arising from reporting biases) for each synthesis assessed. | Table S.3-S.5 |
| Certainty of evidence | 22 | Present assessments of certainty (or confidence) in the body of evidence for each outcome assessed. | N/A |
| **DISCUSSION** | | |  |
| Discussion | 23a | Provide a general interpretation of the results in the context of other evidence. | 795-842 |
|  | 23b | Discuss any limitations of the evidence included in the review. | 844-906 |
|  | 23c | Discuss any limitations of the review processes used. | 724-732 |
|  | 23d | Discuss implications of the results for practice, policy, and future research. | 908-921 |
| **OTHER INFORMATION** | | |  |
| Registration and protocol | 24a | Provide registration information for the review, including register name and registration number, or state that the review was not registered. | 129-130 |
|  | 24b | Indicate where the review protocol can be accessed, or state that a protocol was not prepared. | 129-130 |
|  | 24c | Describe and explain any amendments to information provided at registration or in the protocol. | 182-193 |
| Support | 25 | Describe sources of financial or non-financial support for the review, and the role of the funders or sponsors in the review. | 940 |
| Competing interests | 26 | Declare any competing interests of review authors. | 939 |
| Availability of data, code and other materials | 27 | Report which of the following are publicly available and where they can be found: template data collection forms; data extracted from included studies; data used for all analyses; analytic code; any other materials used in the review. | 935-938 |


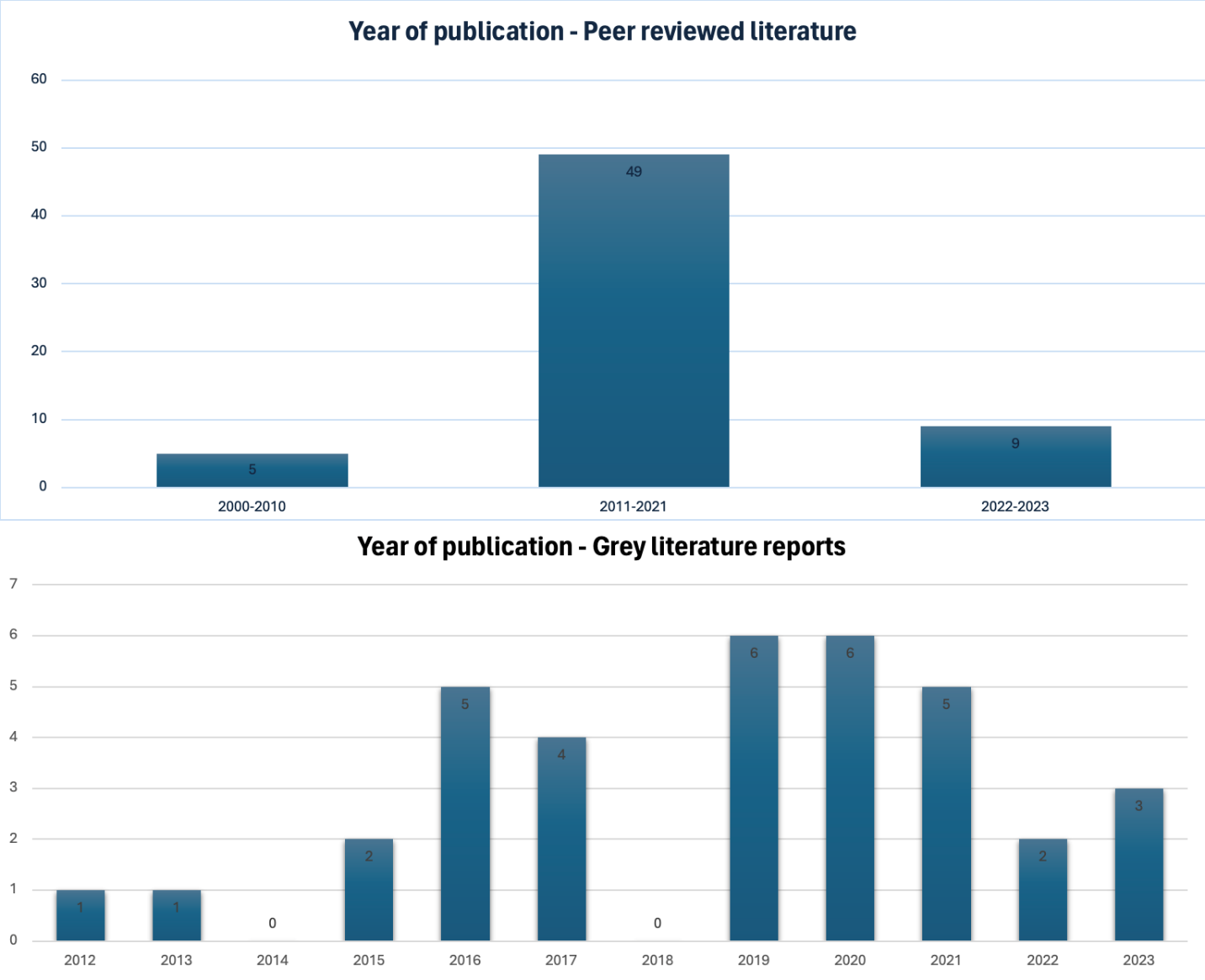


**Supplementary Figure 1. Included studies by publication year**


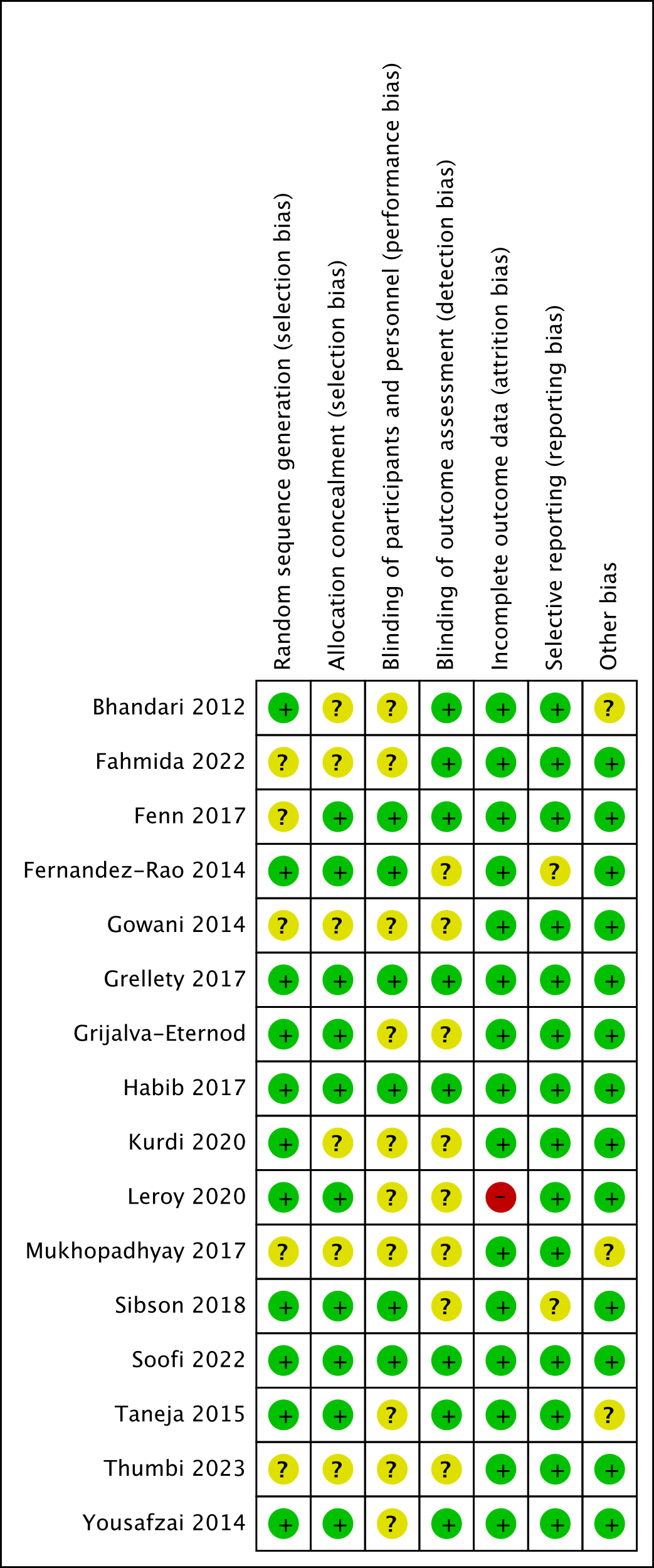


**Supplementary Figure 2 Risk of bias (RoB-2) summary: review authors' judgements about each risk of bias item for each included RCT** ^5,6,10,17,20,27,33,54,57,64,92,94,96,102,103,105^

| **Study ID** | **Confounding** | **Selection** | **Intervention** | **Attrition** | **Measurement** | **Reporting** | **Overall** |
| --- | --- | --- | --- | --- | --- | --- | --- |
| Ali et al. (2022) ^101^ | Low | Low | Low | Moderate | Low | Low | Moderate |
| Amsalu et al. (2020) ^104^ | Low | Moderate | Low | Moderate | Moderate | Low | Moderate |
| Bliss et al. (2018) ^15^ | Moderate | Moderate | Moderate | Low | Moderate | Low | Moderate |
| El-Jor et al. (2021) ^40^ | Low | Low | Low | Moderate | Moderate | Low | Moderate |
| Ghattas et al. (2019) ^106^ | Moderate | Low | Low | Low | Low | Low | Moderate |
| Langendorf et al. (2014) ^91^ | Moderate | Moderate | Moderate | Low | Low | Low | Moderate |
| Ouedraogo et al. (2018) ^71^ | Low | Moderate | Low | Moderate | Low | Low | Moderate |
| Scott et al. (2017) ^90^ | Moderate | Moderate | Low | Low | Low | Low | Moderate |
| Singh et al. ( 2017) ^52^ | Low | Moderate | Low | Moderate | Low | Low | Moderate |

**Supplementary Figure 3. Risk of bias for non-randomized studies (ROBINS-1) summary: review authors' judgements about each risk of bias item for each included quasi-experimental study**

| Study ID | Overall Quality Score |
| --- | --- |
| Head et al. (2019) ^19^ | Fair |

**Supplementary Figure 4. Risk of bias summary for case-control studies (NIH tool): review authors' judgements about each risk of bias item for each included case-control study**

| Study ID | Overall Quality Score |
| --- | --- |
| Shaker-Berbari et al. (2017) ^79^ | Medium |
| Berti et al. (2010) ^1^ | Medium high |
| Bile et al. (2010) ^3^ | Medium |
| Chakrabarti et al. (2019) ^2^ | Medium |
| Chehab et al. (2016) ^66^ | Medium |
| Codjia et al. (2022) ^44^ | Medium |
| Deconinck et al. (2016) ^65^ | Medium |
| Doherty et al. (2010) ^56^ | Medium |
| Fagerli et al. (2017) ^13^ | Medium |
| Fenn et al. (2021) ^12^ | Medium |
| Gatobu et al. (2017) ^70^ | Medium |
| Gorstein et al. (2003) ^50^ | Low-medium |
| Levin et al. (2019) ^14^ | Medium |
| Leidman et al. (2017) ^76^ | Medium |
| Locks et al. (2019) ^99^ | Low-medium |
| Mokori et al. (2013) ^58^ | Medium |
| Morris et al. (2012) ^89^ | Medium |
| Oiye et al. (2019) ^72^ | Low-medium |
| Oresanya et al. (2022) ^81^ | Medium |
| Renzaho et al. (2003) ^49^ | Medium |
| Renzaho et al. (2021) ^68^ | Low-medium |
| Tadesse et al. (2017) ^59^ | Medium-high |
| Wanzira et al. (2018) ^73^ | Medium |

**Supplementary Figure 5. Risk of bias for program evaluations, cohort and cross-sectional studies (NIH tool) summary: review authors' judgements about each risk of bias item for each included observational cohort and cross-sectional study**

References

1. Berti PR, Mildon A, Siekmans K, Main B, Macdonald C. An adequacy evaluation of a 10-year, four-country nutrition and health programme. *Int J Epidemiol*. Apr 2010;39(2):613-29. doi:10.1093/ije/dyp389

2. Chakrabarti S, Raghunathan K, Alderman H, Menon P, Nguyen P. India's Integrated Child Development Services programme; equity and extent of coverage in 2006 and 2016. *Bull World Health Organ*. Apr 1 2019;97(4):270-282. doi:10.2471/blt.18.221135

3. Bile K, Shadoul A, Raaijmakers H, Altaf S, Shabib K. Learning through crisis: development and implementation of a health cluster strategy for internally displaced persons. *EMHJ-Eastern Mediterranean Health Journal, 16 (Supp), 82-90, 2010*. 2010;

4. Bile K, Hafeez A, Kazi G, Southall D. Protecting the right to health of internally displaced mothers and children: the imperative of inter-cluster coordination for translating best practices into effective participatory action. 2011;

5. Gowani S, Yousafzai AK, Armstrong R, Bhutta ZA. Cost effectiveness of responsive stimulation and nutrition interventions on early child development outcomes in Pakistan. *Ann N Y Acad Sci*. Jan 2014;1308:149-161. doi:10.1111/nyas.12367

6. Yousafzai AK, Rasheed MA, Rizvi A, Armstrong R, Bhutta ZA. Effect of integrated responsive stimulation and nutrition interventions in the Lady Health Worker programme in Pakistan on child development, growth, and health outcomes: a cluster-randomised factorial effectiveness trial. *The Lancet*. 2014;384(9950):1282-1293.

7. Ayoya MA, Golden K, Ngnie-Teta I, et al. Protecting and improving breastfeeding practices during a major emergency: lessons learnt from the baby tents in Haiti. *Bulletin of the World Health Organization*. 2013;91:612-617.

8. Olney DK, Pedehombga A, Ruel MT, Dillon A. A 2-year integrated agriculture and nutrition and health behavior change communication program targeted to women in Burkina Faso reduces anemia, wasting, and diarrhea in children 3-12.9 months of age at baseline: a cluster-randomized controlled trial. *J Nutr*. Jun 2015;145(6):1317-24. doi:10.3945/jn.114.203539

9. Leroy JL, Olney D, Ruel M. Tubaramure, a food-assisted integrated health and nutrition program in Burundi, increases maternal and child hemoglobin concentrations and reduces anemia: a theory-based cluster-randomized controlled intervention trial. *The Journal of nutrition*. 2016;146(8):1601-1608.

10. Leroy JL, Olney DK, Bliznashka L, Ruel M. Tubaramure, a food-assisted maternal and child health and nutrition program in Burundi, increased household food security and energy and micronutrient consumption, and maternal and child dietary diversity: a cluster-randomized controlled trial. *The Journal of nutrition*. 2020;150(4):945-957.

11. Ickes SB. Supportive Evidence for Program Impact Pathways: Food-Assisted Maternal and Child Health and Nutrition Programs Can Produce Sustained Dietary Improvements. *The Journal of Nutrition*. 2020;150(4):661.

12. Fenn B, Myatt M, Mates E, Black RE, Wilkinson C, Khara T. Effects on child growth of a reduction in the general food distribution ration and provision of small-quantity lipid-based nutrient supplements in refugee camps in eastern Chad. *BMJ Nutr Prev Health*. 2021;4(1):235-242. doi:10.1136/bmjnph-2021-000292

13. Fagerli K, O'Connor K, Kim S, et al. Impact of the Integration of Water Treatment, Hygiene, Nutrition, and Clean Delivery Interventions on Maternal Health Service Use. *Am J Trop Med Hyg*. May 2017;96(5):1253-1260. doi:10.4269/ajtmh.16-0709

14. Levin C, Self J, Kedera E, et al. What is the cost of integration? Evidence from an integrated health and agriculture project to improve nutrition outcomes in Western Kenya. *Health policy and planning*. 08/29 2019;34doi:10.1093/heapol/czz083

15. Bliss J, Golden K, Bourahla L, Stoltzfus R, Pelletier D. An emergency cash transfer program promotes weight gain and reduces acute malnutrition risk among children 6-24 months old during a food crisis in Niger. *Journal of global health*. 2018;8(1)

16. Drupadi Dillon DS, Dewi Fatmaningrum. *An Evaluation of the 2012 – 2015 Maternal &Child Nutrition (MCN) Program*. 2016:79. August 2016. <https://cdn.wfp.org/wfp.org/publications/1.%20MCN_Final%20evaluation%20report_WFP_SEAMEO.pdf>

17. Habib MA, Soofi S, Cousens S, et al. Community engagement and integrated health and polio immunisation campaigns in conflict-affected areas of Pakistan: a cluster randomised controlled trial. *The Lancet Global Health*. 2017;5(6):e593-e603.

18. Dozio E, Le Roch K, Bizouerne C. Baby friendly spaces: an intervention for pregnant and lactating women and their infants in Cameroon. *Intervention Journal of Mental Health and Psychosocial Support in Conflict Affected Areas*. 2020;18(1):78-84.

19. Head JR, Pachon H, Tadesse W, Tesfamariam M, Freeman MC. INTEGRATION OF WATER, SANITATION, HYGIENE AND NUTRITION PROGRAMMING IS ASSOCIATED WITH LOWER PREVALENCE OF CHILD STUNTING AND FEVER IN OROMIA, ETHIOPIA. Article. *African Journal of Food, Agriculture, Nutrition and Development*. 2019/08//

// 2019;19:14971+.

20. Fenn B, Colbourn T, Dolan C, Pietzsch S, Sangrasi M, Shoham J. Impact evaluation of different cash-based intervention modalities on child and maternal nutritional status in Sindh Province, Pakistan, at 6 mo and at 1 y: A cluster randomised controlled trial. *PLoS Med*. May 2017;14(5):e1002305. doi:10.1371/journal.pmed.1002305

21. Trenouth L, Colbourn T, Fenn B, Pietzsch S, Myatt M, Puett C. The cost of preventing undernutrition: cost, cost-efficiency and cost-effectiveness of three cash-based interventions on nutrition outcomes in Dadu, Pakistan. *Health Policy Plan*. Jul 1 2018;33(6):743-754. doi:10.1093/heapol/czy045

22. Dr Sahib Jan Badar EAA, Dr Saba Shuja, Dr Wisal Khan, Dr Umar Khan. Combining WASH and nutrition activities within a multisectoral package to improve young children’s diets and reduce child stunting in Sindh province, Pakistan. Emergency Nutrition Network (ENN); 2020(2):19. doi:Enn_6690 Pakistan. Accessed 29/06/2020 00:00:00. <https://www.ennonline.net/nex/southasia/2/pakistansindh>

23. WFP. *WFP Nigeria Country Brief, November 2016*. 2016. 30 Nov 2016. <https://reliefweb.int/report/nigeria/wfp-nigeria-country-brief-november-2016>

24. Monica Shrivastav AS, Neha Abraham, R.S. Reshmi, Sarita Anand, Apolenarius Purty, Rika Shalima Xaxa, Jagjit Minj, Babita Mohapatra, Sethi V. Early lessons from Swabhimaan, a multi-sector integrated health and nutrition programme for women and girls in India. Emergency Nutrition Network (ENN); 2021(65):103. doi:Enn_7100 India. Accessed 20/05/2021 00:00:00. <https://www.ennonline.net/fex/65/healthnutritionprogrammewomenindia>

25. Marshak A, Young H, Bontrager EN, Boyd EM. The Relationship Between Acute Malnutrition, Hygiene Practices, Water and Livestock, and Their Program Implications in Eastern Chad. *Food Nutr Bull*. Mar 2017;38(1):115-127. doi:10.1177/0379572116681682

26. Borja A, Khondaker R, Durant J, Ochoa B. Child-centred, cross-sectoral mental health and psychosocial support interventions in the Rohingya response: a field report by Save the Children. *Intervention Journal of Mental Health and Psychosocial Support in Conflict Affected Areas*. 2019;17(2):231-237.

27. Kurdi S, Figueroa JL, Ibrahim H. Nutritional training in a humanitarian context: evidence from a cluster randomized trial. *Maternal & Child Nutrition*. 2020;16(3):e12973.

28. Kurdi S, Ghorpade Y, Ibrahim H. *The Cash for Nutrition Intervention in Yemen Impact Evaluation Study*. 2019.

29. Dulacha D, Ramadan OPC, Guyo AG, et al. Use of mobile medical teams to fill critical gaps in health service delivery in complex humanitarian settings, 2017-2020: a case study of South Sudan. *The Pan African Medical Journal*. 2022;42(Suppl 1)

30. Fahmida U, Hidayat AT, Oka A, Suciyanti D, Pathurrahman P, Wangge G. Effectiveness of an Integrated Nutrition Rehabilitation on Growth and Development of Children under Five Post 2018 Earthquake in East Lombok, Indonesia. *Int J Environ Res Public Health*. Feb 28 2022;19(5)doi:10.3390/ijerph19052814

31. Ghattas H, Choufani J, Jamaluddine Z, Masterson AR, Sahyoun NR. Linking women-led community kitchens to school food programmes: lessons learned from the Healthy Kitchens, Healthy Children intervention in Palestinian refugees in Lebanon. *Public Health Nutrition*. 2020;23(5):914-923.

32. Sahyoun NR, Jamaluddine Z, Choufani J, Mesmar S, Reese-Masterson A, Ghattas H. A mixed-methods evaluation of community-based healthy kitchens as social enterprises for refugee women. *BMC public health*. 2019;19:1-11.

33. Grijalva-Eternod CS, Jelle M, Mohamed H, et al. Evaluation of conditional cash transfers and mHealth audio messaging in reduction of risk factors for childhood malnutrition in internally displaced persons camps in Somalia: A 2 × 2 factorial cluster-randomised controlled trial. *PLoS Med*. Feb 2023;20(2):e1004180. doi:10.1371/journal.pmed.1004180

34. Eveline Ngwenyi MJ, Nicolas Joannic, Patricia C. Addressing acute malnutrition in Cameroon during an emergency: Results and benefits of an integrated prevention programme. Emergency Nutrition Network (ENN); 2019(60):96. doi:fex.60.108 Cameroon. Accessed 03/07/2019 00:00:00. <https://www.ennonline.net/fex/60/acutemalnutritioncameroon>

35. Ahmad Nawid Qarizada MLG, Abdul Qadir Baqakhil, Goergen M. Integrating nutrition services into mobile health teams: Bringing comprehensive services to an underserved population in Afghanistan. Emergency Nutrition Network (ENN); 2019(61):62. doi:fex.61.407 Afghanistan. Accessed 04/11/2019 00:00:00. <https://www.ennonline.net/fex/61/mobilehealthteams>

36. Dr Khawaja Masuood Ahmed DSS, Khan DW. Providing maternal nutrition services at sub-national level in Punjab Province, Pakistan. Emergency Nutrition Network (ENN); 2019(1):23. doi:Enn_6338 Pakistan. Accessed 24/06/2019 00:00:00. <https://www.ennonline.net/nex/southasia/maternalnutritionpunjabprovince>

37. SaïdouTamboura DMI, Pantella L. Impact evaluation of WASH in nutrition intervention on morbidity and acute malnutrition in Niger. Emergency Nutrition Network (ENN); 2019(59):47. doi:Enn_6222 Niger. Accessed 26/01/2019 00:00:00. <https://www.ennonline.net/fex/59/washcmamniger>

38. Sreeparna Ghosh Mukherjee PS, Shah DNN. Ensuring pregnancy weight gain: An integrated community-based approach to tackle maternal nutrition in India. Emergency Nutrition Network (ENN); 2019(61):14. doi:fex.61.502 India. Accessed 14/11/2019 00:00:00. <https://www.ennonline.net/fex/61/pregnancyweightgain>

39. Group HDaEMIE. *What works: ETHIOPIA Nutrition Project*. 2019. June 26 2019. <https://documents1.worldbank.org/curated/en/276231563896790836/pdf/Ethiopia-Nutrition-Project.pdf>

40. El-Jor C, Rahi B, Malhame MEK, Mattar L, Moussa S, Zeeni N. Assessment of the World Food Programme summer camps in Lebanon: a model of effective interventions for vulnerable adolescents. *British Journal of Nutrition*. 2021;125(12):1416-1426.

41. Dr Mohammadullah Noorzad DPA, Maureen L. Gallagher. Scaling up a community-based nutrition package in Afghanistan to improve complementary feeding practices in children 6-23 months of age. Emergency Nutrition Network (ENN); 2020(2):16. doi:Enn_6685 Afghanistan. Accessed 29/06/2020 00:00:00. <https://www.ennonline.net/nex/southasia/2/afghanistan>

42. Alemu T, Bezabih B, Amsalu A, Hassen E, Haile M, Abite M. Health and nutrition emergency response among internally displaced persons at Ranch collective site, Chagni, Ethiopia: The role of emergency operation center, lessons from the field, and way forwards. *Front Public Health*. 2022;10:926551. doi:10.3389/fpubh.2022.926551

43. Frontier Economics FC, Nina Nepesova, Isis Sunwoo, Emma Wanchap. *Fiscal Year 2020 Results Report: THE COST OF CONFLICT FOR CHILDREN FIVE YEARS OF THE SYRIA CRISIS*. 2020:26. March 2016. <https://www.wvi.org/sites/default/files/The%20Cost%20of%20Conflict%20for%20Children%20report%20-%20online%20version.pdf>

44. Codjia P, Kutondo E, Kamudoni P, et al. Mid-term evaluation of Maternal and Child Nutrition Programme (MCNP II) in Kenya. *BMC Public Health*. 2022;22(1):2191.

45. Ahmed AS. *Integrated Humanitarian Assistance Program (IHAP) South and East Darfur, Final Evaluation - October 2020*. 2020:44. 31 Oct 2020. <https://reliefweb.int/report/sudan/integrated-humanitarian-assistance-program-ihap-south-and-east-darfur-final-evaluation>

46. SOYDA. *SOYDA Quarterly Progressive Narrative Report, October - December 2021*. 2021. *Quarterly Progressive Narrative Report*.

47. Tayalla Elmedani AAO. *Final Impact Evaluation Report: Impact Evaluation of the Integrated Humanitarian Assistance Project aiming to Reduce the Secondary Impacts of COVID-19 on the Most Vulnerable Populations in South and East Darfur - Sept. 2022, Khartoum*. 2022:109. 3 December 2022. <https://reliefweb.int/report/sudan/final-impact-evaluation-report-impact-evaluation-integrated-humanitarian-assistance-project-aiming-reduce-secondary-impacts-covid-19-most-vulnerable-populations-south-and-east-darfur-sept-2022-khartoum>

48. UNICEF. *Humanitarian Action for Children 2023 - Mali - Revision 1 (June 2023)*. 2023. 9 Jul 2023 <https://reliefweb.int/report/mali/humanitarian-action-children-2023-mali-revision-1-june-2023>

49. Renzaho A, Renzaho C. In the shadow of the volcanoes: the impact of intervention on the nutrition and health status of Rwandan refugee children in Zaire two years on from the exodus. *Nutrition & Dietetics*. 2003;60(2)

50. Gorstein J, Bhaskaram P, Khanum S, et al. Safety and impact of vitamin A supplementation delivered with oral polio vaccine as part of the immunization campaign in Orissa, India. *Food Nutr Bull*. Dec 2003;24(4):319-31. doi:10.1177/156482650302400402

51. Laillou A, Baye K, Zelalem M, Chitekwe S. Vitamin A supplementation and estimated number of averted child deaths in Ethiopia: 15 years in practice (2005–2019). *Maternal & child nutrition*. 2021;17(3):e13132.

52. Singh V, Ahmed S, Dreyfuss ML, et al. Non-governmental organization facilitation of a community-based nutrition and health program: Effect on program exposure and associated infant feeding practices in rural India. *PLoS One*. 2017;12(9):e0183316.

53. Singh V, Ahmed S, Dreyfuss ML, et al. An integrated nutrition and health program package on IYCN improves breastfeeding but not complementary feeding and nutritional status in rural northern India: A quasi-experimental randomized longitudinal study. *PLoS One*. 2017;12(9):e0185030. doi:10.1371/journal.pone.0185030

54. Bhandari N, Mazumder S, Taneja S, Sommerfelt H, Strand TA. Effect of implementation of Integrated Management of Neonatal and Childhood Illness (IMNCI) programme on neonatal and infant mortality: cluster randomised controlled trial. *Bmj*. Mar 21 2012;344:e1634. doi:10.1136/bmj.e1634

55. Mazumder S, Taneja S, Bahl R, et al. Effect of implementation of Integrated Management of Neonatal and Childhood Illness programme on treatment seeking practices for morbidities in infants: cluster randomised trial. *BMJ : British Medical Journal*. 2014;349:g4988. doi:10.1136/bmj.g4988

56. Doherty T, Chopra M, Tomlinson M, Oliphant N, Nsibande D, Mason J. Moving from vertical to integrated child health programmes: experiences from a multi-country assessment of the Child Health Days approach in Africa. *Trop Med Int Health*. Mar 2010;15(3):296-305. doi:10.1111/j.1365-3156.2009.02454.x

57. Taneja S, Bahl S, Mazumder S, Martines J, Bhandari N, Bhan MK. Impact on inequities in health indicators: Effect of implementing the integrated management of neonatal and childhood illness programme in Haryana, India. *J Glob Health*. Jun 2015;5(1):010401. doi:10.7189/jogh.05.010401

58. Mokori A, Hendriks S, Oriskushaba P, Oelofse A. Changes in complementary feeding practices and nutrition status in returnee children aged 6-23 months in northern Uganda. *South African Journal of Clinical Nutrition*. 2013;26(4):201-211.

59. Tadesse E, Worku A, Berhane Y, Ekström EC. An integrated community‐based outpatient therapeutic feeding programme for severe acute malnutrition in rural Southern Ethiopia: Recovery, fatality, and nutritional status after discharge. *Maternal & child nutrition*. 2018;14(2):e12519.

60. Richardson L, Bush, A. & Ambroso, G. *An Independent Review of UNHCR's Response to the Somali Refugee Influx in Dollo Ado, Ethiopia, 2011*. Program review. 2012. 01 Nov 2012. <https://www.unhcr.org/media/independent-review-unhcrs-response-somali-refugee-influx-dollo-ado-ethiopia-2011>

61. Sellen DS, Sharmin; Tefera, Bethlehem; Hyder, Ziauddin. *Strengthening Family Planning with Community-based Nutrition Interventions in Ethiopia : A Qualitative Study*. 2012. 2012-06. <https://openknowledge.worldbank.org/entities/publication/643943e1-742f-55bb-9e05-abe6fe1f9cc5>

62. Kim SS, Avula R, Ved R, et al. Understanding the role of intersectoral convergence in the delivery of essential maternal and child nutrition interventions in Odisha, India: a qualitative study. *BMC Public Health*. Feb 2 2017;17(1):161. doi:10.1186/s12889-017-4088-z

63. Soaade Messoudi AL, Anastasia Isyuk. *Philippines: Help for displaced persons and detainees in Zamboanga*. 2013. 05-10-2013 <https://www.icrc.org/en/doc/resources/documents/update/2013/10-05-philippines-idp-detainee.htm#:~:text=While%20continuing%20to%20distribute%20food,of%20people%20facing%20prolonged%20displacement>.

64. Mukhopadhyay DK, Sarkar AP, Chowdhury A, Gazi E, Sarkar GN. Can frontline workers be change agents for infant feeding and growth?-A community Trial. *Al Ameen J Med Sci*. 2017;10:71-77.

65. Deconinck H, Hallarou ME, Pesonen A, et al. Understanding factors that influence the integration of acute malnutrition interventions into the national health system in Niger. *Health Policy Plan*. Dec 2016;31(10):1364-1373. doi:10.1093/heapol/czw073

66. Chehab ET, Anya B-PM, Onyango AW, et al. Experience of integrating vitamin A supplementation into polio campaigns in the African Region. *Vaccine*. 2016;34(43):5199-5202.

67. UNICEF. *South Sudan Integrated Rapid Response Mechanism 2014–2016*. 2017. <https://reliefweb.int/report/south-sudan/south-sudan-integrated-rapid-response-mechanism-2014-2016#:~:text=To%20reach%20these%20populations%20with,to%20rapidly%20changing%20needs%20on>

68. Renzaho AMN, Dachi G, Tesfaselassie K, et al. Assessing the Impact of Integrated Community-Based Management of Severe Wasting Programs in Conflict-Stricken South Sudan: A Multi-Dimensional Approach to Scalability of Nutrition Emergency Response Programs. *Int J Environ Res Public Health*. Aug 29 2021;18(17)doi:10.3390/ijerph18179113

69. WFP-UNICEF. *The WFP-UNICEF Rapid Response Mechanism in South Sudan - One Year On: Results, Challenges and Way Forward - May 2015*.34. <https://www.unicef.org/southsudan/media/736/file/South-Sudan-WFP-UNICEF-RRM-2015.pdf>

70. Gatobu S, Horton S, Kiflie Aleyamehu Y, Abraham G, Birhanu N, Greig A. Delivering Vitamin A Supplements to Children Aged 6 to 59 Months: Comparing Delivery Through Mass Campaign and Through Routine Health Services in Ethiopia. *Food Nutr Bull*. Dec 2017;38(4):564-573. doi:10.1177/0379572117708657

71. Ouedraogo CT, Wessells KR, Young RR, et al. The mixed effects of a package of multilevel interventions on the health and care of pregnant women in Zinder, Niger. *BMJ Global Health*. 2019;4(6):e001200.

72. Oiye S, Safari N, Anyango J, et al. Programmatic implications of some vitamin A supplementation and deworming determinants among children aged 6-59 months in resource-poor rural Kenya. *Pan African Medical Journal*. 2019;32(1)

73. Wanzira H, Muyinda R, Lochoro P, et al. Quality of care for children with acute malnutrition at health center level in Uganda: a cross sectional study in West Nile region during the refugee crisis. *BMC Health Services Research*. 2018;18:1-10.

74. Doris Mwendwa JN, Korir J. Integrating MIYCN initiatives across sectors in Dadaab refugee camps in Kenya. Emergency Nutrition Network (ENN); 2016(51):65. doi:Enn_5081 Kenya. Accessed 19/01/2016 00:00:00. <https://www.ennonline.net/fex/51/integratingmiycndadaabkenya>

75. Dr. Saja Abdullah DRAA, Sharhan DR. Scaling up nutrition services and maintaining service during conflict in Yemen: Lessons from the Hodeidah sub-national Nutrition Cluster. Emergency Nutrition Network (ENN); 2016(52):57. doi:Enn_5224 Yemen. Accessed 10/05/2016 00:00:00. <https://www.ennonline.net/fex/52/nutritionservicesduringconflict>

76. Leidman E. Acute malnutrition among children, mortality, and humanitarian interventions in conflict-affected regions—Nigeria, October 2016–March 2017. *MMWR Morbidity and mortality weekly report*. 2017;66

77. Amsalu R, Morris CN, Hynes M, et al. Effectiveness of clinical training on improving essential newborn care practices in Bossaso, Somalia: a pre and postintervention study. *BMC Pediatrics*. 2020/05/13 2020;20(1):215. doi:10.1186/s12887-020-02120-x

78. Amsalu R, Schulte-Hillen C, Garcia DM, et al. Lessons Learned From Helping Babies Survive in Humanitarian Settings. *Pediatrics*. Oct 2020;146(Suppl 2):S208-s217. doi:10.1542/peds.2020-016915L

79. Shaker‐Berbari L, Ghattas H, Symon AG, Anderson AS. Infant and young child feeding in emergencies: organisational policies and activities during the refugee crisis in Lebanon. *Maternal & child nutrition*. 2018;14(3):e12576.

80. Oladeji O, Campbell P, Jaiswal C, et al. Integrating immunization services into nutrition sites to improve immunization status of internally displaced persons’ children living in Bentiu protection of civilian site, South Sudan. *Pan African Medical Journal*. 2019;32(1)

81. Oresanya O, Phillips A, Okereke E, et al. Co-implementing vitamin A supplementation with seasonal malaria chemoprevention in Sokoto State, Nigeria: a feasibility and acceptability study. *BMC Health Services Research*. 2022;22(1):871.

82. Bank W. *Livelihoods and Nutrition: A Women’s Empowerment and Convergence Initiative – JEEViKA*. <https://documents1.worldbank.org/curated/pt/109401572440521978/pdf/Livelihoods-and-Nutrition-A-Women-s-Empowerment-and-Convergence-Initiative-JEEViKA.pdf>

83. Coulibaly-Zerbo F, Al-Jawaldeh A, Prinzo ZCW, et al. Maintaining essential nutrition services to underfive children in Yemen: a programmatic adaptation amidst the COVID-19 pandemic. *Children*. 2021;8(5):350.

84. UNICEF. *Boosting breastfeeding: Bringing postnatal care to the doorstep in Gaza, State of Palestine, 2020*. 2020:12. *FIELD REPORTS: Lessons from improving nutrition at scale*. <https://www.unicef.org/media/94071/file/2020-Field-Report-State-of-Palestine.pdf>

85. Alliance GNCT. Supporting non-breastfed children as part of an Ebola response - Experiences from the Democratic Republic of the Congo. Emergency Nutrition Network (ENN); 2020(0):0. doi:Enn_6874 Global. Accessed 01/12/2020 00:00:00. <https://www.ennonline.net/gtamebolacasestudy>

86. Sarrassat S, Lewis JJ, Some AS, Somda S, Cousens S, Blanchet K. An Integrated eDiagnosis Approach (IeDA) versus standard IMCI for assessing and managing childhood illness in Burkina Faso: a stepped-wedge cluster randomised trial. *BMC Health Services Research*. 2021/04/16 2021;21(1):354. doi:10.1186/s12913-021-06317-3

87. Somalia Nutrition Cluster IOfMI. *Report of Mass MUAC Screening in Bardhere and Dollow December 2022*. 2022. 1 Feb 2023. <https://reliefweb.int/report/somalia/report-mass-muac-screening-bardhere-and-dollow-december-2022>

88. WRC WsRC. *Localizing Humanitarian Aid: Learning from a Consortium-Based Approach to Designing and Implementing a Village Health Worker Program in Borno State, Nigeria*. 2023. 17 Apr 2023. <https://reliefweb.int/report/nigeria/localizing-humanitarian-aid-learning-consortium-based-approach-designing-and-implementing-village-health-worker-program-borno-state-nigeria>

89. Morris J, Jones L, Berrino A, Jordans MJ, Okema L, Crow C. Does combining infant stimulation with emergency feeding improve psychosocial outcomes for displaced mothers and babies? A controlled evaluation from northern Uganda. *American Journal of Orthopsychiatry*. 2012;82(3):349-357.

90. Scott J, Marquer C, Berthe F, Ategbo E-A, Grais RF, Langendorf C. The gender, social and cultural influences on the management and use of unconditional cash transfers in Niger: a qualitative study. *Public health nutrition*. 2017;20(9):1657-1665.

91. Langendorf C, Roederer T, de Pee S, et al. Preventing acute malnutrition among young children in crises: a prospective intervention study in Niger. *PLoS medicine*. 2014;11(9):e1001714.

92. Sibson VL, Grijalva-Eternod CS, Noura G, et al. Findings from a cluster randomised trial of unconditional cash transfers in Niger. *Matern Child Nutr*. Oct 2018;14(4):e12615. doi:10.1111/mcn.12615

93. McGrath M, Schafer A. Integrating psychosocial support into nutrition programmes in West Africa during the Sahel food crisis. *Intervention*. 2014;12(1):115-126.

94. Fernandez-Rao S, Hurley KM, Nair KM, et al. Integrating nutrition and early child-development interventions among infants and preschoolers in rural India. *Ann N Y Acad Sci*. Jan 2014;1308:218-231. doi:10.1111/nyas.12278

95. Hashmi A, Carrara VI, Nyein PB, Darakamon MC, Charunwatthana P, McGready R. The healthy baby flipbook: piloting home-based counseling for refugee mothers to improve infant feeding and water, sanitation, and hygiene (WASH) practices. *Global Health Action*. 2019;12(1):1560115.

96. Grellety E, Babakazo P, Bangana A, et al. Effects of unconditional cash transfers on the outcome of treatment for severe acute malnutrition (SAM): a cluster-randomised trial in the Democratic Republic of the Congo. *BMC Med*. Apr 26 2017;15(1):87. doi:10.1186/s12916-017-0848-y

97. Renuka Bery ST, Shafritz L. WASHplus in Mali: integrating WASH and nutrition for healthy communities. Emergency Nutrition Network (ENN); 2016(51):134. doi:Enn_5105 Mali. Accessed 28/01/2016 00:00:00. <https://www.ennonline.net/fex/51/washplusmali>

98. Mike Brewin HK, Kelly David, Abdulmajid Khojali, Nuha Mohamed, Nathan Horst. *Sudan PRRO 200808 "Support for Food Security and Nutrition for Conflict-Affected and Chronically Vulnerable Populations": A mid-term Operation Evaluation*. 2017. 10 June 2016. <https://www.wfp.org/publications/sudan-prro-200808-support-food-security-and-nutrition-conflict-affected-and-chronically-vule>

99. Locks LM, Nanama S, Addo OY, et al. An integrated infant and young child feeding and small‐quantity lipid‐based nutrient supplementation programme in the Democratic Republic of Congo is associated with improvements in breastfeeding and handwashing behaviours but not dietary diversity. *Maternal & child nutrition*. 2019;15(3):e12784.

100. Addo OY, Tripp K, Nanama S, et al. An Integrated Infant and Young Child Feeding and Small-Quantity Lipid-based Nutrient Supplementation Program Is Associated with Improved Gross Motor and Communication Scores of Children 6-18 Months in the Democratic Republic of Congo. *The Journal of pediatrics*. 2020;222:154-163.

101. Ali MK, Flacking R, Sulaiman M, Osman F. Effects of Nutrition Counselling and Unconditional Cash Transfer on Child Growth and Family Food Security in Internally Displaced Person Camps in Somalia-A Quasi-Experimental Study. *Int J Environ Res Public Health*. Oct 18 2022;19(20)doi:10.3390/ijerph192013441

102. Soofi SB, Ariff S, Khan GN, et al. Effectiveness of unconditional cash transfers combined with lipid-based nutrient supplement and/or behavior change communication to prevent stunting among children in Pakistan: a cluster randomized controlled trial. *Am J Clin Nutr*. Feb 9 2022;115(2):492-502. doi:10.1093/ajcn/nqab341

103. Thumbi SM, Muema J, Mutono N, et al. The Livestock for Health Study: A Field Trial on Livestock Interventions to Prevent Acute Malnutrition Among Women and Children in Pastoralist Communities in Northern Kenya. *Food Nutr Bull*. Dec 2023;44(2_suppl):S119-s123. doi:10.1177/03795721231195427

104. Amsalu R, Schulte-Hillen C, Martinez Garcia D, et al. Lessons Learned From Helping Babies Survive in Humanitarian Settings. *Pediatrics*. 10/08 2020;146doi:10.1542/peds.2020-016915L

105. Fahmida U, Hidayat AT, Oka AASI, Suciyanti D, Pathurrahman P, Wangge G. Effectiveness of an integrated nutrition rehabilitation on growth and development of children under five post 2018 earthquake in East Lombok, Indonesia. *International Journal of Environmental Research and Public Health*. 2022;19(5):2814.

106. Ghattas H, Choufani J, Jamaluddine Z, Masterson AR, Sahyoun NR. Linking women-led community kitchens to school food programmes: lessons learned from the Healthy Kitchens, Healthy Children intervention in Palestinian refugees in Lebanon. *Public Health Nutr*. Apr 2020;23(5):914-923. doi:10.1017/s1368980019003161
